# Nanoporous anodic alumina-based gas sensors: insights into advances and perspectives

**DOI:** 10.1007/s00604-025-07234-6

**Published:** 2025-06-23

**Authors:** Khoa Nhu Tran, Huong Nguyen Que Tran, Andrew D. Abell, Cheryl Suwen Law, Abel Santos

**Affiliations:** 1 School of Chemical Engineering, The University of Adelaide, Adelaide, South Australia 5005 Australia; 2 Institute for Photonics and Advanced Sensing, The University of Adelaide, Adelaide, South Australia 5005 Australia; 3 Department of Chemistry, The University of Adelaide, Adelaide, South Australia 5005 Australia

**Keywords:** Nanoporous anodic alumina, Gas sensors, Electrochemical sensors, Optical sensors, Nanostructured materials, Multiplex sensing

## Abstract

Achieving high-performance gas sensing requires materials and transduction mechanisms that enhance sensitivity, selectivity, and stability, while addressing challenges such as cross-sensitivity and real-time operation. Conventional sensor platforms often involve trade-offs between response time, detection limits, and environmental robustness. Nanoporous anodic alumina (NAA) fabricated by electrochemical oxidation—anodization—of aluminum provides a tunable platform for engineering gas sensors with tailored structural and physicochemical properties, enabling diverse transduction mechanisms and sensor configurations. This review categorizes NAA-based gas sensors into two major groups: electrochemical and optical sensors. The distinct interplay between the nanoporous architecture of NAA and its dielectric properties enhances charge transport in electrochemical sensors while enabling precise optical confinement and modulation in optical sensing platforms. Ongoing efforts in structural modifications, surface functionalization, and hybrid material integration continue to refine the capabilities of NAA-based gas sensors. Tailored nanostructured coatings, such as functionalized metal oxides, polymer composites, and plasmonic nanostructures, present new pathways for improving sensitivity and selectivity. The integration of data-driven signal processing, including machine learning-assisted analysis, is transforming how sensor responses are interpreted, endowing gas sensors with enhanced discrimination and multiplex sensing capabilities. These advancements, combined with innovations in microfabrication and miniaturized sensor arrays, enable new forms of NAA-based gas sensors. This review provides an up-to-date overview of recent progress and emerging directions in the development of NAA-based gas sensing technologies.

**Graphical Abstract:**

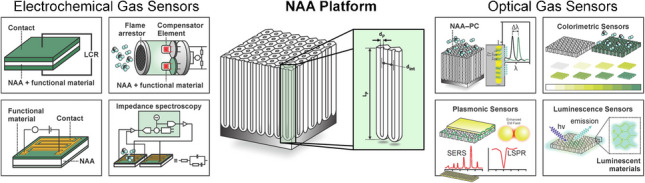

## Introduction

Gas sensors are indispensable tools across various sectors, including environmental monitoring, industrial safety, healthcare diagnostics, and air quality assessment, because of their role in detecting and analyzing gases critical to these fields [1–4]. Typically, a gas sensor is composed of an active layer for the detection of gas molecules and a transducer, which transforms distinct signals into readable formats. The primary applications of gas sensors are detecting hazardous gases, pollutants, and volatile organic compounds (VOCs), which have implications for human health, environmental sustainability, and industrial safety. For instance, gas sensors enable the detection of greenhouse gases and toxic emissions in environmental monitoring [5, 6], protect workers from harmful exposure in industrial settings [7], and facilitate non-invasive diagnostics in healthcare [8, 9]. With growing concerns about air pollution, climate change, and public health, there is raising demand for gas sensors that operate reliably in complex, multi-gas environments and that can detect trace concentrations of target analytes, with high precision [10, 11]. But existing gas sensing technologies face challenges to meet the increasingly demanding requirements of these applications. For example, fast, real-time gas sensing systems that can provide immediate data are essential for rapid, informed decision-making in fields such as environmental monitoring, defense, and industrial safety. Modern gas sensors must also be capable of distinguishing between various gases within complex matrices for accurate identification of target analytes. The importance of developing miniaturized, low-power, and compact gas sensors has also grown, especially for portable and wearable applications, where energy efficiency and device size are critical parameters. These demands drive the pursuit of research and innovation in sensor design, aiming to achieve the precision, responsiveness, and functionality required across diverse applications.

To address these challenges, researchers have increasingly turned to nanomaterials to enhance gas sensor performance and harness a range of transduction mechanisms. Because of their high surface area, tunable structures, and unique chemical and physical properties, nanomaterials are particularly well-suited for gas sensing applications, enabling enhanced interactions with gas molecules even at trace level concentrations [12]. Some examples of nanomaterials used in gas sensing include metal oxides, carbon-based nanomaterials, two- and three-dimensional materials, and nanoporous structures—each offering distinct benefits. In particular, nanoporous materials prepared by self-ordering synthesis based on electrochemical oxidation of metals have proven to have promising potential for gas sensing devices [13–15]. These structures, featuring arrays of nanopores arranged in a highly ordered manner with a vertically aligned configuration, improve sensitivity and reliability of sensing devices by controlling interactions with target gas molecules when these flow along their nanopores. Of all nanoporous materials, nanoporous anodic alumina (NAA) stands out for its highly ordered nanopore structure and tailorable characteristic properties to detect specific gases. The structure of NAA provides this platform material a qualitative advantage over other systems since it facilitates efficient mass transport of gas molecules and enhance the accessibility of molecular species to functional binding sites within high-aspect ratio nanopores. These features increase sensitivity and improve detection limits in gas sensing applications, harnessing a variety of transduction mechanisms (e.g., optical, electrical) [16]. Anodization is a highly versatile nanofabrication approach by which the structural features of the resultant NAA films can be precisely engineered for specific gas sensing applications [17, 18]. However, NAA is a chemically inert material based on anodic alumina, which does not have intrinsic chemical functionality for chemical selectivity. This constraint can be overcome by functionalizing the inner surface of the nanopores with a variety of functional groups and molecules through diverse techniques (e.g., chemical vapor deposition, atomic layer deposition, and solution-based approaches). These functional coatings based on nanoparticles, films, or self-assembled monolayers of molecules provide tailorable chemical groups for selective interactions with specific gas molecules [19, 20]. NAA platforms for gas sensing can also be combined with a variety of transduction mechanisms such as electrical and optical sensing technologies. Some examples of NAA electrical transduction include chemiresistive, capacitive, or catalytic responses induced by gas interactions with functional coatings in NAA films [21]. In contrast, the intrinsic optical properties of NAA make it an ideal platform to harness light–matter interactions for sensing applications. These systems can use a range of optical transduction mechanisms such as photoluminescence, surface enhanced Raman, localized surface plasmon resonances and interferometry [22].

**Fig. 1:**
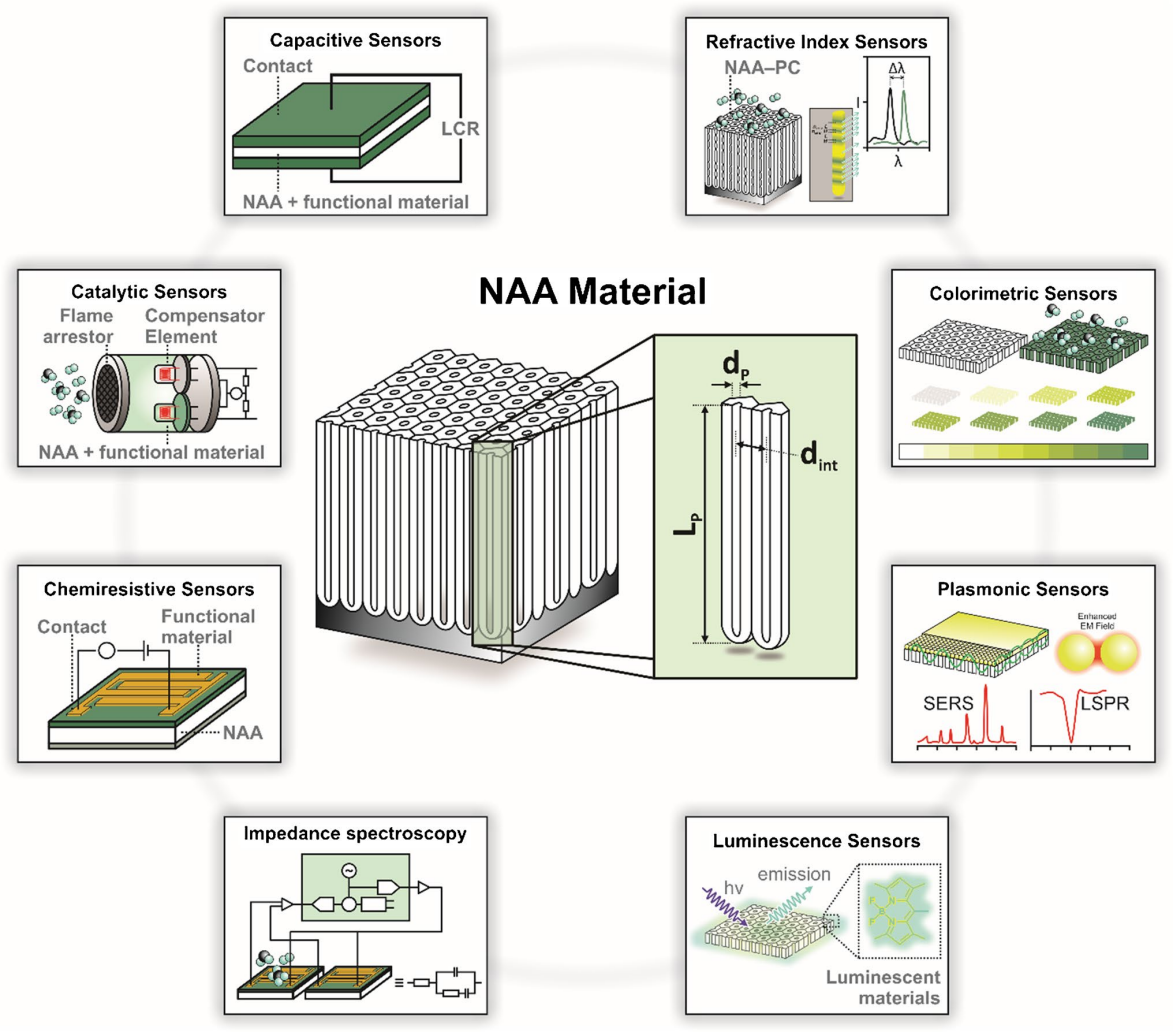
Overview of nanoporous anodic alumina (NAA) platforms and their applications in gas sensing, with illustrations of the main types of NAA-based gas sensors. NAA-based gas sensors using electrical transduction methods (left): capacitive sensor, catalytic sensor, chemiresistive sensor, and impedance spectroscopy-based sensors. NAA-based gas sensors using optical transduction methods, including photonic crystals (NAA–PC) (right): refractive index-based sensors, colorimetric sensor, plasmonic sensor, and luminescence sensor

In this context, this review provides an in-depth overview of recent advances and applications of NAA-based gas sensors (Fig. 1), with a focus on both electrical and optical transduction detection methods. The review starts with a brief introduction to NAA fabrication, including how a judicious selection of anodization conditions—voltage/current density profile, electrolyte composition and temperature, and anodization regime—enable a precise approach to engineer the structure of NAA for gas sensing applications. Next, we provide an overview of existing functionalization methods to tailor-engineer the chemical and physical properties of NAA platforms for gas sensing. We also provide detailed commentary on recent advances in NAA-based gas sensors, from pioneering to state-of-the-art studies. The review concludes with a general overview and a prospective outlook on emerging research directions and potential applications of NAA platforms in the field of gas sensing.

## Fabrication and properties of NAA

### Fabrication and structural engineering of NAA

**Fig. 2:**
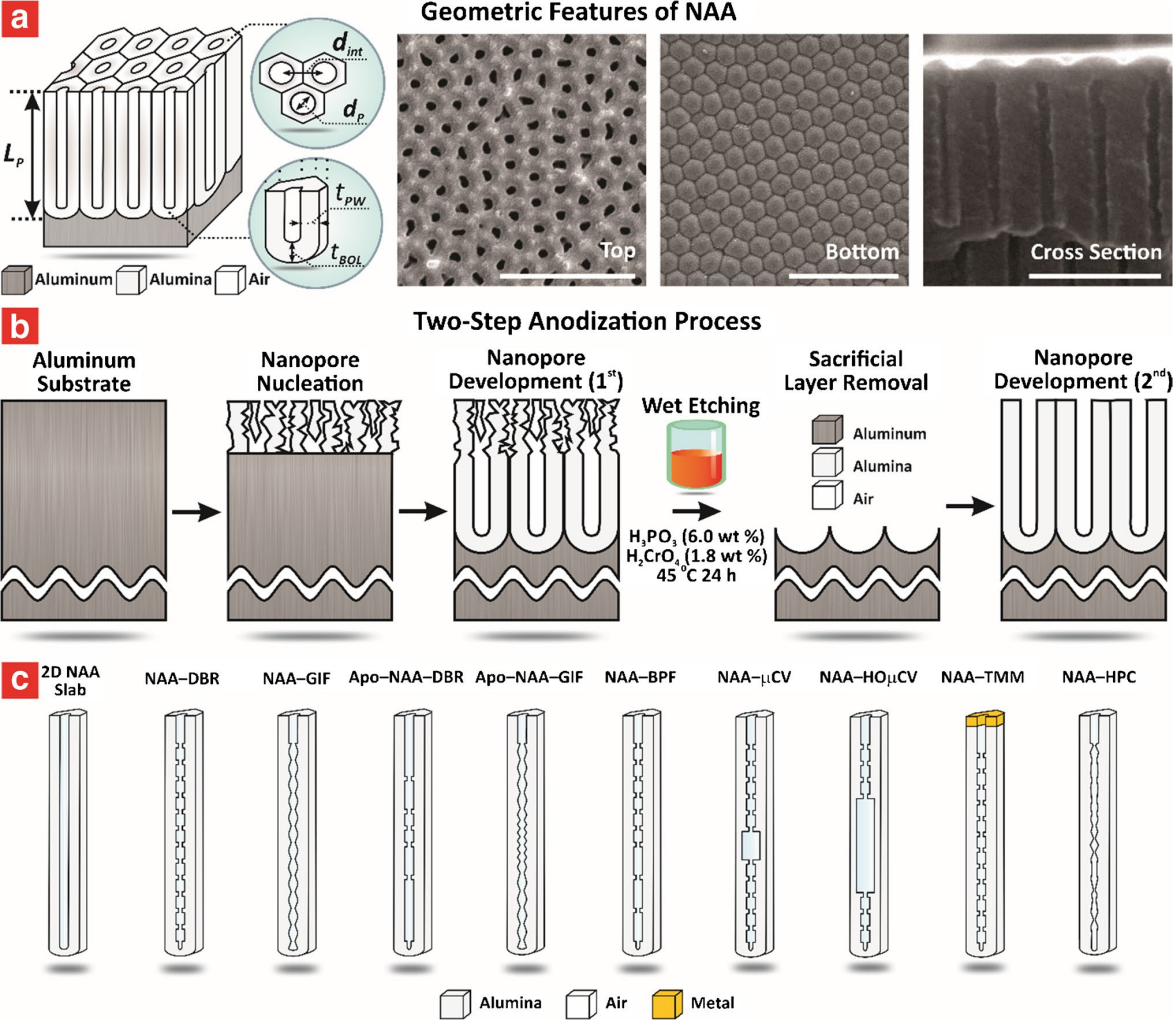
NAA fabrication and structures. **a** Illustration of NAA structure, with key geometric features: pore length (*L*_*P*_), interpore distance (*d*_*int*_), pore diameter (*d*_*P*_), barrier oxide layer thickness (*t*_*BOL*_), and thicknesses of pore wall and (*t*_*pw*_); and top, bottom, and cross-sectional field emission scanning electron microscopy (FEG-SEM) images of actual NAA platforms produced by two-step anodization in 0.3 M oxalic acid electrolyte at 40 V and 6 °C (scale bars from left to right—500, 500, and 250 nm). **b** Two-step anodization process flow: (i) starting with aluminum substrate; (ii) nucleation and (iii) growth of nanopores during the first anodization step; (iv) removal of sacrificial alumina layer by wet chemical etching; and (v) second anodization step for organized nanopore growth. (reproduced from ref. [23] with permission from the authors, copyright 2023). **c** Idealized illustrations of the most representative nanoporous anodic alumina photonic crystal (NAA–PC) structures produced by anodization to date (i.e., distributed Bragg reflector NAA–DBR; gradient-index filter NAA–GIF; apodized distributed Bragg reflector Apo–NAA–DBR; apodized gradient-index filter Apo–NAA–GIF; bandpass filter NAA–BPF; microcavity NAA–mCV; high-order microcavity NAA–HOmCV; Tamm plasmon cavity NAA–TMM; hybrid photonic crystal NAA–HPC) (reproduced from ref. [42] with permission from the authors, copyright 2025)

Structurally, NAA comprises a matrix of anodic alumina featuring arrays of straight cylindrical nanopores aligned perpendicularly to the aluminum substrate, each capped by closed hemispherical tips at their bottom. Nanopores are centered within hexagonally arranged cells [17]. NAA is typically fabricated through an electrochemical process known as anodization, which is extensively used in the metal finishing industry. In this fabrication approach, an aluminum substrate is immersed in an acidic electrolyte and subjected to a controlled voltage or current density input. Under specific conditions, there is a dynamic balance between the formation of aluminum oxide at the metal–electrolyte interface and the electric-field enhanced dissolution of anodic oxide at the oxide–electrolyte interface [16]. This balance between formation and dissolution of anodic oxide drives the growth of cylindrical nanopores perpendicular to the aluminum substrate. Judicious modification of the anodization parameters provides an effective approach to fine-tune the geometry of NAA nanopores (Fig. 2a), which is typically defined by structural parameters such as the pore diameter (*d*_*P*_), the interpore distance (*d*_*int*_), the pore length (*L*_*P*_), and the barrier layer thickness (*t*_*BOL*_) [23]. The typical ranges of these structural features are from 10 to 400 nm for *d*_*P*_, from 50 to 600 nm for *d*_*int*_, and from nanometers to several hundred micrometers for *L*_*P*_ [16, 24]. The resultant structure of NAA can be further tailored by post-anodization treatments such as pore widening or chemical etching to remove the barrier oxide layer (BOL) and produce through-hole NAA membranes.

Conventionally, NAA structures featuring straight cylindrical nanopores can be produced by the two-step anodization process pioneered by Masuda and co-workers, as shown in Fig. 2b [25–27]. This simple process enables the fabrication of self-organized NAA structures featuring highly ordered nanopores. The initial anodization step creates an oxide layer featuring disorganized nanopores at its top surface. These nanopores, which nucleate randomly across the surface of the anodic film, self-organize through a mechano–electrochemical process as the thickness of the nanofilm increases during the top–down anodization process [18, 28]. This process creates an imprinted pattern on the aluminum substrate with highly ordered hemispherical concavities replicating the bottom side of the NAA film. The resultant NAA film is then removed through wet chemical etching to pre-pattern the aluminum surface. The second anodization step proceeds under the same conditions as the initial step, enabling the nucleation and growth of nanopores at the center of each concave template due to localized electric field concentration. This guided growth mechanism leads to the formation of self-ordered cylindrical nanopores that extend uniformly from the surface to the bottom of the anodic film, and which grow perpendicularly aligned to the aluminum substrate without the need for complex lithographic methods [18]. The characteristic geometry of NAA nanopores can be finely tuned by the anodization conditions (i.e., electrolyte composition and temperature, anodization time, input voltage and current density, anodization regime) and post fabrication treatments (i.e., pore widening by wet chemical etching, annealing) [16–18, 29]. Common electrolytes used to fabricate NAA structures such as sulfuric, oxalic, and phosphoric acids provide well-established conditions to tune the characteristic structural features of NAA across a broad range of geometries. For example, *d*_*int*_ for NAA produced using these three electrolytes is typically 63, 100, and 500 nm, respectively, while *d*_*P*_ ranges from 10 to 25 nm for sulfuric acid, from 25 to 80 nm for oxalic acid, and > 100 nm for phosphoric acid [24, 30–36]. Other less commonly used electrolytes, such as citric, maleic, malonic, tartaric, and sulfamic acids, offer specialized advantages that influence pore morphology, in particular *d*_*P*_ and *d*_*int*_, over a wider geometric region [37–41]. By selecting and modifying the type of electrolyte, the anodization process can be fine-tuned to produce NAA platforms with tailored porosity and structural properties to support diverse applications that require specific pore configurations.

Other anodization approaches such as pulse and multi-step anodization enable new degrees of freedom to engineer the structure of nanopores in NAA by judiciously induced modulations of nanopore diameter along the anodic film thickness. By adjusting the input voltage or current density, or changing the acid electrolyte between anodization steps, diverse pore configurations such as gradient or layered NAA structures can be created [42, 43]. The BOL—hemispherical layer of electrically insulator anodic oxide—at the nanopore bottom tips poses certain limitations to directly translate modifications of input voltage or current density into pore diameter modulations. Some of these include uncontrollable oscillatory kinetic behavior under hard anodization regime and input–output time delay associated with the ionic diffusion-controlled growth/dissolution of anodic oxide. However, a careful design of pulsed profiles and anodization conditions (i.e., choice of acid electrolyte and temperature) has been demonstrated as an effective approach to overcome these constraints and enable the fabrication of new forms of NAA platforms [44–46]. Pulse-like anodization is characterized by a periodic change of input anodizing voltage or current density through time or charge density. These oscillations in the electric field-driven growth of anodic oxide result in periodic modulations of nanopore diameter along the nanopore length, from top to bottom of the anodic film [47–49]. This process takes advantage of the existing proportionality correlation between diameter of nanopores in NAA and anodization voltage or current density [50, 51]. By using distinct pulse anodization profiles (e.g., stepwise, sawtooth, Gaussian-like, sinusoidal, apodized sinusoidal), a broad variety of nanopore modulations following specific patterns can be generated in NAA structures to achieve stacking or overlaying architectures with different internal porosities [52–59]. Another strategy to generate NAA with asymmetrical nanopore architectures such as hierarchical [60–63], funnel-like [64–69], and multilayer [70–74] is the use of multi-step anodization, which combines several anodization steps at different anodizing conditions and/or pore widening treatment to control the porosity in multiple dimensions. A collection of representative idealized NAA-based photonic crystal (NAA–PC) structures fabricated with various anodization profiles are shown in Fig. 2c.

The versatility of anodization process stems from the combination of this simple nanofabrication with a range of post-fabrication modifications to broaden the potential of NAA structures. For example, the BOL of NAA film and the remaining aluminum substrate can be selectively removed after fabrication to obtain free-standing, through-hole NAA films (membranes) via wet chemical etching or dry etching (e.g., reactive ion etching, ion milling). Heat treatments can also be performed to enhance the structural stability and chemical resistance of NAA. High-temperature annealing converts amorphous alumina into crystalline alpha-alumina (α-Al_2_O_3_), significantly improving its mechanical durability and chemical stability for harsh chemical environments [18, 75, 76]. In summary, the broad variety of anodization strategies and post-treatment processes provide researchers with significant flexibility to control the structural, physical, and chemical properties of NAA platforms to meet specific needs, demonstrating the versatility and potential of this nanomaterial.

### Physical properties of NAA for gas sensing applications

NAA is a mechanically robust and hard oxide known for its exceptional thermal stability to withstand high temperatures. These features make NAA platforms ideal for use in gas sensors for high temperatures and reactive gases. However, while its structure allows for applications such as pressure sensors due to some inherent flexibility, NAA is highly susceptible to fracture under sudden or high-impact forces due to its ceramic nature. In terms of chemical composition, NAA consists of dielectric anodic aluminum oxide with an onion-like distribution of ionic impurities and vacancies from the outer to the inner side of the nanopore wall and BOL (i.e., oxide–electrolyte and metal–oxide interfaces, respectively). Two main regions can be distinguished in the nanopore structure of NAA: (i) an inner layer close to the aluminum–alumina interface mainly composed of pure alumina; and (ii) an outer layer located between the inner layer and the alumina–electrolyte interface, which contains impurities of anionic species incorporated from the acid electrolyte (e.g., sulfate, oxalate, and phosphate) during the anodization process [16, 17, 77]. The distribution and concentration of anionic impurities across the structure of anodic alumina is affected by the anodizing voltage or current density, and temperature, type, and concentration of electrolyte [78, 79]. Another important characteristic of NAA platforms is that these can be integrated into micro- and nanoelectromechanical systems (MNEMS) devices using current microfabrication methods. In these integrated systems, NAA can act not only as an active sensing layer, but also as a container to accommodate binding events inside its nanopores.

In terms of chemical characteristics, NAA combines the intrinsic properties of alumina with the advantages conferred by its porous structure, making it ideal for sensing applications. The chemical stability and inertness of alumina ensure reliable, long-term use in various environments. The high surface area and aspect ratio provided by its porous structure enhances sensitivity by increasing interactions with analytes over an extended area in a confined space. This porous nature also offers good adsorption properties, crucial for detecting gases and biomolecules. Another unique characteristic of NAA is its capability to adjust the surface chemistry along the inner side of its nanopore walls in a controlled manner by surface functionalization. This approach makes it possible to immobilize specific molecules to attain chemical selectivity for specific sensing applications. While alumina is an electrical insulator, its electrochemical properties can be modified with functional coatings based on semiconductors and conductors (e.g., metals, metal oxides, conductive polymers), broadening its use in hybrid sensor designs. The presence of hydroxyl groups on the inner surface of NAA, which are generated by heterolytic dissociation of water molecules at the oxide-electrolyte interface during the anodization process, enables anchor points for the binding of different functional molecules to design systems with desirable functionalities. These systems exhibit properties such as electron transfer, impedance, and variable resistance, which facilitate transduction mechanisms for gas sensing by enabling responses such as changes in current, impedance, and conductance upon interaction with target gases.

In terms of optical characteristics, the most attractive property of NAA is its composite structure made of air and alumina (RIU ~ 1.70), which can be precisely engineered as an effective medium to modulate light–matter interactions. 1D and 3D NAA–PCs such as distributed Bragg reflectors, gradient-index filters, and optical microcavities can be realized by distinct forms of pulse-like anodization [80–82]. Recent advances in this anodization technology have made it possible to expand the use of NAA optical platforms across gas sensing applications. The intrinsic photoluminescence of NAA is another valuable property for gas sensing. Photoluminescence in NAA structures is associated with ionized oxygen vacancies (F^+^ centers) and impurities incorporated into the structure of this anodic oxide during anodization [83, 84]. The photoluminescence emission of NAA is characterized by a broad, Gaussian-like band located at wavelengths ~ 350 nm. The intensity and wavelength of photoluminescence can be finely tuned via fabrication parameters such as voltage, current density, and electrolyte type and concentration, and structural designs to suit spectral range of specific gas sensing applications [85–87].

### Functionalization techniques for tailoring the properties of NAA

Several comprehensive reviews on the topic of NAA fabrication and functionalization methods have been published [17, 18, 22, 88]. Therefore, in this review, we will briefly introduce functionalization techniques used to modify the properties of NAA platforms to the extent needed for understanding distinct NAA-based gas sensing systems. The surface of as-produced NAA structures has been functionalized through a number of different surface modifications. These can be broadly categorized into soft techniques (e.g., molecular self-assembly, layer-by-layer deposition, dip coating, sol–gel) and hard techniques (e.g., plasma polymerization, atomic layer deposition (ALD), chemical vapor deposition (CVD), physical and electrochemical metal deposition) [89–92]. Through these methods, the large specific surface area of NAA can be activated for interacting with or capturing analyte gas molecules, and subsequently analyzed and interrogated by optical or electrochemical transduction mechanisms. On some occasions, these modifications also provide new means of tuning the optical, chemical, electrical, and electrochemical properties of NAA structures. These includes attributes such as reflectivity, hydrophobicity or hydrophilicity, antifouling properties, and the ability to host and preserve biomolecules for specific, high-throughput assays, offering versatility for developing advanced sensing and biosensing devices capable of multi-analyte detection [93, 94].

## Electrochemical sensors

Electrochemical gas sensors detect and quantify gas molecules by converting chemical interactions into electrical signals such as changes in current, voltage, capacitance, or resistance. This conversion, known as electrochemical signaling, allows for precise and rapid monitoring of target gases as the molecules interact with the sensing layer. When these sensors are developed using NAA platforms, its inherent nanoporous structural features (e.g., large surface area and adsorption sites, high-density ordered nanopores, and adjustable porosity) provide an effective material for gas sensing. These characteristics enhance interactions between gas molecules because of the extended surface area and the high aspect ratio of NAA’s nanopores, through which gas molecules must diffuse. Additionally, the electrochemical properties of NAA-based sensors can be customized and enhanced through functionalization. This enables NAA platforms to support various active materials, improving the selectivity and response capabilities of the composite system, while maintaining its inherent stability and adaptability.

With growing demands for compact, energy-efficient, and multifunctional gas sensors, current research has shifted toward leveraging nanotechnology advancements, including MNEMS technologies for sensor miniaturization. NAA-based gas sensors align with these trends due to their adaptable nanoporous structure and ability to support advanced functional layers, making them highly suitable for portable and real-time monitoring applications. In the field of electrochemical gas sensors, NAA-based sensors can be categorized by their detection principles, which define how they translate chemical reactions into electrical signals. The primary types include (i) capacitive, (ii) catalytic, (iii) chemiresistive, and (iv) impedance spectroscopy-based. In the subsequent sections, we will review these types of NAA-based electrochemical gas sensors, detailing key examples, their mechanisms, and comparative performance metrics.

### Capacitive sensors

Solid-state capacitive sensors based on NAA have garnered significant attention for their capability to detect various gases and vapors. The primary transduction mechanism of these sensors relies on changes in capacitance induced by the interaction between gas molecules and the intrinsic dielectric properties of NAA or the function materials coating its surface. This interaction modifies the dielectric constant of the active sensing layer, leading to detectable capacitance variations, which can subsequently be quantified to establish a correlation between analyte concentration and signal shift. The fundamental structure of these sensors involves a sandwich-like configuration with electrodes deposited on the top and bottom side of the NAA layer, which serves as the dielectric medium. In these systems, NAA has a through-hole membrane structure, and this arrangement allows gas molecules to permeate the nanopores and contact with the large active surface area within the nanopores, affecting the capacitance through adsorption-induced dielectric changes. Table 1 summarizes the most representative studies in this field.

**Table 1:** Summary of NAA-based capacitive sensors

Sensor design/chemical modification*	Detected gases**	Sensing mechanism	Ref
BOL removed—flow-through NAA membrane Au/NAA/Au multilayer: Au films deposited via sputtering	Humidity (10–85%RH) Methanol, ethanol, isobutane, SF_6_, CO_2_, N_2_, He (0–100%P/P_0_)	Adsorbed water vapors alter dielectric permittivity and interfacial polarization, increasing capacitance in NAA	[96]
BOL removed Altering pore size and uniformity via anodization temperature (5 °C, 10 °C, and 20 °C) Au/NAA/Au multilayer: ring-shape Au electrodes deposited via sputtering	Ethanol (5–200 ppm)	Ethanol interacts with surface hydroxyl and oxalate groups, altering dielectric constant and capacitance of NAA	[97]
BOL removed Hydrophobization with 3-aminopropyltriethoxysilane (APTES) Au/NAA/Au multilayer: Au films deposited via sputtering	Ethanol (2.5–50 ppm)	Ethanol modifies effective dielectric constant inside NAA pores, shifting capacitance; silanization improves humidity resistance and hence boosts ethanol sensitivity	[98]
BOL removed In situ growth of CNT via acetone pyrolysis Au/CNT–NAA/Au multilayer: Au films deposited via sputtering	NH_3_ (1–15 ppm) HCOOH (35–175 ppm)	Gas adsorption on CNT arrays alters dielectric properties, modulating capacitance via physisorption-driven modulation of electronic parameters in the CNTs	[99]
CMOS-MEMS-compatible architecture integrated with microheater Au/NAA/Ti multilayer: Au electrodes deposited via electroplating	Humidity (0–100%RH)	Water monolayer adsorption (at low RH) and capillary condensation (at high RH) in NAA pores alter dielectric permittivity, and thus, capacitance	[103]
High-field anodization in phosphoric acid electrolyte Altering pore size and anion distribution via pore-widening treatment Au/NAA/Al multilayer	Humidity (5–95%RH)	Water molecules adsorb at anionic sites on pore walls, reducing surface potential barriers and increasing conductivity, via electronic conduction (at low RH) and proton conduction (at high RH)	[104]
Altering pore size and anion distribution via anodization voltage and pore-widening treatment Ag interdigital capacitive pattern deposited via e-beam evaporation	Humidity (10–90%RH)	Water adsorption in large AAO pores increases permittivity; with larger pores reduce humidity sensing threshold, higher anion concentration in the pore walls enhances water uptake and sensitivity	[106]
Two-step anodization with increasing oxalic acid concentration (0.3 M, 0.5 M, and 0.7 M) Pt/NAA/Al multilayer: Pt film was deposited via sputtering Magnetic-assisted sensing operation	Humidity (15–80%RH)	Phonon-assisted tunneling and dipole alignment boost response; anion-rich pores and magnetism enhance sensitivity	[107]
HPA with alternating voltage between 40 V and –2 V Pt/NAA/Al multilayer: Pt film was deposited via sputtering Magnetic-assisted sensing operation	Humidity (15–80%RH)	Adsorbed water vapors alter dielectric permittivity and increase capacitance of NAA; and magnetic field aligns water dipoles in NAA pores, increasing sensitivity and response linearity	[108]
Eco-friendly SMSA with SRB detachment NAA/Au: Au interdigital electrode deposited on BOL	Humidity (40–90%RH)	Water adsorption in AAO nanopores changes permittivity; interdigitated electrodes enhance capacitance response	[109]
One-step HPA of commercial 1050 Al alloy Pt/NAA/Al multilayer: Pt electrode was deposited via sputtering with photomask	Humidity (20–80%RH)	Shorter diffusion paths and larger adsorption area of thin AAO film with wide pores accelerates adsorption, enhancing capacitance and shortening response time	[110]
On-chip sensor design with: Grid-like Pd thin film deposited via sputtering and annealing Electroplated Au grid electrode via a Ti-Cu-Au multilayer structure patterned by photolithography	Humidity (-)	Water adsorption in porous alumina increases dielectric constant; CMOS-compatible sensor, with NAA film acts as a hygroscopic dielectric, while an ultra-thin palladium or gold grid electrode allows vapor permeation	[112]
Rapid anodization of NAA on paper substrate NAA/Al/paper: interdigitated Al electrode deposited via thermal evaporation	Humidity (20–80%RH)	Humidity detection relies on both dielectric constant change and surface conduction within the NAA; at low RH, phonon-induced electron tunneling dominates, while at high RH, protonic conduction dominates	[113]
GO functionalization using drop-casting of GO dispersion synthesis via Hummer’s method Au/GO–NAA/Al multilayer: Au permeable porous layer deposited via sputtering	Humidity (0–99%RH)	GO–NAA leveraging synergistic hygroscopic behavior enhances water adsorption and increases capacitance	[114]
Altering pore size via anodization voltage and acid electrolyte concentration Ag/NAA/Al multilayer: Ag electrodes deposited via manual screen printing	Humidity (3–98%RH) Moisture (3–800 ppm)	Moisture adsorption in AAO pores alters capacitance; pore morphology and wall anion distribution tailor sensitivity	[117]

*All sensors summarized in this table use straight-pore NAA structures unless otherwise stated

**Reported concentration ranges correspond to the tested working range of the sensors

Radzik et al. investigated a hybrid thick-thin film NAA-based sensor for methanol vapor detection, showcasing its capability to differentiate between water and methanol vapors. The NAA structure was fabricated by anodizing a 1-μm-thick aluminum thin film deposited on an alumina substrate, using 4% oxalic acid at room temperature under a constant voltage of 40 V. The resultant NAA structures featured nanopores with an average diameter of ~ 40 nm [95]. The sensor incorporated an interdigitated capacitor pattern screen-printed onto the NAA surface, with electrical connections established through a dual-pin configuration. Methanol exposure resulted in measurable changes in capacitance and resistance, with frequency-dependent responses analyzed using Cole–Cole plots. Changes in capacitance followed an exponential correlation with the dilution factor, with the highest sensitivity observed at frequencies below 2 kHz. Methanol adsorption within the nanopores significantly altered both dielectric properties and resistance, enabling the distinction between methanol and water vapor through distinct impedance behaviors. The observed resistance variations were uniquely characteristic of this sensor and differ from those typical standard capacitive humidity sensors.

Podgolin et al. developed capacitive sensors capable of distinguishing between different vapors by leveraging a through-hole NAA platform with enhanced selectivity achieved through electrochemical impedance spectroscopy (EIS) and field-induced dissociation effects [96]. The NAA structures were fabricated by anodization in 0.3 M oxalic acid at 40 V and 120 V, producing nanopores with diameters of 35 ± 5 nm and 100 ± 15 nm, respectively. The sensors featured an Au/NAA/Au sandwich structure with 20-nm-thick gold (Au) electrodes sputtered on both sides, and capacitance measurements were conducted over a frequency range of 1 Hz–10 kHz. The sensing mechanism relied on capacitance changes caused by vapor adsorption, polarization, and dissociation at the NAA-electrode interface. Using EIS, the researchers performed a detailed analysis of capacitance variations, enabling the discrimination of water, methanol, ethanol, and nonpolar gases such as isobutane and SF_6_, based on differences in dielectric constants, dipole moments, and polarizability. The sensors demonstrated sensitivity to relative humidity (RH) ranging from 10 to 85%, with capacitance increasing by over five orders of magnitude and response times varying between 26 and 122 s.

**Fig. 3:**
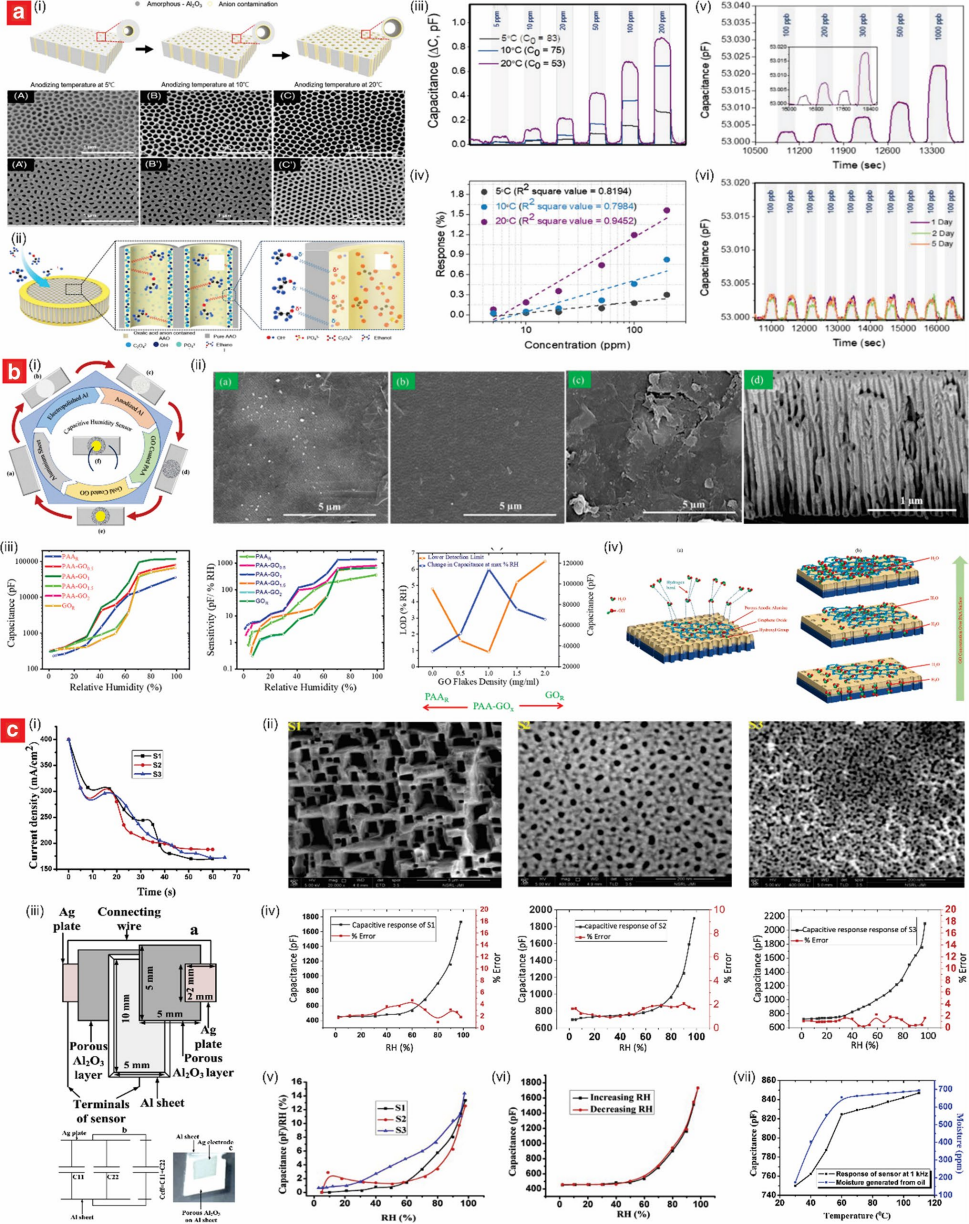
Engineering of NAA-based capacitive sensors. **a** NAA-based chemi-capacitive sensors for ppb-level detection of ethanol vapor at room temperature: (i) concept schematic and SEM images of NAA platform produced at various anodization temperature (i.e., 5, 10, 20 °C) in 0.3 M oxalic acid; (ii) sensing mechanism; (iii) response and (iv) sensitivity analysis with different ethanol gas concentrations; and (v) response and stability at ppb-level detection of ethanol (reproduced from ref. [97] with permission from The American Ceramic Society copyright 2023). **b** Engineering of a NAA–GO bilayer system by optimizing GO dispersion to achieve ultrafast humidity sensing: (i) fabrication process and (ii) SEM characterization of NAA coated with increasing GO thickness; (iii) transient response, sensitivity and LOD analysis at various levels of %RH; and (iv) sensing mechanism of NAA–GO sensors (reproduced from ref. [114] with permission from The Royal Society of Chemistry copyright 2023). **c** Online monitoring of moisture in transformer oil using parallel-plate NAA-based capacitive humidity sensors: (i) engineering of NAA-based sensors via variation of current density and (ii) SEM images of corresponding NAA platform; (iii) schematic of fabricated sensor and equivalent electrical circuit; (iv) capacitive response, (v) sensitivity analysis, and (vi) hysteresis curve of the fabricated sensors; and (vii) measurement of moisture in vapor generated through the transformer oil (reproduced from ref. [117] with permission from Elsevier copyright 2020)

Using a similar architecture, Park et al. developed capacitive ethanol sensors based on through-hole NAA films, achieving ppb-level detection with high selectivity, rapid response times, and the ability to operate reliably under varying humidity conditions (Fig. 3a) [97]. The sensors were fabricated via the two-step anodization process in 0.3 M oxalic acid. The first anodization was conducted at 50 V and 5–20 °C for 30 min, followed by chemical etching with 1.8 wt% chromic acid and 6 wt% phosphoric acid at 60 °C for 2 h to remove the irregular oxide layer. A second anodization at 55 V for 8 h produced straight nanopores with diameters of 65–99 nm, porosities of 29–50%, and thicknesses of 88–108 µm (Fig. 3a(i)). Au electrodes were sputtered onto the NAA surface, forming a ring-shaped electrode at the top and a circular electrode at the bottom. These electrodes were connected to an LCR meter for capacitance measurements at 1.0 V and 500 Hz. The sensing mechanism relied on interactions of ethanol molecules with hydroxyl and oxalate groups on the NAA surface, which altered the dielectric constant of NAA and led to capacitance changes (Fig. 3a(ii)). The sensor achieved a response of 1.56% to 200 ppm ethanol at 20 °C, with a limit of detection (LOD) of 16.49 ppb (Fig. 3a(iii–vi)). The response and recovery times were less than 30 s across all ethanol concentrations. Despite some sensitivity to moisture, the sensor retained 60% of its performance at 65%RH and exhibited good selectivity for ethanol, with minimal interference from toluene, sulfur dioxide (SO_2_), and carbon monoxide (CO).

Most recently, the potential of NAA for capacitive ethanol vapor detection was demonstrated using an Au/NAA/Au membrane structure [98]. The NAA substrate was a commercially sourced NAA membrane, featuring a straight pore structure with a pore size of 200 nm, thickness of 60 µm, and porosity of 30%. To create the sensor, Au electrodes were deposited using direct current (DC) sputtering in high vacuum, with the bottom electrode thickness fixed at 100 nm and the top electrode varying between 50 and 200 nm. Surface modification was achieved through silanization by immersing the sample in a 10 mM APTES toluene solution for a specified duration. The hydrophobically treated sensor demonstrated promising results for ethanol detection within the tested range of 2.5 to 50 ppm. At a frequency of 1 kHz, the sensor achieved responses ranging from 0.0075 to 0.068, indicating its capability for reliable detection across this concentration range. Furthermore, the sensor exhibited fast response and recovery times of approximately 27.54 and 26.26 s, respectively, at an ethanol concentration of 10 ppm. Additionally, functionalization with APTES significantly minimized moisture interference, reducing interferences from humidity by 34% and further enhancing selectivity towards ethanol detection under varying environmental conditions. The detection mechanism was based on changes in the dielectric constant of the NAA material upon ethanol adsorption. Ethanol molecules entering the nanopores displaced air, which altered the permittivity of the dielectric material and resulted in measurable changes in capacitance. Moreover, the hydrophobic surface treatment reduced interference from moisture while maintaining ethanol sensitivity with minimal degradation.

The nanostructure of NAA provides a versatile scaffold for integrating a broad range of active materials, enabling advanced gas sensors that exploit changes in electrical or dielectric properties for sensitive and selective detection. Chen et al. developed capacitive sensors for ammonia (NH_3_) and formic acid (HCOOH) detection, achieving rapid response and recovery times with enhanced sensitivity by utilizing vertically aligned carbon nanotube (CNT) arrays embedded in NAA templates [99]. NAA templates were fabricated via the two-step anodization process in 0.3 M oxalic acid at 40 V, followed by a BOL removal. Arrays of CNTs were subsequently grown within the NAA template through acetone pyrolysis at 750 °C for 60 min, yielding uniform CNTs approximately 40 nm in diameter. Au layers were sputtered onto the CNT/NAA arrays to create electrical contacts, and adhered to silicon substrates with silver conductive paste. The sensing mechanism relied on changes in the dielectric constant of the CNTs due to physisorption of gas molecules on their surface, which altered the capacitance of the sensor. These sensors achieved rapid response times of 100 s for 15 ppm NH_3_ and 20 s for 175 ppm HCOOH, with sensitivities of up to 10% and 15%, respectively, at room temperature.

Humidity sensing represents the most extensively explored application of NAA-based capacitive sensors, owing to the high surface area, tunable pore structure, and dielectric properties of NAA, which are well-suited for detecting changes in environmental moisture levels [100–102]. Juhasz et al. developed a capacitive humidity sensor for detecting RH, achieving exceptional sensitivity through optimized NAA morphology and integration with MEMS-compatible processes [103]. The NAA platform was fabricated via selective anodization of a 500-nm aluminum layer in 10% sulfuric acid under a constant current density of 8 mA⋅cm^–2^, and the electrodes were created from titanium (Ti) and Au via electrodeposition. The detection mechanism relied on changes in capacitance driven by water molecules adsorption and capillary condensation within the nanopores, with permittivity changes as water replaced air in the porous dielectric. The sensor demonstrated non-linear sensing characteristics, with significantly enhanced sensitivity of 15 pF/%RH at RH levels above 80%, attributed to capillary condensation effects. Yao et al. employed high-field anodization combined with isotropic chemical etching to fabricate NAA films. The sensors exhibited enhanced performance over a wide range of RH, from 5 to 95%, due to the optimized pore geometry and structural modifications achieved through controlled anodization and chemical etching processes [104]. The NAA films were fabricated via high-field anodization in a phosphoric acid electrolyte system at 195 V, yielding uniform hexagonal pores with an initial diameter of 180 nm. Subsequent isotropic chemical etching in phosphoric acid enlarged the pores and redistributed anion impurities, which played a critical role in enhancing sensitivity. The optimal performance was achieved with 30 min of etching, where increased surface area and enhanced anion concentration promoted efficient water molecule adsorption and diffusion through the nanopores. This improvement was evidenced by the remarkable dynamic response, with capacitance changes spanning 2–3 orders of magnitude across the tested humidity range. The sensitivity, defined as the ratio of capacitance at 85% RH to 35% RH (*C*_*85%RH*_*/C*_*35%RH*_), progressively increased with etching time, reaching a maximum of 348 at 40 min of etching. Additionally, the sensor’s rapid response and recovery times, approximately 24 s and less than 4 s respectively, were attributed to the large pore size and improved diffusion pathways provided by the isotropic etching process.

Advancements in the fabrication and structural optimization of NAA platforms have enabled substantial enhancements in the performance of NAA-based capacitive humidity sensors. These studies have demonstrated the critical role of tailored anodization conditions and innovative processing methods in improving sensitivity, stability, and response times [105]. Almasi Kashi et al. developed capacitive RH sensors for detecting RH (10–90%) with ultrafast response and recovery times, leveraging large-diameter nanopores and optimized anion concentrations to enhance sensitivity and reduce the RH detection threshold to 30% [106]. The NAA templates were fabricated via accelerated mild anodization in phosphoric acid at 165 V and 185 V, followed by post-anodization nanopore modification via pore widening process. The resultant NAA platforms featured pores diameters of 67 to 190 nm, with larger pores showing superior sensing performance. Silver (Ag) interdigital electrodes (~ 250 nm thick) were deposited via electron-beam evaporation, and electrical contacts were formed using Ag paste and copper wires, annealed at 90 °C for stability. The detection mechanism relied on impedance changes caused by water molecule adsorption and condensation within the pores. At lower RH levels, electronic conduction was the determining factor, while protonic conduction was dominant at higher humidity levels, facilitated by the enhanced adsorption on the anion-rich pore walls. The sensors demonstrated high sensitivity, with the maximum performance observed for pore diameters over 100 nm, response times of ≤ 5 s, and recovery times of ≤ 0.5 s, classifying them as ultrafast.

Chung and co-workers explored various anodization factors that affect the performance of NAA-based capacitive humidity sensors. They demonstrated that incorporating magnetic fields during a two-step anodization process in oxalic acid (0.3–0.7 M) at 40 V and 25 °C improved the response stability and linearity of the sensors, particularly at low RH levels [107]. The applied magnetic field promoted an orderly arrangement of water molecules within the NAA pores, enhancing the dielectric constant by facilitating bonding between physiosorbed and chemisorbed water layers. Platinum (Pt) thin films (10 nm) were sputtered onto the NAA surface to create electrical contacts. The sensors exhibited a rapid response time of 32 s and recovery time of 10 s at RH levels between 15 and 50%, demonstrating enhanced sensitivity due to phonon-assisted electron tunneling at low RH and increased pore conductance at higher RH levels.

Further advancing these concepts, other studies have been conducted to investigate a range of fabrication techniques and materials to enhance sensing performance. Chen et al. developed capacitive humidity sensors for detecting RH (15–80%), achieving improved sensitivity and linearity through the application of a magnetic field, which aligned water dipole moments and enhanced dielectric properties [108]. The NAA structures were fabricated using a two-step hybrid pulse anodization (HPA) process in 0.3 M oxalic acid, alternating 40 V for 1 s and − 2 V for 1 s to minimize Joule heat and improve pore uniformity. The resultant NAA film had an average pore diameter of 41.2 ± 2.7 nm and a thickness of 17.1 µm. A Pt thin film was sputtered onto the top side of the NAA layer, while aluminum served as the bottom electrode. At low RH (15–45%), chemisorbed and first-layer physisorbed water molecules minimally affected capacitance. At higher RH (45–80%), secondary water layers formed, with magnetic field application enhancing dipole moment alignment and clustering, increasing the dielectric constant. Under the external application of a magnetic field of 0.058 T, the response of the sensor reached 166.5% at 80% RH, compared to 79.7% without the action of the external magnetic field, demonstrating the effectiveness of the approach in improving performance across varying humidity levels.

Jeong et al. introduced scalable and eco-friendly fabrication approaches for NAA-based capacitive sensors, employing a simultaneous multi-surface anodization (SMSA) method in 0.3 M oxalic acid [109]. Anodization was conducted under mild anodization (MA) conditions at 15 °C and 40 V, resulting in nanopores with diameters of 35 ± 5 nm or 100 ± 15 nm, depending on the conditions. The flow-through design, achieved by removing the BOL and the remaining aluminum from the backside of the as-produced NAA platform, allowed for enhanced gas permeation and reduced resistance. The sensor structure incorporated interdigitated Au electrodes placed on either the BOL or open-pore side of the membrane. The sensing mechanism was based on capacitance changes induced by the adsorption and polarization of vapor molecules within the nanopores. Water molecule chemisorption and physisorption were particularly influential, with the BOL contributing to a linear response without additional modifications. These sensors demonstrated a linear capacitance response to RH ranges of 40–70% at 100 kHz and 60–90% at 1 MHz, with response and recovery times ranging from 26 to 122 s, depending on the AC frequency. In addition to humidity sensing, the sensor exhibited sensitivity to other gases, including SF_6_, carbon dioxide (CO_2_), nitrogen (N_2_), and helium (He), with responses varying according to polarizability and molar mass. This versatility underscores the applicability of the NAA-based platform for both polar and nonpolar gas detection.

Furthermore, a one-step anodization process on commercial 1050 aluminum alloy was developed, utilizing hybrid-pulse anodization in 0.3 M oxalic acid at 20 V and 25 °C [110]. This cost-effective and scalable method produced thinner NAA layers with increased pore density, leading to shorter diffusion paths for water molecules. Pt electrodes were sputtered with a photomask to define specific areas for electrical contact. The sensors exhibited a remarkable capacitive response of 5013% in measured capacitance change at 80% RH, with a response time of 9 s and recovery time of 9 s. The sensing mechanism involved rapid adsorption and desorption of water molecules on the nanopore walls of NAA, enhanced by the large surface area and reduced thickness of the NAA layer.

Integrating novel substrates and hybrid materials has shown potential for enhancing the performance and versatility of capacitive NAA-based humidity sensors [111]. Juhász and Mizsei developed capacitive humidity sensors using thin-film NAA layers integrated with CMOS-compatible processing technology [112]. The NAA structures were fabricated by anodizing aluminum thin films deposited on SiO_2_ layers, using either 20 wt% sulfuric acid at 10 mA⋅cm^–2^ or 9.6 wt% sulfuric acid at 8 mA⋅cm^–2^. These processes produced nanopores with diameters of approximately 8 and 18 nm, respectively. Two sensor configurations were studied: the first employed an annealed, ultra-thin Pd electrode forming a grid-like structure, while the second featured an electroplated Au grid. Contacts were created using thermocompression bonding with 50-µm Au wires. The sensing mechanism relied on capacitance changes induced by water adsorption within the NAA pores, altering the dielectric properties of the material. The first sensor exhibited a sensitivity of 4–5 pF/%RH, while the second achieved a three-fold higher sensitivity of 15 pF/%RH. This enhancement was attributed to the increased porosity and structural enhancements. Response times ranged from 60 to 90 s, with both designs surpassing the performance of commercial capacitive sensors.

Balde et al. developed a flexible capacitive humidity sensor by integrating porous NAA layers on a paper-based substrate, enabling detection of RH in the range 20–80%, with enhanced sensitivity and a lightweight design [113]. The NAA layers were fabricated using mild anodization in 15 wt% phosphoric acid at 100 and 140 V for 100 s. These processes produced NAA films with pore diameters of 40–90 nm and 40–150 nm, thicknesses of 720 nm and 1300 nm for the NAA structures produced at 100 and 140 V, respectively. It was found that higher anodization voltages resulted in NAA structures with improved circularity and uniformity. The NAA layer served as the active sensing material, with interdigitated electrodes formed by evaporating 300 nm of aluminum onto the NAA surface using a Pt mask. The detection mechanism relied on variations in capacitance associated with water molecules adsorption within the nanopores. At low RH (< 50%), chemisorbed water molecules formed hydroxyl groups, resulting in minor capacitance changes governed by electronic conduction. At higher RH (> 50%), physisorbed water layers facilitated proton conduction through a continuous hydrogen-bonded network of water molecules, leading to capacitance increases that were three times higher at 1 kHz compared to 100 kHz, and up to twice as high for NAA layers fabricated at 140 V compared to those at 100 V. The sensor exhibited enhanced sensitivity, particularly at lower frequencies, with a sensitivity of 0.652 pF/%RH measured at 70% RH.

Alam and Islam developed a capacitive humidity sensor using a graphene oxide (GO) and NAA bilayer system, demonstrating exceptional sensitivity and an ultra-low detection limit (Fig. 3b) [114]. The NAA layer was fabricated via anodization in 0.3 M sulfuric acid at 25 V for 1 h, resulting in pores with an average diameter of ~ 25 nm and a depth of ~ 1 µm. A GO layer was synthesized using a modified Hummers’ method and deposited onto the NAA surface through drop-casting, achieving an optimized NAA–GO configuration with a 1.0 mg/mL GO dispersion (Fig. 3b(i–ii)). The sensor featured a capacitive design with NAA as the base electrode and a sputtered Au layer on the GO surface as the top electrode. The NAA–GO sensor exhibited a high responsiveness to humidity across the range of 0.9–99% RH and a sensitivity of 1178.76 pF/%RH, with a rapid response time of 0.8 s and a negligible hysteresis of 0.129% (Fig. 3b(iii)). The sensing mechanism relied on the synergistic interplay between the GO and NAA layers. At low RH, water molecules penetrated the GO layer to interact with the NAA surface, enhancing the capacitive response. At higher RH, multilayer adsorption on GO flakes, facilitated by hydrophilic functional groups, dominated the signal response upon exposure (Fig. 3b(iv)). This combination enabled the sensor to achieve an ultralow LOD of 0.9% RH, which was attributed to the optimized GO dispersion.

Capacitive gas sensors based on NAA platforms have shown adaptability and resilience, making them effective in challenging environments where conventional sensors may falter due to harsh conditions [115, 116]. Kumar et al. developed a capacitive humidity sensor capable of detecting water vapor (3–98% RH) and moisture in transformer oil (180–800 ppm), with enhanced sensitivity attributed to optimized nanopore morphology and stable Ag electrodes (Fig. 3c) [117]. The NAA platform was fabricated by anodization in oxalic acid under varying conditions (i.e., 40–60 V and 4–15.5 mA⋅cm^–2^) to produce nanopores with diameters ranging from 10 to 262 nm (Fig. 3c(i–ii)). Ag electrodes were applied via screen printing and annealed for stability (Fig. 3c(iii)). Sensitivity optimization was achieved through tailored pore morphology, with the smallest pores (i.e., 10 nm) enabling a linear capacitive sensitivity across the full humidity range (Fig. 3c(iv–v)). The sensor demonstrated excellent stability, with minimal hysteresis error (~ 3.5%), rapid response, and recovery times (27 and 40 s, respectively), and robust performance during prolonged exposure to contaminated transformer oil (Fig. 3c(vi–vii)).

### Catalytic sensors

Catalytic gas sensors have long been a cornerstone in the detection of flammable gases due to their reliability, robustness, and ability to operate at high temperatures, humid environments, and areas with varying gas concentrations. These sensors operate on the principle of flameless combustion, where target gases react on heated catalytic elements, generating heat that is proportional to the gas concentration. This design is highly effective in detecting combustible gases, particularly in environments where factors such as humidity or interfering background gases could compromise the performance of other sensor types. A typical catalytic sensor consists of a sensing element coated with a catalytic material, a heating source to maintain the element at a suitable temperature, and a temperature-sensitive transducer that measures the heat generated during the reaction. Among the various platforms for developing catalytic gas sensors, NAA has emerged as a highly effective substrate, distinguished by its unique nanoporous structure, which provides high surface area, excellent thermal stability, and seamless integration with catalytic materials. To date, NAA platforms have been used to develop catalytic gas sensors designed to detect a variety of gases (Table 2), with flammable gases being the most common targets [118, 119]. In addition to its intrinsic properties, NAA-based catalytic sensors can also be integrated into MEMS platforms. These designs serve as a foundation for addressing the growing demand for robust and energy-efficient gas detection technologies, providing a basis for more specialist applications in gas sensing.

**Table 2:** Summary of NAA-based catalytic sensors

Sensor design/chemical modification*	Detected gases**	Sensing mechanism	Ref
BOL removed Pd–Pt/NAA-based planar microheater-integrated catalytic sensor Pd and Pt catalytic noble metal clusters functionalization	CH_4_ (0.17–2.45 vol.% in air)	Step-heated Pt–Pd catalyst enables methane detection via catalytic combustion, minimizing humidity interference	[119]
BOL removed Pd–Pt/NAA-based planar microheater-integrated catalytic sensor Pd–Pt bimetallic catalytic nanoparticle functionalization Hysteresis behavior analysis under operando operation	CH_4_ (2.56 vol.%)	Methane detection via catalytic combustion on Pt–Pd catalyst; a Wheatstone bridge with catalyst and non-catalyst micro-hotplates isolates the combustion signal	[121]
BOL removed Pd–Pt/NAA-based planar microheater-integrated catalytic sensor Pulsed operation mode	H_2_ (0.1 to 2 vol.% in air)	H_2_ oxidizes on Pd–Pt catalyst, generating heat that raises microheater resistance, detected by Wheatstone bridge circuit, under pulsed heating mode	[122]
Functionalization with Pt nanoparticles synthesized using a biological extract from *Asparagus racemosus Linn* via chemical bath deposition	CO_2_, methanol, ethanol, acetone (1,000 ppm)	Reducing gases react with adsorbed oxygen species (O⁻, O_2_⁻) on Pt-nanoparticles-coated porous alumina, lowering resistance via electron release	[124]

*All sensors summarized in this table use straight-pore NAA structures unless otherwise stated

**Reported concentration ranges correspond to the tested working range of the sensors

**Fig. 4:**
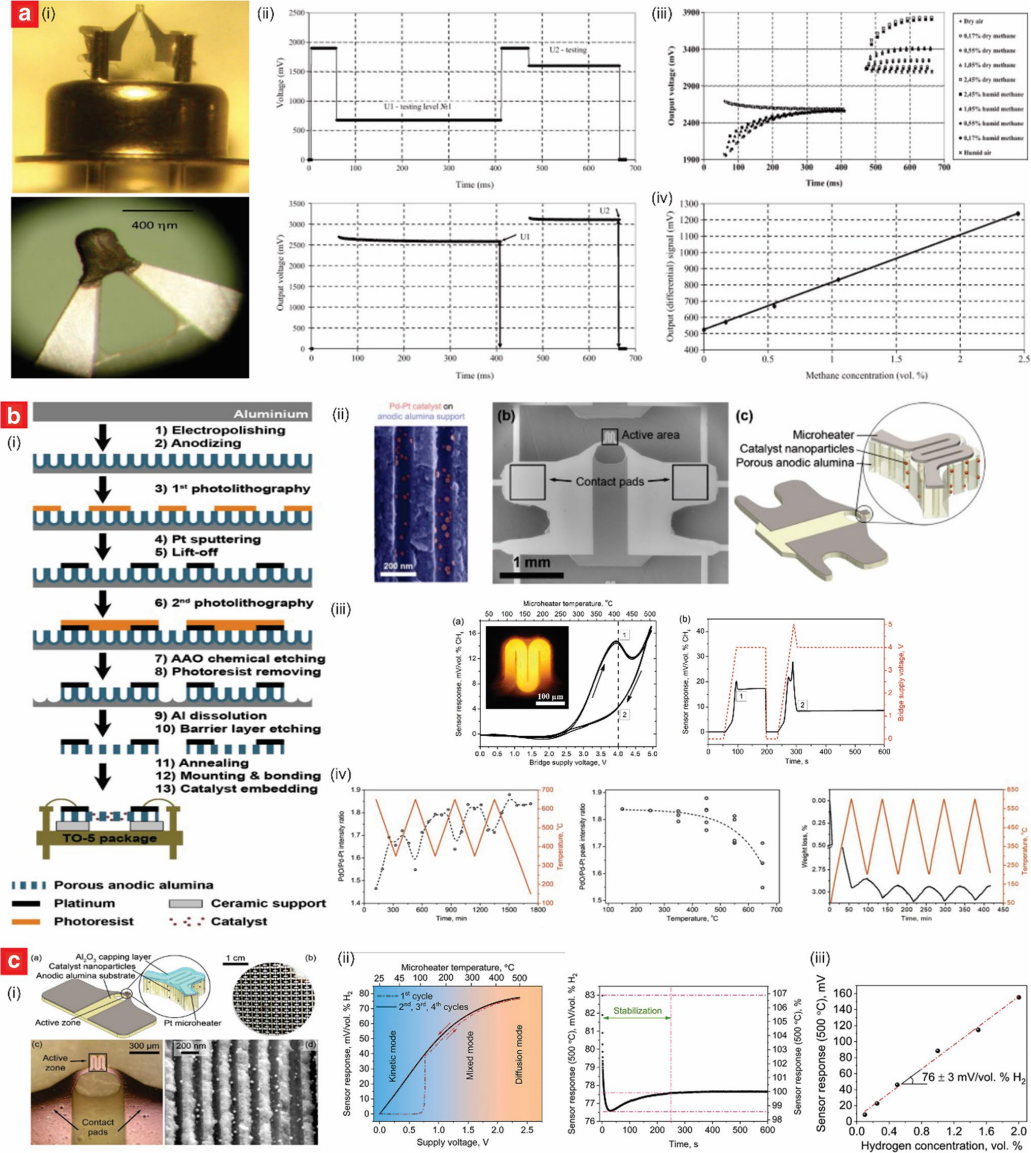
Engineering of NAA-based catalytic sensors. **a** Engineering of an energy-efficient planar NAA-based catalytic sensor for CH_4_ detection with minimized humidity interference: (i) digital images of the sensor design; (ii) sensor response to CH_4_ under pulsed-voltage operation; (iii) sensor response at different concentrations of methane in dry and humid air; and (iv) sensitivity analysis. (reproduced from ref. [119] with permission from Elsevier copyright 2013). **b** Engineering of Pt/NAA-based micro-hotplate catalytic sensors to study CH_4_ detection and response hysteresis under *operando* conditions: (i) fabrication process of Pd–Pt/NAA sensor in MEMS-compatible package; (ii) SEM images and schematic of the fabricated sensor; (iii) hysteresis in the response of catalytic sensor and time-stability analysis of the sensor response at constant bridge supply voltage in forward and reverse sweep; and (iv) the evolution of the catalyst phase composition was studied under *operando* conditions in dry air with 2.56 vol. % of CH_4_ (reproduced from ref. [121] with permission from Elsevier copyright 2020). **c** Engineering of low power consumption microheater-based catalytic sensors on NAA for H_2_ detection at high temperatures: (i) schematic and SEM images of the active element; (ii) Sensor response of the developed catalytic hydrogen sensor for 2 vol% H_2_ in air exposure, and the dependence of the sensor response on time; and (iii) sensor response to various H_2_ concentrations at operating temperature of 500 °C (reproduced from ref. [122] with permission from Elsevier copyright 2024)

Recent advancements have demonstrated the potential of a sensor design based on NAA substrate functionalized with catalytic metal (e.g., Pt and Pd). This combination enabled enhanced combustion reactions and improved sensor performance for methane (CH_4_) and hydrogen (H_2_)—two key target flammable gases [119–123]. Planar CH_4_ sensor designs utilizing Pt and Pd catalysts on NAA substrates have shown exceptional and reliable performance, and energy-efficient operation that supports deployment in stand-alone wireless sensor networks [119–121]. Karpov et al. presented an energy-efficient planar catalytic sensor for CH_4_ detection, distinguished by its innovative use of a differential measurement method and a free wedge-shaped alumina membrane to enhance stability and minimize energy consumption (Fig. 4a) [119]. The sensor was based on NAA membranes fabricated through the anodization of aluminum foil shaped into a free wedge configuration to prevent bending from thermal expansion. NAA membranes were functionalized with Pt and Pd individually, using an impregnation process followed by annealing at 500 °C to form noble metal clusters. A micro-heater was integrated via lithographic patterning and magnetron sputtering of Pt; a thin alumina passivation layer was coated on top to ensure durability and prevent the system from degradation. The detection mechanism relied on the catalytic combustion of CH_4_ on adsorption sites of the catalytic metals, and a differential method was employed to measure sensor responses at two distinct temperatures, 200 °C and 450 °C. As CH_4_ combustion occurs only at higher temperatures (i.e., 450 °C in this case), this approach effectively isolated the CH_4_ signal by subtracting the response to environmental factors such as humidity and temperature observed at 200 °C. The sensor exhibited a linear response across the tested CH_4_ concentration range, from 0.17 to 2.45 vol.%, with a sensitivity of 290 mV per % CH_4_ concentration at an amplifier gain of 20. The pulsed voltage operation mode employed demonstrated to be key to reducing energy consumption significantly, achieving an average power consumption of just 1.2 mW when measurements were taken twice per minute, enabling over a year of operation on three AA batteries.

Roslyakov et al. presented a micro-hotplate catalytic sensor for CH_4_ detection integrating NAA and bi-metallic Pd–Pt nanoparticles. This combination enhanced catalytic efficiency, stability, and response performance through synergistic effects, and mitigation of environmental interferences and response hysteresis (Fig. 4b) [121]. The NAA substrate was fabricated via a hard anodization process in 0.3 M oxalic acid electrolyte at 1 °C, where the anodization voltage was increased linearly to 120 V at a rate of 0.5 V/s. Upon reaching 120 V, the process was terminated when the electrical charge density (i.e., integration of current density through time) flowing through the system was 60 C⋅cm^–2^. The resulting porous structure had a thickness of 30 µm, an interpore distance of 256 ± 24 nm, and a pore diameter of 150 ± 11 nm, providing a robust template for catalyst deposition. The functionalization of the resultant NAA templates involved embedding bi-metallic Pd–Pt nanoparticles, with an average diameter of 21 ± 8 nm, into the porous channels via soaking of the NAA platform in a solution of 0.6 M PdCl_2_ and 0.2 M H_2_PtCl_6_, followed by thermal decomposition at 500 °C and aging at 400 °C for 48 h. Electrical contacts were integrated using a 300-nm platinum micro-heater deposited via magnetron sputtering and structured through lift-off photolithography. The underlying aluminum foil and BOL at the bottom of the nanopore tips were chemically etched to produce free-standing micro-hotplates, which were mounted on TO-5 packages with 3D-printed ceramic supports and Au wire bonding for electrical connections. The sensor demonstrated effective CH_4_ detection, with a maximum sensitivity of 15 mV per vol.% CH_4_ at a supply voltage of 3.9 V, corresponding to an active area temperature of approximately 400 °C. CH_4_ oxidation initiated at ~ 270 °C, and self-ignition was observed above 500 °C. The detection mechanism of the sensor was based on the catalytic oxidation of CH_4_ on bi-metallic Pd–Pt nanoparticles, where cyclic oxidation and reduction of palladium oxides (PdOx) during heating and cooling influenced the active site availability and drive the response of the sensor. A unique feature of the sensor was its response hysteresis during forward and reverse voltage sweeps, attributed to changes in the PdOx/Pd ratio on the catalyst surface. Operando studies using XRD and thermogravimetry confirmed that the hysteresis resulted from phase transitions between Pd and PdOx, which influenced the catalytic activity for CH_4_ oxidation.

Similarly, Kalinin et al. developed a high-performance microheater-based catalytic H_2_ sensor based on the design of bi-metallic Pd–Pt nanoparticles embedded in a NAA substrate (Fig. 4c) [122]. The NAA substrate was fabricated through anodization of aluminum foils in 0.3 M oxalic acid at a constant voltage of 100 V, producing a structure with open porosity and a high specific surface area enhanced through annealing. The functionalization process involved the deposition of Pd–Pt bi-metallic nanoparticles onto the nanopore walls via incipient wetness impregnation, followed by thermal decomposition at 500 °C. Pt micro-heaters were fabricated on the NAA substrate via a combination of magnetron sputtering, photolithography, and ion beam etching techniques, while electrical connections were established with Au wire bonding to ensure robust contact. The sensor exhibited outstanding performance, demonstrating a high sensitivity of 76 mV/vol.% H_2_ and a LOD as low as 12 ppm. Its rapid response time of 0.4 s and excellent long-term stability, with a response deviation of less than 4% over 14 days, highlighted its reliability for practical applications. Furthermore, the sensor operated effectively without interference from humidity, even in conditions of high RH, due to the diffusion-controlled operation mode at high temperatures of 500 °C. The sensing mechanism relied on the catalytic combustion of H_2_ on the Pd–Pt nanoparticles, which generated heat and increased the resistance of the Pt micro-heater, allowing accurate determination of H_2_ concentrations using a Wheatstone bridge circuit.

Besides the main application in flammable gases detection, NAA-based catalytic sensors have also been developed for detecting VOCs such as methanol and ethanol. Nadekar et al. presented a VOC sensor based on Pt-embedded NAA films specifically designed to detect methanol, ethanol, acetone, and CO_2_. The novel integration of biologically synthesized Pt nanoparticles with a NAA structure enabled enhanced sensitivity at low-temperature operation—addressing key challenges in energy efficiency and reliability [124]. The NAA films were fabricated through anodization of aluminum substrates in a 40 wt% H_2_SO_4_ solution at a current density of 50 mA⋅cm^–2^ for 60 s, producing uniform pores with diameter of 20–50 nm. Sustainable and cost-effective functionalization of the NAA films was achieved by coating them with Pt nanoparticles synthesized from a biological extract of *Asparagus racemosus Linn*, using a chemical bath deposition method. The gas sensing mechanism relied on surface reactions involving adsorbed oxygen species, such as O₂⁻, O^2^⁻, and O⁻, on the Pt-embedded NAA surface. When exposed to reducing gases such as methanol, ethanol, acetone, or CO_2_, these oxygen species reacted with the gas molecules, resulting in changes to the resistance of the sensor. The sensor demonstrated outstanding performance for various VOCs, with a sensitivity factor of approximately 5.5 × 10^5^ for methanol and 4.5 × 10^5^ for ethanol at 40 °C, 950 for acetone at 40 °C, and 250 for CO_2_ at 80 °C. It also exhibited exceptional repeatability and reproducibility, with a response deviation error of ± 20. Moreover, its ability to operate at low temperatures (i.e., 40–80 °C) provided energy efficiency, making it suitable for practical applications.

### Chemiresistive sensors

NAA has emerged as an exceptional platform for the development of chemiresistive sensors due to its unique structural and material properties, which have enabled the realization of a broad variety of gas sensors [125, 126]. NAA exhibits tunable pore architectures with large surface areas, all of which are ideal for facilitating gas adsorption and promoting effective interaction with functional materials. Further to that, its inherent chemical and thermal stability ensure reliable operation in harsh environments. NAA-based chemiresistive sensors operate on the principle of detecting variations in electrochemical signals upon exposure to target gases. These changes are derived from redox reaction between the target gas molecules and the NAA substrate, which in turn modulate the electrical conductivity or resistance of the composite platform. The simplicity, cost-effectiveness, and ability to operate at either room or elevated temperatures make chemiresistive sensors a preferred choice for developing NAA-based gas sensors. NAA structures have been used in their bare form, such as for detecting NH_3_ and alcohol vapors through their physisorption properties. However, functionalization has proven an essential step for enhancing performances [125, 127]. Building on this foundation, functionalized NAA structures offer excellent customization with diverse active sensing materials (e.g., catalytic metals and metal oxides, conducting polymer, carbon-based materials, ceramic) to enhance gas interaction and improve overall sensing performance [128–137]. Table 3 summarizes the most representatives studies on the development of this class of sensor.

**Table 3:** Summary of NAA-based electrochemical sensors

Sensor design/chemical modification*	Detected gases**	Sensing mechanism	Ref
Catalytic metals			
High uniformity single-phase Au nanorods synthesized via NAA-assisted pulse electrochemical deposition	H_2_S (1–4 ppm) CH_3_SH (0.5–4 ppm)	S-containing gas molecules forming Au–sulfide bonds on surface of gold nanorods alters the charge distribution and increases resistance	[128] [129]
Ultra-thin Pt film deposited via sputtering	H_2_ (10–50,000 ppm)	H_2_ molecules dissociate on nanoporous Pt film, diffusing into the lattice and altering resistance via surface scattering	[138]
Pd film deposited via ALD with variable-timing cycles	H_2_ (10 vol.% in N_2_)	H_2_ adsorbs and diffuses into Pd film and forms PdHx, which disturbs electron flow and increases resistance	[126]
Pd film deposited via sputtering MEMS-platform and Pd microheater integrated via photolithography and thin-film deposition	H_2_ (25–5000 ppm)	H_2_ adsorbs and diffuses into Pd film and forms PdHx, which disturbs electron flow and increases resistance	[146]
Hydrophobic treatment with ODTMS Pd-capped Mg bimetallic ultra-thin films deposited via sequential sputtering of Mg and Pd	H_2_ (1–40,000 ppm)	H_2_ dissociates on the Pd cap and diffuses into Mg, causing a decrease in resistance due to MgH_2_-induced lattice expansion and altered electron transport	[140]
Bristle-like morphology Pd/Bi bimetallic nanowires deposited via co-electrodeposition MEMS integrated with interdigitated electrodes	H_2_ (1000–30,000 ppm)	H_2_ dissociates and forms PdHx, increasing resistance due to electron scattering and Bi-stabilized lattice expansion	[141]
Metal oxides			
Pd/WO_3-x_/NAA multilayer Oxygen-deficient WO_3-x_ with controlled oxygen vacancies deposited via sputtering Pd thin film deposited on WO_3-x_ via sputtering	H_2_ (50 ppb–30,000 ppm)	H_2_ dissociates on Pd and enters WO_3-x_, altering resistance due to oxygen vacancy-enhanced electron transfer	[131]
High-temperature sensor using rutile-phase TiO_2_ fabricated via e-beam evaporation of Ti and oxidation by sintering Pt interdigitated electrodes fabricated via photolithography	H_2_ (5–500 ppm)	H_2_ interacts with surface-adsorbed oxygen on rutile TiO_2_ thin films, reducing Ti^4+^ to Ti^3+^ and increasing electron density, which decreases resistance	[151]
High-temperature sensor using macroporous TiO_2_ thin film deposited via sputtering CMOS and MEMS compatible	O_2_ (500–6000 ppm)	O_2_ adsorption on macroporous TiO_2_ nanofilm extracts electrons, increasing resistance	[132]
Room-temperature gas sensor based on Mn_3_O_4_ nanorods deposited via vacuum infiltration Mn_3_O_4_ nanorods were synthesized via hydrothermal reaction Au/Mn_3_O_4_–NAA/Au multilayer: Au contacts deposited via sputtering	Ethanol, acetone (25–50 ppm)	VOCs adsorb on Mn_3_O_4_ nanorods, interacting with surface oxygen species and triggering charge transfer that alters resistance	[133]
Room-temperature NH_3_ gas sensor using highly ordered ZnO nanowire arrays fabricated via a vacuum suction method on NAA templates	NH_3_ (10–150 ppm)	NH_3_ molecules donate electrons to ZnO nanowires, reducing resistance via surface chemisorption	[134]
Room-temperature CO_2_ sensor using sub-20 nm SnO nanowires SnO nanowires deposited via electrodeposition Pt contact fabricated via electrodeposition	CO_2_ (5000 ppm)	Gas adsorption alters hole concentration in SnO nanowires, changing conductivity via surface charge transfer	[153]
Highly sensitive H_2_ gas sensor based on a porous network of ultrathin SnO_2_ nanowires synthesized via a drop-coating technique on NAA template, with Pd functionalization	H_2_ (0.5–200 ppm)	H_2_ molecules reduce adsorbed oxygen species (O⁻, O_2_⁻) on SnO_2_ nanowires, releasing electrons into the conduction band and decreasing resistance, with Pd doping catalyzes H_2_ dissociation	[135]
Fe-NiO_x_ nanotubes assembled using NAA templates, featuring Fe^3^⁺ doping to enhance oxygen vacancies Fe-NiO_x_ nanotubes synthesized via calcination of FeNi layered double hydroxides	H_2_S (50–1000 ppb)	H_2_S adsorbs on Fe–NiOx, altering resistance via Fe^3+^-facilitated chemisorption and electron transfer	[136]
H_2_ and CO gas sensor based on Sn_x_Bi_k_Mo_y_O_z_ composite deposited via ionic layering on NAA	H_2_ (5–50 ppm) CO (1–100 ppm)	Gases modulate charge at Sn_x_Bi_k_Mo_y_O_z_ composite layer, altering resistance through changes in surface charge and chemisorbed oxygen	[155]
Integration of a p-n heterojunction (SnO_2_/CuO) Hydrophobic SnO_2_/CuO bilayer thin film fabricated via pulsed laser deposition Pt contact deposited via sputtering	CO (5–500 ppm)	CO molecules reduce adsorbed oxygen species on SnO_2_/CuO heterojunction, injecting electrons and lowering resistance; while hydrophobicity improves gas selectivity and suppresses humidity interference	[154]
Room-temperature gas sensor SnO_2_ nanotubes deposited on NAA via ALD and decorated with noble metals (Pt, Pd, Au, Ag)	H_2_ (5–8000 ppm) Formaldehyde (50–1000 ppb) Toluene (50–1000 ppb) NO_2_ (100 ppb–20 ppm)	Gases interact selectively with metal-decorated SnO_2_ nanotubes, changing resistance via surface reactions and electron transfer	[156]
Carbon-based materials			
Room-temperature sensor using aligned CNT grown in NAA platform CNTs were grown via CVD Pd/CNT–NAA/Al multilayer: Pd and Al contacts were fabricated via sputtering and thermal evaporation	H_2_ (50 ppm–1.5%)	H_2_ forms PdHx on Pd-CNT/NAA, increasing resistance via lattice expansion and scattering at CNT interface	[157]
Room-temperature H_2_ gas sensor using CNT grown in anodic NAA via CVD with deposited nanoporous palladium electrodes	H_2_ (0.1–1.5%)	NH_3_ donates electrons to defect-rich CNTs in NAA, decreasing resistance via surface adsorption	[158]
Room-temperature sensor based on catalyst-free MWCNTs embedded in NAA templates MWCNTs grown via CVD	NH_3_ (100 ppm) NO_2_ (-)	NH_3_ donates electrons to MWCNTs in NAA, lowering resistance; plasma oxidation of this layer can tune current pathways, optimizing the resistive sensing response	[159]
NO_2_ and NH_3_ gas detection by using graphene film transferred onto the NAA via PMMA-assisted process	NO_2_ (1000 ppm in N₂) NH_3_ (1% in an Ar)	NH_3_/NO_2_ adsorb on graphene and alter the resistance through local charge modulation; NAA pores induce an inhomogeneous electrostatic potential, promoting charge redistribution and thereby enhancing sensitivity	[137]
Semiconducting polymers			
Stripe structure PEDOT/PSS nanowires with Au on both ends PEDOT/PSS deposited via electrochemical polymerization Au segments deposited via galvanostatic deposition	Methanol, ethanol, acetone (10–50%P/P_0_)	VOC vapors induce polymer swelling or charge depletion in PEDOT:PSS nanowires, increasing resistance via adsorption​	[162]
Highly sensitive ammonia sensor by P3HT fabricated using laser-patterning NAA	NH_3_ (100 ppb–100 ppm)	NH_3_ molecules interact with the p-type P3HT layer through charge–dipole interactions, donating electrons and reducing hole mobility, which decreases current	[163]
Other functional materials			
Pd-decorated SiC nanocauliflowers grown on Ag-coated NAA substrates SiC nanocauliflowers grown by co-sputtering of Si and C Pd thin film deposited on SiC via sputtering	H_2_ (2–500 ppm)	H_2_ dissociates on Pd and reacts with SiC surface oxygen, increasing conductivity via vacancy-driven electron release	[164]
Ethanol vapor sensor using Ti_3_C_2_T_x_ MXene films deposited on NAA membranes via vacuum filtration	Ethanol (0.1%)	Ethanol intercalates and swells Ti_3_C_2_T_x_ layers, altering resistance due to Na^+^-tuned interlayer transport​	[165]
CdSe nanorods capped with L-cysteine nanorods were fabricated using a scalable chemical bath deposition, followed by annealing	Ethanol (25–300 ppm)	Ethanol adsorption increases CdSe conductance; visible light enhances hole generation and surface interaction on NAA	[166]
Innovative structures and integrated platforms			
Ethanol and H_2_ gas sensor by Nb_2_O_5_ nanocolumns growing on NAA by re-anodizing	Ethanol (50–500 ppm) H_2_ (100–1000 ppm)	H₂/ethanol adsorb on Nb_2_O_5_ columns, modulating conductivity via surface reactions and Schottky barrier effects	[169]
MEMS- and microheater-integrated based H_2_ sensor utilizing nanostructured WO_3_ on a low-aspect-ratio NAA template WO_3_ deposited via sputtering and annealing Pt electrode deposited on top of via sputtering and photolithography	H_2_ (5–1000 ppm)	Hydrogen reacts with oxygen species adsorbed on nanostructured WO_3_, injecting electrons and lowering resistance	[170]
Gas sensor array using WO_3_ films prepared via sputtering over NAA	H_2_ (10, 100, and 1000 ppmv) CO (5, 10, and 50 ppmv) Ethanol (1, 5, and 10 ppmv) DMMP (1 and 5 ppmv)	Gases interact with WO₃ nanotubes via diffusion and adsorption; temperature modulation enhances selectivity and discrimination via gas-specific kinetic signatures	[171]
Microheater-integrated gas sensor based on NAA-supported SnO_2_-Pd nanostructures Dual-mode operation: continuous and pulse heating modes SnO_2_ thin film and Pd nanoparticles deposited via ALD and annealing	H_2_, acetone, toluene (4 ppt–1 ppm) HCHO (4 ppb–1 ppm)	Gases react on SnO_2_-Pd nanotubes, altering conductance via redox reaction; pulse heating extracts transient features for gas discrimination	[172]
H_2_ gas sensor by Nb_2_O_5_ nanorods growing on NAA by re-anodizing	H_2_ (100–5000 ppm)	H₂ reduces Nb_2_O_5_ nanorod surface, decreasing depletion width and increasing conductivity; conductive Nb base and Schottky barrier at the metal–oxide interface improves carrier extraction and gas sensitivity	[175]
Low-power and thermally robust microheater-integrated NO_2_ sensor Nanostructured WO_3_ and In_2_O_3_ layers deposited via glancing angle sputtering Ta/Pt heater deposited via sputtering Cr/Au interdigitated electrodes patterned via e-beam evaporation	NO_2_ (0.5–5 ppm)	NO_2_ induces redox reaction on SMO, altering resistance	[176]

*All sensors summarized in this table use straight-pore NAA structures unless otherwise stated

**Reported concentration ranges correspond to the tested working range of the sensors

#### Noble metals

Metal functionalization of straight-pore NAA sensing platforms has focused on catalytic metals such as Au [128, 129], Pt [138], and Pd [139–144]. Au nanorods, with their exceptional chemical affinity for sulfur (S)-containing gases and their ability to induce significant charge transfer and conductivity changes upon gas adsorption, provide a highly effective approach to enhancing the sensitivity and selectivity of gas detection, particularly for S-containing toxic analytes. For example, Li et al. presented a comprehensive investigation into the development of a highly sensitive hydrogen sulfide (H_2_S) gas sensor using Au nanorods synthesized within a NAA template [128]. A one-step anodization process (i.e., 0.3 M oxalic acid at 40 V and 7 °C) was used to fabricate the NAA templates, which featured a pore diameter of 70 nm (expanded by pore widening through wet chemical etching in phosphoric acid) and a pore length of 27 µm. The sensor’s active material, Au nanorods, was deposited into the NAA pores through pulse electrodeposition in an electrolyte containing HAuCl_4_·3H_2_O and HClO_4_. Electrical contacts were established using tungsten (W) needles attached to the nanorod array surface, and the sensing device was integrated into a custom-designed chamber for H_2_S detection. The sensor exhibited outstanding performance characteristics, with a sensitivity defined by a resistance change ratio (*ΔR/R*_*0*_) that increased up to 19 times upon exposure to H_2_S. The device demonstrated an LOD as low as 0.1 ppm and a rapid response time, which decreased from 450 to 30 s as the H_2_S concentration was increased from 1 to 4 ppm, respectively. These attributes underscore the applicability of the sensor for detecting trace levels of toxic gases with high temporal resolution. Additionally, the sensor showcased excellent selectivity, responding 15 times more strongly to H_2_S than to N_2_, and 50 times more sensitive than to argon (Ar). The underlying sensing mechanism involved the high electron affinity between H_2_S molecules and Au, leading to the formation of Au–S bonds on the nanorods surface. This interaction induced charge accumulation and altered the conductivity of the sensing layer, providing a robust signal for gas detection.

In another study from Li et al., the authors explored the fabrication and application of single-phase Au nanorods fabricated using NAA-assisted method, as a novel sensing material for detecting methyl mercaptan (CH_3_SH) gas [129]. The highly uniform nanostructures based on NAA platforms were fabricated using the same method as reported in their previous work [128]. In brief, the functionalization process involved depositing Au nanorods within the NAA template through pulse electrodeposition using a deposition solution containing HAuCl_4_·3H_2_O and boric acid. Key to the formation of single-phase Au nanorods was the modification of the deposition parameters, including a low pH of 1.33, the addition of 3.75 M dimethyl sulfoxide (DMSO) as a stabilizing agent, and a carefully controlled duty cycle of 10%. These conditions promoted uniform filling of the NAA pores and the growth of straight and smooth nanorods with a preferred (111) crystalline orientation. The Au nanorods were subsequently integrated into a sensor device using a combination of conductive silver adhesive and an Ag thin film for electrode contacts. The fabricated sensor demonstrated exceptional performance in detecting CH_3_SH gas, attributed to the self-assembled monolayer (SAM) property of these molecules onto Au, which facilitated strong interactions with the S-containing analyte. The sensor exhibited a current response of 0.88% at 0.5 ppm CH_3_SH, increasing to 7.82% at 4 ppm, with an LOD of 0.5 ppm. Notably, the single-phase Au nanorod sensor outperformed its Au thin-film counterpart, achieving sensitivity up to 21 times higher at 1 ppm CH_3_SH. Additionally, the sensor showcased superior selectivity, with significantly stronger responses to CH_3_SH compared to other gases such as H_2_S and N_2_.

Pt nanoporous films, renowned for their superior catalytic activity and high surface reactivity, provide an efficient platform for hydrogen gas detection by leveraging surface scattering phenomena and facilitating electron transfer processes, thereby significantly enhancing the sensitivity and selectivity of gas detection mechanisms—particularly suited for H_2_ gas in safety–critical applications. Sener et al. developed a resistive-type H_2_ sensor using nanoporous Pt films, which leveraged the enhanced catalytic activity and increased surface area provided by the metal and the NAA template. This in turn resulted in improved sensitivity and lower detection limits compared to those of conventional thin-film Pt sensors [138]. The high-uniformity NAA templates (nanopore diameter < 100 nm) were fabricated through a one-step anodization process using phosphoric acid as the electrolyte, operating at a voltage of 40 V and a temperature of 20 °C for 1 h. The NAA templates were then functionalized by depositing Pt films of two different thicknesses, 3 and 20 nm, using sputtering technique where the thickness of the films was precisely controlled via a quartz crystal microbalance. This thin-film deposition method facilitated the formation of highly porous Pt layers, optimizing the surface morphology for gas sensing applications. The H_2_ sensor structure was finalized by depositing Ag electrodes onto the Pt-coated NAA platform via thermal evaporation with a shadow mask, enabling the establishment of electrical contacts for resistance-based sensing measurements. The sensing performance of the nanoporous Pt–NAA films was then tested under varying H_2_ concentrations, from 10 to 50,000 ppm, and at different temperatures ranging from room temperature to 150 °C. Among the tested configurations, the 3-nm Pt film sensor exhibited the highest sensitivity, achieving a response of 13% at 10,000 ppm H_2_ at room temperature and demonstrating an LOD as low as 15 ppm. The 20-nm Pt sensor also performed well, with an LOD of 30 ppm, but showed reduced sensitivity compared to the thinner film. The study further explored the thermal effects on the sensor performance, revealing optimal operation at 50 °C for both configurations. However, at higher temperatures, the sensors exhibited diminished response due to reduced surface scattering phenomena. The H_2_ detection mechanism of the Pt films was attributed to surface scattering phenomena, wherein exposure to H_2_ gas decreases the number of scattered electrons on the Pt surface, thereby reducing resistance. The nanoporous structure of the NAA platform significantly enhanced the surface area-to-volume ratio of the Pt films, boosting their catalytic activity and facilitating efficient H_2_ adsorption and detection.

Pd has been a key material in the development of chemiresistive sensors, particularly for H_2_ detection, because of its excellent catalytic properties and ability to interact with H_2_ molecules at the atomic level [126, 144–148]. An early study from Elam et al. presented an H_2_ sensor, developed using ALD-deposited Pd films within high-aspect-ratio NAA platforms. The sensing system leveraged the unique hydride-forming properties of Pd and the extended surface area of NAA to achieve rapid and reproducible gas detection [126]. The NAA platforms, with pores measuring 40 nm in diameter and an aspect ratio of 1500, were fabricated via the two-step anodization process in 0.3 M oxalic acid at 3 °C. To enable efficient deposition of Pd films, a thin alumina seed layer, ranging from 1 to 10 nm, was deposited on the NAA surface using the ALD technique. This seed layer facilitated the formation of uniform and conformal Pd films within the high-aspect-ratio pores of NAA. Pd functionalization was achieved through sequential exposures to palladium hexafluoroacetylacetonate (Pd(hfac)_2_) and formalin (37% formaldehyde solution). The deposition conditions were optimized to ensure complete precursor penetration and consistent film growth, achieving a growth rate of 0.2 Å per ALD cycle. Electrical contacts were created by thermally evaporating Pd layers (~ 30 nm thick) through a shadow mask, with gold wires attached using silver paint. The resulting sensors exhibited a rapid response time of less than 1 s when exposed to H_2_, with an initial irreversible resistance drop attributed to enhanced inter-granular contact caused by H_2_-induced volume expansion. In subsequent exposures, the resistance change became reproducible and stable, demonstrating operational consistency. The sensors showed a resistance increase of approximately 20% upon H_2_ adsorption—a result consistent with continuous Pd films. This behavior was attributed to the formation of Pd-hydride, which induced volume expansion and enhanced inter-granular contacts within the Pd film, leading to significant resistance changes.

**Fig. 5:**
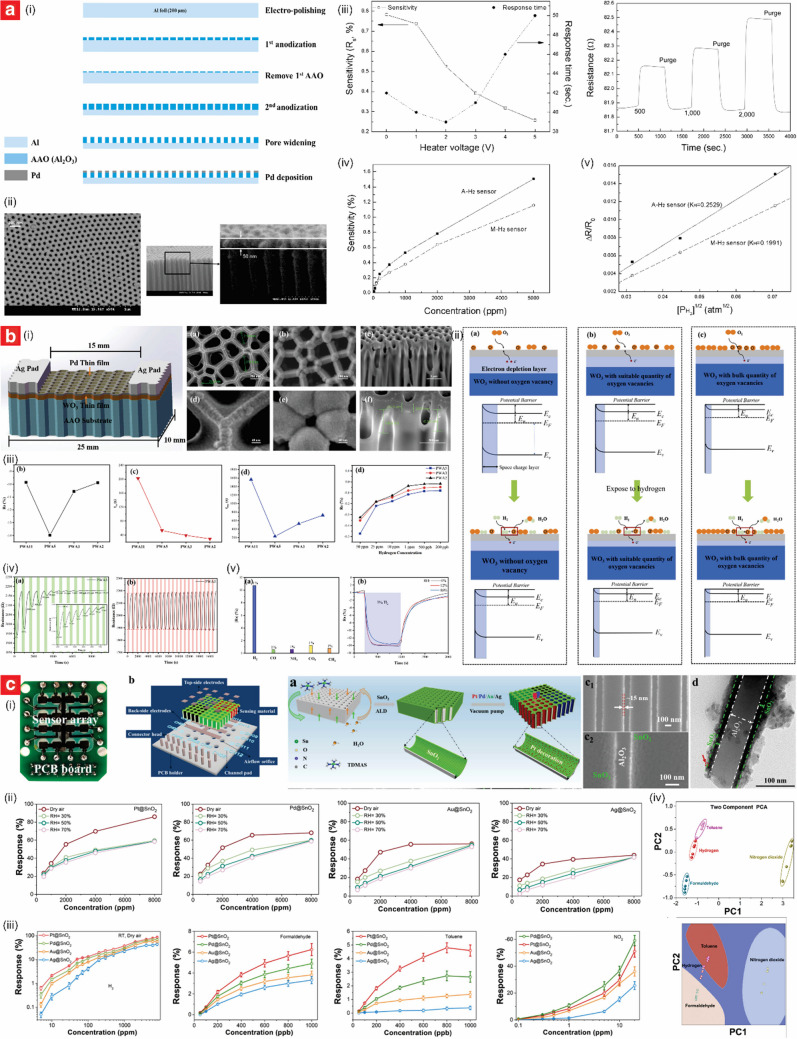
Engineering of NAA-based chemiresistive sensors functionalized with catalytic metals and metal oxides. **a** Engineering nanoporous Pd films on NAA templates via MEMS fabrication for enhanced H_2_ sensing: (i) flowchart for fabrication and (ii) SEM images of Pd films on NAA platform; (ii) transient response and response time of the sensor to H_2_; (iv) sensitivity curve; and (v) response rate curve of the sensor to various H_2_ concentrations (reproduced from ref. [146] with permission from Elsevier copyright 2014). **b** Engineering Pd/WO_3-x_/NAA sensors by tuning oxygen vacancy concentrations in WO_3-x_ films to enhance H_2_ detection at ppb levels: (i) schematic of the sensor design and SEM images of WO_3-x_/NAA sensor; (ii) H_2_ sensing reaction mechanisms of WO3-based sensor; (iii) response time and real-time response of the fabricated WO_3-x_/NAA sensors; (iv) real-time response of PWA5 sensor to ppb-level H_2_ concentrations; and (v) selectivity of the PWA5 sensor to different kinds of gases with the concentration of 1% (reproduced from ref. [131] with permission from Elsevier copyright 2024). **c** Engineering a 3D SnO_2_ nanotube-based sensor array on NAA templates with metal decoration to achieve room-temperature gas identification using machine learning algorithms: (i) structure of the sensor, fabrication process, and SEM images of 3D SnO2 nanotube with metal decoration; (ii) response toward H_2_ (500–8000 ppm) at different RH of the 3D nanostructured sensor with different metal decoration (i.e., Pt, Pd, Au, Ag); (iii) sensing response curves of the fabricated sensors towards H_2_, formaldehyde, toluene, and NO_2_; and (iv) gas classifications employing PCA and SVM algorithms (reproduced from ref. [156] with permission from Elsevier copyright 2023)

Kim and co-workers developed a highly sensitive H₂ gas sensor operating at elevated temperatures, based on Pd films deposited on NAA templates fabricated via the two-step anodization process (Fig. 5a) [146]. The NAA templates, prepared in 0.3 M oxalic acid at 40 V and 5 °C, featured nanopore diameters of 50 nm in average, achieved through a post-anodization pore widening treatment in phosphoric acid. The functionalization of the NAA templates involved the deposition of a 50 nm-thick Pd film using radio frequency (RF) magnetron sputtering for precise control over the film thickness and morphology, ensuring a conformal coating on the NAA’s nanoporous structure. The Pd-coated NAA platforms were integrated into a MEMS-based system, which included patterned Pt thin films serving as electrodes and micro-heaters. This design facilitated efficient H_2_ sensing by leveraging the high surface area of the nanoporous Pd film. The H_2_ detection performance was thoroughly characterized. At room temperature (i.e., heater voltage = 0 V), the sensor exhibited a sensitivity of 0.783% at 2000 ppm of H_2_, with an LOD of 25 ppm. The sensitivity varied across H_2_ concentrations, ranging from 0.135% at 100 ppm to 1.507% at 5000 ppm. A rapid response time of 42 s was observed at room temperature, corresponding to 90% of the signal change. At an elevated temperature of approximately 70 °C (i.e., heater voltage = 2 V), the sensitivity slightly decreased due to reduced H_2_ solubility in Pd, which was consistent with Sievert’s law. This temperature-dependent behavior highlighted the dynamic interplay between H_2_ adsorption and desorption on the Pd surface, which influenced the overall performance of the sensor. The H_2_ detection mechanism relied on the formation of Pd-hydride upon H_2_ adsorption, which induced lattice expansion and altered inter-granular contacts, leading to measurable resistance changes. In addition, the nanoporous structure of the Pd film significantly enhanced sensitivity by providing a larger surface-to-volume ratio, facilitating efficient H_2_ adsorption and interaction.

Building upon the advances in H_2_ sensing with Pd catalytic metal functionalization, combining Pd with other metals (e.g., Ag, Mg, and Bi) to create bi-metallic functional layers has introduced novel synergistic effects that enhance sensing stability, sensitivity, and operational range [139–141]. Hassan and Chung explored an H_2_ sensor design that utilized the unique properties of Pd-capped Mg ultra-thin films supported on hydrophobic alumina substrates [140]. The hydrophobicity, achieved through surface modification, increases the contact angle of the sensing substrate to 113.1°, significantly improving its resistance to environmental humidity and enhancing sensor stability. The functional sensing layer comprised Pd-capped Mg films fabricated via RF magnetron sputtering, with discrete quantum-sized nanoparticles uniformly distributed across the substrate. Precise deposition rates ensured optimized film thicknesses for efficient gas interaction, with Pd and Mg layers sputtered at rates of 1 nm/3.5 s and 1 nm/5.42 s, respectively. Silver electrodes were applied with a 2-mm gap to facilitate resistance-based sensing measurements. The sensor exhibited remarkable H_2_ detection capabilities, achieving a broad detection range of 1–40,000 ppm with rapid response, and recovery times of ~ 6 s and ~ 33 s, respectively, for 10,000 ppm H_2_ at room temperature. The H_2_ detection mechanism involved catalytic dissociation of H_2_ molecules on the Pd surface, followed by the diffusion of H_2_ atoms into the Mg layer to form MgH_2_. This process induced lattice expansion and increased resistance because of electron scattering. The hydrophobic surface and discrete nanoparticle structure further enhanced gas adsorption and desorption, contributing to the fast and reversible response of the sensor. In addition, the hydrophobic modification minimized the impact of RH up to 60%, preserving performance and ensuring operational stability over 5 months. The sensor also displayed excellent selectivity for H_2_ against other gases such as O_2_, CO, CO_2_, and nitrogen dioxide (NO_2_).

More recently, Du et al. introduced an innovative approach to enhancing the stability and performance of H_2_ sensors using palladium–bismuth (Pd–Bi) nanowires [141]. The nanowires were fabricated through NAA-template-confined co-electrodeposition, followed by chemical etching to create rough surfaces. This dual-step process resulted in nanowires with an average diameter of ~ 45 nm and a bristled surface morphology. This provided an optimized structure for H_2_ sensing applications. The functionalization process involved forming a Pd–Bi alloy during electrodeposition, with an initial Bi content of 15 at.%. Subsequent chemical etching reduced the Bi concentration to ~ 3 at.%, exposing more Pd atoms for H_2_ adsorption and enhancing the surface roughness and effective area. This morphological modification provided ample space to accommodate lattice expansion during H_2_ adsorption—a critical factor in suppressing the α–β phase transition and improving sensing stability. To engineer the sensor, multiple rough-surfaced Pd–Bi nanowires (RS-PdBi NWs) were integrated between interdigitated electrodes (IDEs). These nanowires formed conductive paths across the electrodes, enabling efficient transmission of electrical signals for the detection of changes in their electrical properties upon exposure to the analyte gas. The resulted sensors exhibited excellent H_2_ detection performance across a wide temperature range of 193.4–400 K. The sensors demonstrated a detection capability down to 0.1% (1000 ppm) hydrogen concentration, with transient response curves confirming fast and reversible performance. The H_2_ sensing mechanism was attributed to the synergistic effects of Bi doping and the rough surface morphology. Bi atoms altered the electronic structure of Pd, enriching its electron density and reducing excessive hydrogen penetration. The rough surface provided more active sites for H_2_ adsorption and additional space to accommodate PdHx expansion, enhancing sensitivity and stability while suppressing reverse sensing behavior at low temperatures.

#### Metal oxides

Metal oxides have been extensively employed in NAA-based chemiresistive sensors, including tungsten oxide (WO_3_) [131, 149], titanium oxide (TiO_2_) [132, 150–152], manganese oxide (Mn_3_O_4_) [133], zinc oxide (ZnO) [134], and tin oxide (SnO_x_) [135, 153], leveraging their semiconducting properties and ability to interact with target gases.

WO_3_, for example, has been utilized for its ability to introduce oxygen vacancies that enhance gas adsorption and improve charge carrier density. Zhang et al. demonstrated an H_2_ sensor with improved performance based on a Pd/WO_3–x_/NAA platform configuration, achieved by introducing oxygen vacancies into WO_3_ thin films to enhance sensitivity and selectivity at low hydrogen concentrations (Fig. 5b) [131]. The NAA template used for the sensor featured a pore size of 250 nm and a depth of 15 μm. WO_3–x_ thin films were deposited using reactive magnetron sputtering, with oxygen vacancy concentration controlled by adjusting the Ar:O_2_ flow ratio. A 5-nm Pd catalytic layer was subsequently deposited via DC magnetron sputtering to complete the sensor structure. The introduction of oxygen vacancies reduced the bandgap, increased electron concentration, and provided additional active sites for gas adsorption, facilitating improved sensing through the formation of an electron depletion layer. The Pd layer further enhanced H_2_ adsorption and dissociation, reducing the activation energy required for the sensing reaction. The Pd/WO_3–x_/NAA sensor demonstrated remarkable H_2_ sensing capabilities, achieving an LOD as low as 100 ppb. Among the tested configurations, the sensor with an Ar:O_2_ ratio of 5:1 (PWA5) exhibited optimal performance, with a response of 13.97% at a H_2_ concentration of 3%. The response and recovery times were 53 s and 231 s, respectively, indicating rapid and reliable detection. The sensor also showed high stability over 20 testing cycles and excellent selectivity for H_2_ compared to other gases such as CO, CO_2_, NH_3_, and CH_4_.

TiO_2_-based sensors have also seen notable advancements, with research highlighting their role in redox reactions because of their stable semiconducting properties. For instance, Lu and Chen introduced a high-temperature resistive H_2_ sensor based on a thin nanoporous rutile TiO_2_ film supported by an NAA platform [151]. The NAA substrate was fabricated through a two-step anodization process in 0.3 M oxalic acid under 40 V, followed by a pore widening process in 0.6 M phosphoric acid for 25 min at room temperature to achieve a well-defined self-organized porous structure with enhanced specific surface area. A thin TiO_2_ film, with varying thicknesses of 25 nm, 50 nm, and 100 nm, was deposited on the NAA using electron-beam evaporation of Ti, followed by oxidation and sintering at 600 °C in flowing oxygen to produce the rutile phase. The sensor was completed with platinum interdigitated electrodes fabricated via photolithography for electrical contacts. The functionalization of the NAA with rutile-phase TiO_2_ leveraged its thermal stability and high surface area for effective H_2_ detection. The sensor demonstrated exceptional performance, with conductance changes of 25 to 90 times in response to H_2_ concentrations ranging from 5 to 500 ppm. The LOD was as low as 5 ppm, and the device exhibited rapid response and recovery times of 10 s or less while maintaining stability under high-temperature conditions. The sensing mechanism was attributed to the “spill-over” effect, where H_2_ molecules adsorbed onto the platinum electrodes dissociated into atoms, which then diffused into the TiO_2_ lattice. This interaction caused partial electron transfer, resulting in significant changes in the conductance of the sensing layer. The porous NAA structure played a critical role by enhancing the specific surface area, which amplified the interactions between H_2_ molecules and the sensing material.

Similarly, Lu et al. presented a novel approach to fabricating macroporous TiO_2_ gas sensors for O_2_ detection via NAA-assisted method [132]. The NAA layer was formed on aluminum-coated silicon wafers using anodization in 0.3 M oxalic acid at 4 °C under an applied voltage of 40–60 V, followed by immersion in 5% phosphoric acid for 4 h to widen the pores and remove the BOL. This process resulted in a macroporous surface with pore diameters ranging from 100 to 750 nm, providing a high specific surface area to enhance the sensor performance. A 50-nm TiO_2_ thin film was deposited on the macroporous substrate via RF sputtering, forming the active sensing layer. The electrical contacts were established using silver electrodes fabricated by a lift-off technique. The unique combination of the macroporous structure and the TiO_2_ film aimed to improve gas-sensing characteristics, specifically targeting O_2_ detection. The fabricated sensor demonstrated superior performance at a high operating temperature of 500 °C. The sensing mechanism relied on the n-type semiconductor properties of TiO_2_, where O_2_ adsorption on the surface created a depletion layer, affecting charge carrier concentrations and resistance. Compared to its non-porous counterparts, the macroporous TiO_2_ sensor exhibited approximately 33% higher sensitivity, effectively discriminating between O_2_ concentrations ranging from 4000 to 6000 ppm. The enhanced performance was attributed to the increased specific surface area, which amplified electron interactions with the gas molecules, and the improved stability and noise reduction of the macroporous architecture.

Mn_3_O_4_, a lesser-studied metal oxide, has been applied for ethanol and acetone detection at room temperature. John et al. used a vacuum infiltration technique to grow aligned Mn_3_O_4_ nanorods within NAA templates, showing enhanced sensitivity attributed to increased surface area and oxygen vacancies [133]. The commercially acquired NAA substrate, characterized by an average pore diameter of 350 nm and a pore length of 60 μm, served both as a template and a support for the gas sensor. Mn_3_O_4_ nanorods were synthesized via a hydrothermal method, using manganese acetate (Mn(CH_3_COO)_2_) and ammonium hydroxide (NH_4_OH) precursors, followed by vacuum infiltration to fill the NAA pores. Subsequent annealing at 400 °C in an argon-rich atmosphere resulted in crystalline Mn_3_O_4_ with a highly uniform, aligned structure. These nanorods extended the entire length of the NAA pores, forming a continuous and well-organized array. Gold electrodes were sputtered onto the top and bottom surfaces of the Mn_3_O_4_-filled NAA platform to create a conductometric sensor. The sensor demonstrated outstanding gas sensing performance, with maximum sensitivities of 67% and 68% for ethanol and acetone vapors, respectively, at a concentration of 50 ppm. For lower concentrations of 25 ppm, the sensitivities were 56% for ethanol and 59% for acetone. The response times were exceptionally fast, with 4 s for ethanol and 2 s for acetone at 50 ppm, and 7 s for ethanol and 5 s for acetone at 25 ppm, respectively. Recovery times varied depending on the gas and concentration, ranging from 64 s for acetone at 50 ppm to 70 s for ethanol at 25 ppm. Remarkably, these results were achieved at room temperature, emphasizing the practicality of the sensor for ambient applications. Furthermore, the sensor demonstrated excellent stability, with sensitivity variations of only ± 0.4% for ethanol and ± 0.3% for acetone over a testing period of two months. The enhanced sensing capability was attributed to the large surface area and oxygen vacancies of the Mn_3_O_4_ nanorods, which facilitated the adsorption of O_2_ molecules on their surfaces and created negative oxygen ions that form a depletion layer, increasing the resistance. When exposed to reducing gases such as ethanol and acetone, reactions with the chemisorbed O_2_ released electrons back into the conduction band, decreasing the resistance and enabling rapid detection.

ZnO nanostructures, synthesized via NAA-assisted template routes, have demonstrated excellent room-temperature gas sensing properties. Kumar et al. utilized a low-temperature synthesis method to fabricate luminescent ZnO nanowires on NAA template, leveraging their high electron mobility and intrinsic defect chemistry for efficient room-temperature NH_3_ detection [134]. The NAA templates featured nanopores with diameters of 45–50 nm and a thickness of 12 µm, prepared through anodization (i.e., 0.3 M oxalic acid at 40 V and 3 °C) followed by pore widening in 6 wt% phosphoric acid solution. Vertically aligned ZnO nanowires were formed within the NAA pores via a vacuum infiltration technique using a saturated zinc nitrate (Zn(NO_3_)_2_) solution, followed by drying and annealing at 435 °C to achieve the wurtzite-phase ZnO structure. The sensor was fabricated by integrating the ZnO nanowires in the gap between two Cu wire electrodes using a micromechanical technique. The sensor achieved a sensitivity of 68% for 50 ppm NH_3_, with response and recovery times of approximately 28 and 29 s, respectively, and exhibiting a linear sensitivity that was maintained up to 75 ppm, while the response increase slowed at higher concentrations. The sensing mechanism was attributed to the chemisorption of oxygen ions (O_2_⁻) on the ZnO nanowire surfaces, which altered the surface charge distribution and reduced conductivity. Upon exposure to NH_3_, reactions between the gas molecules and the adsorbed oxygen ions released electrons back into the conduction band, increasing its conductivity. The high performance of the sensor was enhanced by the Debye length of ZnO nanowires, which was comparable to their radius (~ 34 nm), amplifying the impact of surface interactions on electrical properties.

SnO_x_ materials, including SnO and SnO_2_, have also been incorporated into NAA platforms for the development of gas sensors, making a good use of their high surface-to-volume ratios and tunable electronic properties. Zheng et al. synthesized < 20 nm SnO nanowires through thermal oxidation, using NAA templates as a structural framework to enhancing sensitivity through increased reactive surface area and improved gas adsorption dynamics [153]. The NAA templates, featuring pore diameters of approximately 20 nm and lengths of 20–22 µm, were prepared through the two-step anodization process in 2.6 wt% sulfuric acid at 5 °C, followed by pore widening treatment in 6 wt% phosphoric acid for 20 min at 30 °C. To enable electrical contact, a platinum layer (~ 400 nm) was sputtered onto the NAA platforms as a conductive base prior to the deposition process. Sn nanowires were synthesized within the NAA pores via electrochemical deposition using a solution of 0.01 M SnCl_2_ and 0.05 M H_3_BO_3_, employing a combination of AC and DC voltages. The resultant composite structures were subsequently annealed in vacuum-sealed glass ampoules at 500 °C for 4 h, converting them into crystalline SnO. The final sensor configuration involved integrating the SnO nanowires onto a platinum interdigitated electrode, which was placed in a vacuum chamber to minimize atmospheric interference. The sensor exhibited a resistance change rate of 49% when exposed to 5000 ppm of CO_2_ gas at room temperature, with a response time of approximately 5 min. This performance underscored the high surface area and sensitivity of the SnO nanowires, driven by their nanoscale dimensions and the enhanced interaction between gas molecules and the active sensing surface. The SnO nanowire-based CO_2_ sensor operated through a resistance modulation mechanism driven by surface interactions with the target gas. Adsorbed oxygen species in the air initially depleted charge carriers in the p-type SnO, but their reaction with CO_2_ restored electrons, reducing resistance and enabling sensitive detection correlated with gas concentration.

Lu et al. introduced Pd-doped SnO₂ nanowires within porous alumina substrates, which lowered operational temperatures and boosted H_2_ sensing by leveraging catalytic activity of Pd for improved charge transfer efficiency [135]. The synthesis involved reacting SnCl_4_·5H_2_O in a solution of oleic acid and oleylamine at 180 °C for 8 h, followed by annealing at 350 °C to remove organic ligands and create a porous network of nanowires. Pd doping was achieved by treating the SnO_2_ nanowires with a PdCl_2_ solution, significantly enhancing their catalytic properties. The sensors were fabricated by drop-coating the SnO_2_ nanowires onto alumina substrates equipped with gold electrodes spaced 200 µm apart, followed by annealing to stabilize the sensing film. The H_2_ sensing capabilities of SnO_2_-based sensors, particularly with the addition of Pd doping, exhibited significant improvements in sensitivity, response time, and operating conditions. The sensor demonstrated a strong response of 8.5 to 40 ppm H_2_ at an optimized operating temperature of 150 °C, with a LOD as low as 500 ppb, corresponding to a response of approximately 1.15. The response and recovery times were markedly improved with Pd doping; without doping, these times were 13 s and 15 s, respectively, for 40 ppm H_2_, whereas with Pd doping, the time reduced significantly to 6 s and 3 s, respectively. Further to that, the optimal operating temperature was lowered from 250 to 150 °C because of Pd doping, with potential operation near room temperature, albeit at a slightly reduced sensitivity. H_2_ detection in Pd-doped SnO_2_ sensors involved dynamic interaction between adsorbed oxygen species and H_2_ molecules. The pre-adsorbed oxygen extracted electrons from the SnO_2_ conduction band. Upon exposure to H_2_, Pd dopants catalyzed the chemical reaction between H_2_ and the adsorbed oxygen species, leading to a more efficient electron release back into the SnO_2_ conduction band, thereby enhancing sensitivity and reducing operating temperatures.

Building on the advancements of single-metal oxide sensors, composite and hybrid metal oxides, which integrate multiple metals within a single oxide structure, have demonstrated enhanced performance attributed to synergistic effects between their components [136, 154–156]. For instance, in a study by Chen and co-workers, Fe–NiO_x_ nanotubes fabricated through aqueous chemical precipitation and calcination within NAA templates exhibited ultralow detection limits for H_2_S. This performance was attributed to enhanced electron mobility and reactive oxygen species generation at the Fe–NiO_x_ interface [136]. The commercially acquired NAA template facilitated the assembly of Fe–Ni layered double hydroxides (LDHs) into nanotubes, which were subsequently calcined to form Fe–NiO_x_. The authors emphasized the advantages of this structure, including a high specific surface area of 175 m^2^⋅g^–1^ and uniform Fe^3+^ doping, which enhanced the sensing performance of the material. The calcination process also created abundant oxygen vacancies, further contributing to the sensor’s efficiency. The fabrication of the sensing device involved integrating the Fe–NiO_x_ nanotubes onto a MEMS platform with interdigital electrodes, followed by heat treatment at 250 °C. The gas sensor demonstrated remarkable sensitivity, achieving an *ΔR/R*_*a*_ value of 5.24 for 0.8 ppm H_2_S at an optimal operating temperature of 270 °C. The LOD reached was as low as 0.05 ppm, and the sensor exhibited rapid response and recovery times of 3.2 s and 8.1 s, respectively. Its selectivity for H_2_S over interfering gases such as NO_2_ (i.e., selectivity coefficient S_H₂S_/S_NO₂_ = 8.3) was attributed to Fe^3+^ doping, which shifted the adsorption enthalpy to favor H_2_S detection. The long-term stability of the sensor was validated over 40 days, maintaining consistent performance. The study also integrated density functional theory (DFT) calculations, which corroborated the experimental results by demonstrating the critical role of Fe^3+^ in modifying the adsorption properties and selectivity of the NiO_x_ surface.

In another study by Gorokh and co-workers, Sn_x_Bi_k_Mo_y_O_z_ nanocomposites produced via cyclic ionic layering on NAA platforms demonstrated micropowered sensing capabilities with improved selectivity for toxic gases, benefiting from the electronic coupling between the constituent metals [155]. A well-controlled double-sided anodization process in 0.5 M oxalic acid at 14–15 °C and 55 V was used to fabricate NAA substrates with a uniform pore structure. The produced membranes featured pore diameters ranging from 30 to 50 nm, and porosity ~ 20%. The functionalization of the NAA membranes was achieved using cyclic ionic layering, alternating between Sn–Mo oxides and Bi–Mo oxides. Twenty monolayers were deposited in total, followed by annealing at 480 °C in an argon atmosphere, resulting in a crystalline nanostructure. The sensitive layer was coupled with a platinum-based heating and electrode system, fabricated through magnetron sputtering and photolithography, ensuring effective electrical and thermal conductivity. The sensor demonstrated excellent sensitivity to reducing gases, specifically H_2_ and CO. For H_2_ detection within the range 5–50 ppm, the sensor achieved sensitivities of 0.22 and 0.40 at concentrations of 5 ppm and 40 ppm, respectively, at an optimal operating temperature of 250 °C with a power consumption of only 10 mW. For CO, the sensor detected concentrations ranging from 1 to 100 ppm at a higher operating temperatures of 400 °C. The sensor operated efficiently within a wide temperature range (i.e., 150–550 °C) and exhibited rapid response and recovery times, approximately 5 min each. These characteristics highlight its potential for real-time monitoring of toxic and flammable gases. The sensing mechanism was attributed to the n-type semiconducting behavior of the Sn_x_Bi_k_Mo_y_O_z_ composite. Interaction with reducing gases led to chemisorption on the oxide surface, reducing resistance by enriching the conduction band with electrons. The incorporation of a well-structured NAA membrane minimized power consumption and enhanced thermal stability, making the sensor suitable for low-energy applications.

Composite metal oxides have also shown remarkable potential in enhancing the performance of NAA-based chemiresistive gas sensors by leveraging heterojunction and interface engineering. Kumar et al. presented the design of a SnO_2_/CuO bilayered system deposited on NAA substrates utilizing a p–n heterojunction at the oxide interface. This created a depletion region that improved charge transfer efficiency and selectively modulated conductivity upon CO exposure [154]. The NAA structure was fabricated through anodization in 0.3 M oxalic acid at 0 °C under a constant voltage of 80 V for 1 h, creating a nanoporous framework for the sensing layer. The bilayered thin film was synthesized using pulsed laser deposition (PLD) at 100 °C in an oxygen environment, employing sequential ablation of SnO_2_ and CuO targets. The functionalized SnO_2_/CuO bilayer demonstrated remarkable gas sensing performance over tested CO concentrations, ranging from 5 to 500 ppm, achieving a maximum response of *R*_*a*_*/R*_*g*_ = 9.5 at 180 °C for 100 ppm CO. It exhibited rapid response and recovery times of 18 s and 78 s for these concentrations, respectively, and maintained high selectivity for CO over other gases such as H_2_ and NH_3_. Additionally, the sensor’s performance remained stable over 90 days, indicating excellent long-term reliability. The sensing mechanism was attributed to the formation of a p–n heterojunction between p-type CuO and n-type SnO_2_, which enhanced the modulation of charge carriers at the interface and thereby improved the sensor’s sensitivity. The hydrophobicity of the SnO_2_/CuO bilayer also contributed to the observed reduced interference from humidity. The interaction of CO with chemisorbed oxygen species on the bilayer surface reduced resistance by releasing electrons into the SnO_2_ conduction band, effectively increasing the response of the sensor. In addition, the p–n heterojunction played a critical role in amplifying this effect, enabling detection at lower temperatures compared to SnO_2_ alone.

Complementing this, metal-decorated 3D SnO_2_ nanotube arrays, fabricated via ALD, incorporated noble metals (e.g., Pt and Pd) to form Schottky barriers at the metal–SnO_2_ interfaces (Fig. 5c) [156]. Yan and Song demonstrated an innovative room-temperature gas sensor array based on 3D SnO_2_ nanotubes decorated with noble metals (i.e., Pt, Pd, Au, Ag) and supported by a NAA substrate. The NAA structure provided a robust platform with a high surface-area-to-volume ratio, featuring a 40-µm-thick porous layer with an average pore size of 400 nm. SnO_2_ nanotubes were fabricated using ALD at 150 °C, utilizing tetrakis(dimethylamino)tin (TDMAS) as the precursor and water vapor as the oxygen source. Post-deposition, the nanotubes were decorated with a 5-nm-thick metal layer applied via a vacuum pumping method, leveraging the catalytic properties of these metals to enhance gas-sensing performance. The fabricated sensor array demonstrated excellent gas-sensing capabilities at room temperature, detecting H_2_, formaldehyde, toluene, and NO_2_ with high sensitivity and selectivity. For H_2_, the detection range spanned from 5 to 8000 ppm, achieving a response of 17.4% at 200 ppm with Pt decoration. The LODs for formaldehyde and toluene were as low as 50 ppb, while NO_2_ detection reached a limit of 100 ppb. These results highlighted the ability of this sensor design to monitor gases critical to air quality and safety, with a minimal power consumption of 4 µW per sensor unit. The response and recovery times for H_2_ were 30 and 45 s, respectively, meeting industrial standards for real-time monitoring. The sensor’s performance was attributed to the metal–SnO_2_ interface, where Schottky barrier modulation played a key role. Metals such as Pt and Pd, with high work functions, act as electron acceptors, forming energy barriers that modulate baseline resistance. Gas adsorption and dissociation, facilitated by the catalytic properties of the metal decorations, led to reactions with pre-adsorbed oxygen species on SnO_2_, releasing electrons into the conduction band and reducing resistance. This process, combined with the high surface-area-to-volume ratio of the SnO_2_ nanotubes, enhanced gas adsorption and sensitivity. In addition to its superior sensing performance, the sensor array incorporated advanced features such as pattern recognition algorithms for gas identification. Using techniques such as principal component analysis (PCA) and support vector machines (SVM), the sensor array achieved accurate classification of multiple gases, overcoming the selectivity limitations often faced by single sensors. These results demonstrated the potential of metal-decorated SnO_2_ nanotube arrays for applications in environmental monitoring and smart home systems, offering a low-power, highly sensitive solution for real-time gas detection.

#### Carbon-based materials

Carbon nanomaterials, including carbon nanotubes (CNTs) and graphene, are renowned for their exceptional electrical, thermal, and mechanical properties, making them a versatile choice for improving gas sensor performance. Their high electrical conductivity and extensive surface area facilitate efficient gas adsorption and charge transfer processes—key parameters in achieving high sensitivity and rapid response in gas sensing applications. When combined with the unique structural properties of NAA, including its high porosity, uniform pore distribution, and tunable geometry, these materials create hybrid sensors that exhibit synergistic advantages over other architectures. The porous NAA matrix provides an ideal scaffold for anchoring carbon materials, promoting uniform distribution and enhanced gas diffusion to active sites. At the same time, carbon nanomaterials amplify the transduction mechanisms and functionality by converting gas adsorption events into measurable electrical or optical signals.

Aligned CNTs deposited via CVD within NAA platforms have been employed for H_2_ and NH_3_ sensing, leveraging their excellent charge transport properties [157–159]. An early study on the combination of CNTs integrated with NAA templates explored the development of H_2_ sensors, featuring a Pd nanoporous film that enhanced sensitivity and broadened the detection range compared to traditional dense Pd films [157]. The NAA template was fabricated using the two-step anodization process in 0.3 M oxalic acid, yielding nanopores with diameters of 60–70 nm and lengths of approximately 1 µm. Functionalization of aligned CNTs was performed through pyrolysis of acetylene at 650 °C, and Pd and Au nanoporous films were deposited on the top surface as electrodes using RF sputtering and thermal evaporation to complete the sensor structure. The sensing mechanism relied on the H_2_-trapping properties of the Pd nanoporous film, which facilitated resistance changes upon interaction with H_2_ molecules. The CNTs provided mechanical stability and enhanced the interaction area for the Pd film. The study revealed significant differences in the performance of Pd- and Au-based sensors. While Au-electrode CNT sensors demonstrated no response to H_2_, the Pd-electrode CNT sensors exhibited sensitivity to medium H_2_ concentrations, ranging from 0.1 to 1.5%. The steady-state response of the Pd-electrode CNT sensors, measured as the relative resistance change (*ΔR/R*_*0*_), increased proportionally with H_2_ concentration, with response times of approximately 3–4 min. Additionally, the CNT-supported Pd film sensors demonstrated superior performance, detecting H_2_ at both diluted and medium concentrations (i.e., 100 ppm to 1.5% H_2_). For example, at 100 ppm H₂, the response was 0.17%, which increased to 4.2% at 1.5% H₂. However, for concentrations above 1.5%, the sensor performance degraded due to film instability caused by hydrogen-induced volume expansion.

Integration of CNTs with highly uniform NAA templates carried out by Hoa and co-workers underscored the potential of this hybrid system for reliable NH_3_ detection and mass production capabilities [158]. The NAA template was fabricated through the two-step anodization process in 0.3 M oxalic acid at 40 V and 10 °C, yielding highly ordered nanochannels with a uniform pore diameter of approximately 65 nm, inter-pore distance of 105 nm, and length of 1.4 µm. The multi-walled CNTs (MWCNTs) were then synthesized directly on the pore wall of NAA templates via CVD without requiring metallic catalysts. The synthesis was performed at two different temperatures, 900 and 1200 °C, and involved a post-growth oxygen plasma etching step to open the pore ends for the higher-temperature synthesis. The functionalization of the NAA template with CNTs leveraged the structural regularity of the nanochannels to produce an array of vertically aligned CNTs with consistent geometries. This innovative vertical transport configuration enhanced gas diffusion into the pores for effective adsorption. Silver electrodes were sputtered onto the surface of the CNT-embedded NAA platform to create electrical contacts. In terms of performance, sensitivity measurements highlighted the influence of synthesis temperature and defect density. At an applied voltage of 5 V and 6% NH_3_ concentration, CNTs synthesized at 900 °C exhibited a sensitivity of approximately 35%, while oxygen-treated CNTs synthesized at the same temperature showed a reduced sensitivity of 20%. In comparison, CNTs synthesized at 1200 °C exhibited a lower sensitivity of 15%. The higher sensitivity of CNTs synthesized at 900 °C was attributed to their greater density of crystallographic defects, which acted as favorable adsorption sites for NH_3_. Oxygen-related defects introduced by plasma treatment degraded the sensitivity of the system to approximately 43% for the 900 °C CNTs (i.e., from 35 to 20%). This degradation in performance was attributed to reduced interactions between NH_3_ molecules and oxygenated defect sites compared to those existing in neutral nitrogen environments. It was found that the response times varied with the synthesis conditions. CNTs synthesized at 900 °C exhibited a response time of ~ 1.1 min, while oxygen plasma-treated CNTs synthesized at the same temperature demonstrated a faster response time of 0.3 min. CNTs synthesized at 1200 °C without oxygen treatment showed an intermediate response time of 0.5 min. These results suggested that oxygen-related defects introduced by plasma treatment enhanced the adsorption kinetics of NH_3_ molecules, facilitating faster response times. The sensor achieved a minimum detectable NH_3_ concentration of 0.1%, and the response showed a linear increase beyond ~ 1% NH_3_ concentration, indicating statistical adsorption behavior without saturation within the tested range.

**Fig. 6:**
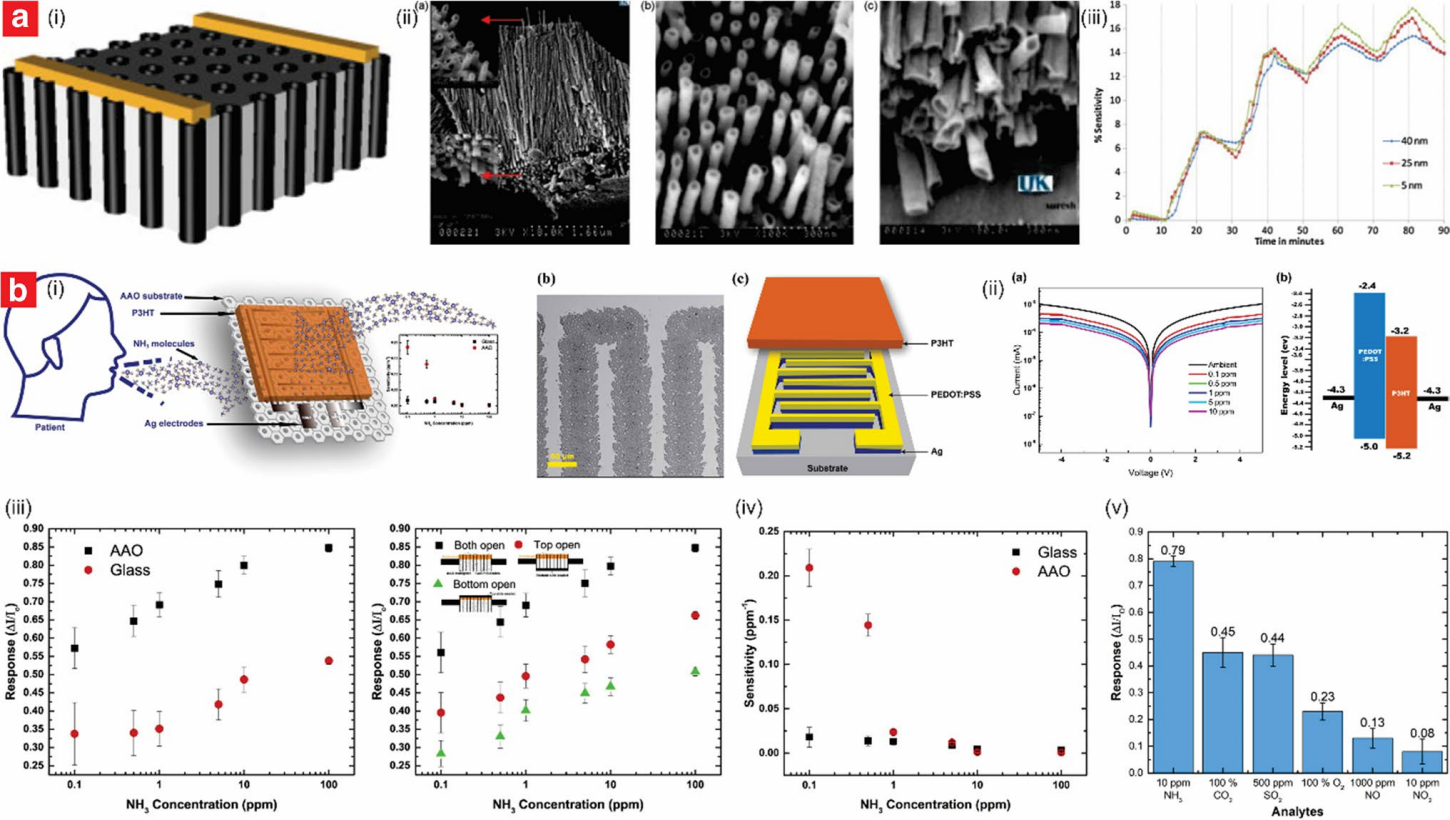
Engineering of NAA-based chemiresistive sensors functionalized with carbon-based materials and semiconducting polymers. **a** Engineering MWCNT/NAA resistive sensors via CVD and amorphous carbon layer optimization to enhance NH_3_ detection at room temperature: (i) schematic showing MWCNT/NAA sensor design with integrated gold bar electrodes; (ii) SEM images of the MWCNT on NAA platform; and (iii) response of MWCNT/AAO resistive sensor with different a-C layer thickness to 0.01% NH_3_ at room temperature (reproduced from ref. [159] with permission from Elsevier copyright 2010). **b** Development of P3HT-functionalized NAA-based sensor for NH_3_ detection at ppb levels: (i) schematic illustrating the sensing concept, and design and structural characterization via SEM images of the sensor; (ii) sensing mechanism of the sensor based on peak intensity change in I-V scans and energy band modification of the sensing material; (iii) sensing responses of the sensors fabricated with different configurations (i.e., glass or NAA platform, and with open top/bottom configuration) to varying concentrations of NH_3_; and (iv) sensitivity analysis and (v) selectivity analysis of the sensor (reproduced from ref. [163] with permission from Elsevier copyright 2022)

Similarly, Mangu et al. demonstrated the development of a resistive NH_3_ sensor based on MWCNTs embedded in NAA templates, achieving improved sensitivity through a high surface area design and tunable amorphous carbon (a-C) layers that enhanced gas adsorption and charge transfer efficiency (Fig. 6a) [159]. The NAA templates were fabricated using the two-step anodization process in 0.3 M oxalic acid at 40 V and 5 °C, resulting in hexagonally arranged cylindrical nanopores with diameters of 35 nm. The BOL at the bottom of the pores was removed by phosphoric acid etching to ensure open pores for CNT growth. The MWCNTs were synthesized using catalyst-free CVD from pure xylene pyrolyzed at 700 °C, yielding vertically aligned nanotubes that conformed to the NAA pore geometry. A thin layer of a-C, formed as a by-product during the CVD process, was deposited on both sides of the template. This a-C layer played a critical role in sensor performance, acting as a conductive bridge between adjacent nanotubes. The thickness of the a-C layer, which ranged from 5 to 40 nm, was tuned either through CVD process parameters or post-growth plasma oxidation. The sensor devices were integrated with gold-bar-electrodes for resistive sensing. The sensing mechanism relied on the p-type semiconducting behavior of MWCNTs, where the adsorption of NH_3_ molecules, as electron donors, increased the resistance of the device by reducing the number of charge carriers (holes). The sensitivity of the sensor improved significantly as the thickness of the a-C layer decreased, with cumulative sensitivities of 15.37, 16.86, and 17.7% observed for a-C layers of 40, 25, and 5 nm, respectively, when exposed to 0.01% NH_3_. This improvement was accompanied by a concurrent increase in baseline resistance, which rose from 2.78 kΩ for the 40 nm a-C layer to 9.27 kΩ for the 5 nm layer. The well-defined pore structure and vertically aligned nanotubes provided a large surface area for gas adsorption, enhancing the sensor’s responsiveness and reproducibility.

In another study, Yang et al. utilized graphene layers grown on quasi-periodic NAA structures for developing gas sensors with enhanced gas adsorption and charge carrier mobility. These resulted in significant improvements in sensitivity and selectivity for detecting reducing and oxidizing gases [137]. NAA substrates featuring a periodic honeycomb structure with pores of approximately 55 nm in diameter and a pitch of 110 nm were fabricated using the two-step anodization process in 0.3 M oxalic acid at 40 V and 15 °C. After anodization, the pore diameter was widened with a mild etching process in 0.1 M phosphoric acid to enhance the surface characteristics. The graphene used in the sensor was grown via low-pressure CVD (LPCVD) on copper foil and subsequently transferred onto the NAA substrates. The copper surface was prepared by annealing at 1000 °C under a hydrogen flow, followed by graphene synthesis using a methane-hydrogen gas mixture. A poly(methyl methacrylate) (PMMA)-assisted wet transfer process was employed to transfer the monolayer of graphene from the copper foil to the NAA substrates. For sensing device fabrication, the graphene was patterned into a channel geometry using a resist-free stencil mask and Ar ion beam irradiation. Gold (40 nm) and chromium (5 nm) electrodes were deposited onto the graphene channel through thermal evaporation with the stencil mask technique, ensuring precise alignment and minimal contamination. This meticulous fabrication process preserved the monolayer graphene’s intrinsic properties and enabled the integration of the NAA substrates for enhanced gas sensing performance. The sensor demonstrated significantly enhanced sensitivity to NO_2_ and NH_3_ compared to that of the graphene devices on traditional SiO_2_ substrates. For a gas concentration of 130 ppm NO_2_, the NAA-supported graphene sensor exhibited a conductance change of approximately 6%—three times higher than the 2% change observed for the SiO_2_-supported device. Similarly, for 1300 ppm NH_3_, the conductance change was 1.5% for the NAA-supported sensor, compared to 0.5% for the SiO_2_-supported sensor. This threefold enhancement in sensitivity was attributed to the inhomogeneous electrostatic potential landscape provided by the NAA substrate, which facilitated stronger physisorption of gas molecules. Additionally, wrinkles and folds in the graphene induced by the NAA substrate created extended defect states, further enhancing gas adsorption and interaction. These carbon-based materials highlight the potential of combining NAA with advanced conductive frameworks for high-performance gas sensing applications.

#### Semiconducting polymers

Semiconducting polymers have also expanded the functional landscape of NAA-based chemiresistive sensors due to their unique electrical properties, tunable chemical structures, and inherent flexibility. The effectiveness of these materials in gas sensing lies in their π-conjugated backbone structure, which enables delocalized electron movement and semiconducting behavior. This structure allows conducting polymers to respond dynamically to gas molecules through mechanisms such as charge transfer and doping/de-doping processes. Gas molecules interacting with the polymer can donate or withdraw electrons, altering the polymer’s electronic structure and conductivity. The redox-active nature of many conducting polymers, such as polyaniline and polypyrrole, facilitates reversible changes in oxidation states upon exposure to oxidizing or reducing gases [160, 161]. Surface functional groups on the polymer chains provide active sites for specific interactions, enhancing sensitivity and selectivity. When integrated with the high surface area and porous structure of NAA, the gas adsorption is further amplified, ensuring effective interactions between the polymer and the target gases. These combined properties make semiconducting polymers a versatile and efficient functional material for NAA-based gas sensors.

Dan et al. developed poly(3,4-ethylene-dioxythiophene)/poly(styrene-sulfonate) (PEDOT/PSS) striped-structure nanowires assembled within NAA templates via di-electrophoresis, leveraging the improved charge transport and swelling of polymer nanostructures upon gas adsorption for VOC detection [162]. The NAA templates provided a well-defined structure with a pore diameter of approximately 220 nm, enabling the fabrication of “striped” nanowires with alternating segments of PEDOT/PSS and Au. These nanowires were constructed using a combination of electrodeposition and dielectrophoretic assembly techniques. The PEDOT/PSS segment, which acted as the active sensing material, was deposited under a constant potential in a solution containing the monomer and poly(styrenesulfonic acid) dopant, which played a crucial role in stabilizing the polymer matrix and preventing unwanted contamination during electrodeposition. The integration of the nanowires into sensing devices was achieved by dielectrophoretic assembly between gold electrodes on silicon chips, ensuring effective electrical contact and alignment, while silver paste was applied post-assembly to enhance electrical connectivity. This design facilitated the measurement of resistance changes upon exposure to model VOCs, including acetone, methanol, and ethanol. The sensor demonstrated notable resistance changes of 10.5%, 9%, and 4% for acetone, methanol, and ethanol, respectively, at their saturation vapor pressures, with response and recovery times of ~ 30 s. Additionally, the linear response observed for methanol concentrations, ranging from 10 to 50% of the saturated vapor pressure, highlighted their suitability for quantitative analysis. However, exposure to methanol concentrations exceeding 50% of the saturated vapor pressure caused irreversible changes in the nanowire resistance, likely due to polymer chain conformational changes. The sensing mechanism was attributed to two primary effects: (i) polymer swelling and (ii) charge carrier reduction in the PEDOT/PSS backbone upon gas adsorption. These interactions resulted in measurable changes in the nanowires’ electrical resistance, with the high surface-to-volume ratio of the nanowire structure amplifying the sensitivity and response speed compared to traditional thin-film sensors.

Biring and Kolaru employed poly(3-hexylthiophene-2,5-diyl) (P3HT) polymer to create NH_3_ sensors by spin-coating the polymer onto NAA templates. These sensors operated efficiently at a low bias of 1 V, benefiting from the strong dipole–charge interactions of P3HT with NH_3_ molecules and the enhanced gas exposure provided by the nanoporous structure of NAA (Fig. 6b) [163]. The NAA substrates, featuring 100-nm pores and a thickness of 60 µm, were commercially obtained and utilized as a platform for the deposition of P3HT—a semiconducting polymer known for its high sensitivity to NH_3_. A PEDOT:PSS hole-transporting layer was spin-coated onto the silver electrodes patterned on the NAA surface, followed by spin-coating of P3HT in chlorobenzene to complete the device fabrication. The interdigitated electrodes, laser-patterned onto the substrate, ensured robust electrical contacts while maintaining exposed nanopores for efficient gas diffusion. The sensor exhibited exceptional performance, detecting NH_3_ concentrations as low as 100 ppb, with a response of 0.56 at this level—representing a significant improvement over comparable sensors on glass substrates. The NAA-based device demonstrated rapid response and recovery times of 2 and 4 s, respectively, attributed to its high surface area and efficient gas diffusion through the nanopores. The sensing mechanism was based on charge–dipole interactions between NH_3_ molecules and the P3HT polymer, which reduced hole mobility and modulated the device’s current. The through-hole NAA structure further enhanced the interaction area by allowing gas molecules to access the sensing layer from both sides of the substrate. This dual-sided access enabled a dramatic increase in sensitivity, particularly at lower NH_3_ concentrations (< 20 ppm), where the sensor achieved a sensitivity of 0.21 ppm^–1^ at 100 ppb. In addition to sensitivity and dynamic response, the sensor exhibited strong selectivity for NH_3_ over other gases such as CO_2_, NO, NO_2_, and SO_2_, making it a promising candidate for industrial and environmental applications.

#### Other functional materials

The versatility of NAA allows it to be functionalized with a wide range of materials beyond conventional choices such as catalytic metals, metal oxides, conducting polymers, and carbon-based materials. These alternative functional materials offer unique properties that expand the scope of NAA-based gas sensing technologies.

One of the possibilities is the integration of ceramics, which add thermal stability and catalytic properties to NAA-based gas sensors. Pd-decorated silicon carbide (SiC) nano-cauliflowers, synthesized via DC magnetron sputtering on a NAA substrate, demonstrated efficient H_2_ sensing at high temperature [164]. The sensor was fabricated on a NAA substrate coated with a silver layer to ensure electrical conductivity. The SiC nano-cauliflowers were deposited using DC magnetron co-sputtering—a physical vapor deposition method that allows precise control over the structural and compositional properties of the sensing layer. This hierarchical architecture was further enhanced by Pd decoration, which facilitated catalytic reactions critical for H_2_ detection. The performance of the sensor was evaluated across a range of H_2_ concentrations (i.e., 2–500 ppm) and temperatures (i.e., 30–500 °C). The sensor exhibited a remarkable LOD of 2 ppm, with a rapid response time of 7 s and recovery time of 13 s. The study highlighted that the high sensitivity of sensor and selectivity stemmed from the synergistic effects of the Pd layer and the SiC nanostructure, which provided abundant active sites for gas adsorption and enhanced catalytic reactions. The dissociation of hydrogen molecules on the Pd surface, followed by the formation of water molecules through interaction with adsorbed oxygen on the SiC surface, formed the basis of the detection mechanism of this sensor design. This process generated oxygen vacancies and free charge carriers, resulting in increased conductivity. The hierarchical design of the sensing material also ensured efficient gas diffusion and interaction with active sites, contributing to its fast and reliable performance. Furthermore, the sensor demonstrated long-term stability over a period of 120 days and maintained consistent performance even under varying humidity conditions.

**Fig. 7:**
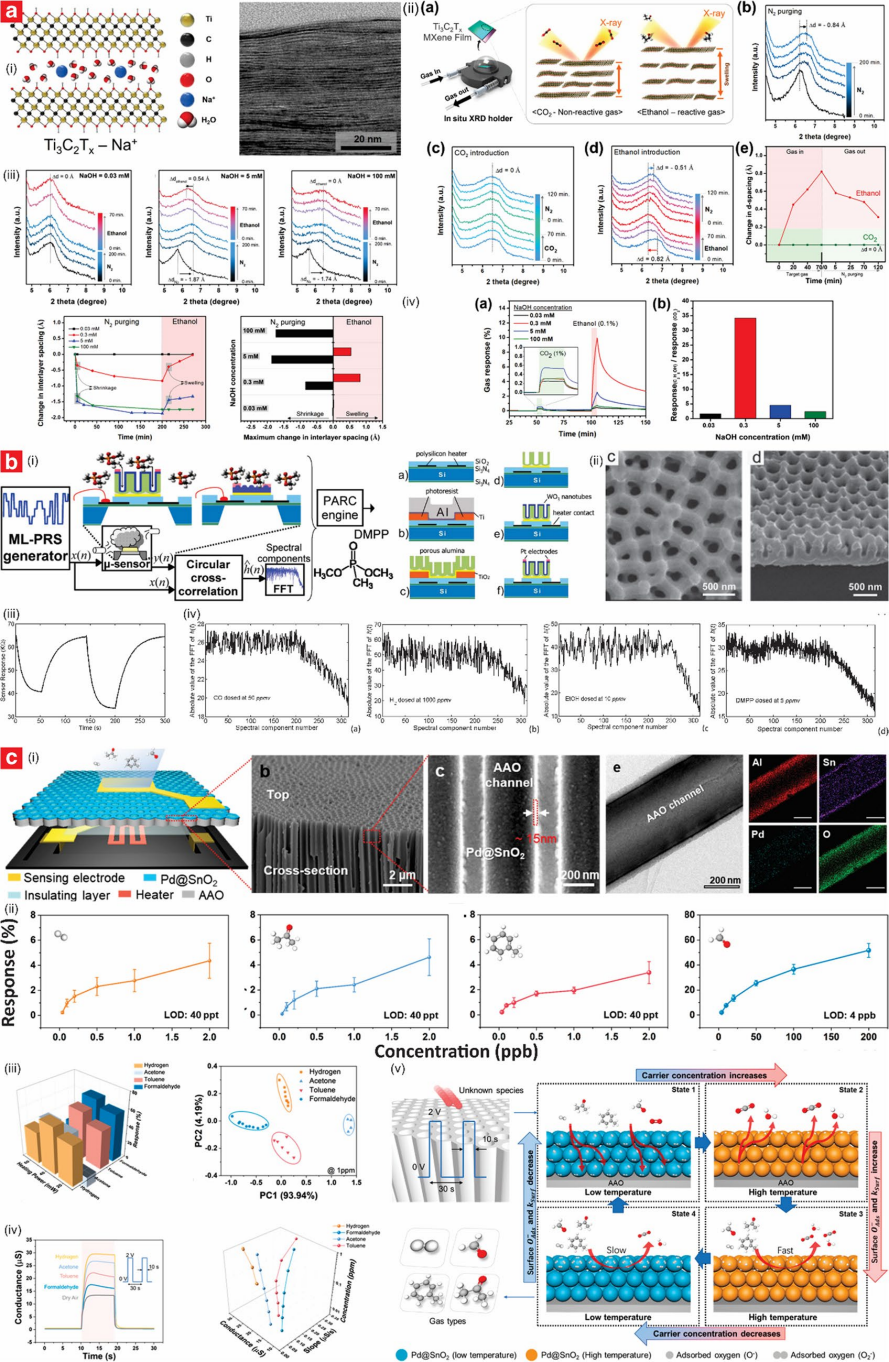
Engineering of NAA-based chemiresistive sensors functionalized with other functional materials or integrated with innovative platforms. **a** Selective ethanol detection through interlayer swelling behavior of Ti_3_C_2_T_x_ MXene gas sensors fabricated via NAA-assisted method: (i) morphological and structural characterizations of Ti_3_C_2_T_x_; (ii) sensing mechanism based on swelling behavior of Ti_3_C_2_T_x_ films and in situ XRD measurements upon gas introduction; (iii) effect of metal ion in the interlayers on the gas intercalation properties of Ti_3_C_2_T_x_ films; and (iv) ethanol selectivity analysis of the gas sensors (reproduced from ref. [165] with permission from American Chemical Society copyright 2019). **b** Engineering of WO_3_-based gas sensors with NAA templates for multifrequency measurement to enhance gas discrimination and concentration estimation: (i) sensing concept and fabrication process of NAA-supported nanotubular WO_3_ films and (ii) SEM images of the structure; (iii) response dynamic and (iv) FFT-transferred of impulse response of the sensor to various gases or vapors (reproduced from ref. [171] with permission from American Chemical Society copyright 2012). **c** Engineering of Pd@SnO2/NAA nanotube arrays on microheaters-integrated NAA for ppt-level gas detection and single-sensor gas discrimination using pulse-heating modes: (i) sensor design and structural characterization via SEM images and XPS spectra; (ii) response versus concentration curves of ultralow concentration gas sensing performance under continuous-heating mode; and trace-gas discrimination under; (iii) continuous-heating and (iv) pulse-heating modes; and (v) schematic of sensing mechanism under pulse-heating mode (reproduced from ref. [172] with permission from American Chemical Society copyright 2022)

MXene film, a lesser studied material that belong to a class of 2D transition metal carbides and nitrides, represents another promising avenue for advancing NAA-based gas sensors. Koh et al. explored the gas sensing capabilities of Ti_3_C_2_T_x_ MXene thin films functionalized through sodium ion intercalation, highlighting their high sensitivity and selectivity for ethanol vapor detection (Fig. 7a) [165]. The films were fabricated by assembling delaminated Ti_3_C_2_T_x_ MXene flakes onto NAA membranes through vacuum filtration. The preparation process involved careful control of NaOH concentration, enabling fine-tuning of the interlayer properties of the MXene films. Gas sensing measurements demonstrated that the MXene films exhibited a highly selective response to ethanol vapor compared to CO_2_ gas. The films prepared with 0.3 mM NaOH showed the highest ethanol sensitivity, with a peak response of 9.995%, while CO_2_ response remained below 1%. This chemical selectivity was attributed to the interlayer swelling mechanism, wherein ethanol molecules formed hydrogen bonds with intercalated water and surface functional groups, causing reversible expansion of the MXene layers. In contrast, CO_2_ did not induce swelling, leading to negligible sensing signals. The study further correlated the gas response to the extent of interlayer swelling observed through in situ X-ray diffraction (XRD). The MXene films with optimal NaOH treatment exhibited the largest swelling and strongest sensing response to ethanol, underscoring the importance of interlayer tuning for enhancing gas selectivity. The work demonstrated that intercalation and interlayer engineering in Ti_3_C_2_T_x_ MXene can provide a pathway for developing highly sensitive and selective gas sensors for VOCs, particularly ethanol.

Another innovative integration involved CdSe nanorods embedded into NAA templates for ethanol sensing. The CdSe nanorods, with their tunable bandgap and strong light–matter interaction, enabled visible light-driven enhancement in gas detection by generating photo-induced carriers that amplified sensitivity. Their high surface-to-volume ratio further facilitated efficient gas adsorption and rapid charge transfer, ensuring high sensitivity and fast response times. Laatar et al. introduced a novel ethanol sensor employing CdSe nanorods integrated into NAA templates, taking advantage of visible-light illumination to significantly enhance sensitivity and enable rapid gas detection [166]. The NAA template, fabricated via the two-step anodization process in 10% sulfuric acid electrolyte at 25 V and 10 °C for 45 min in each step, provided a well-ordered hexagonal pore structure with an average diameter of ~ 40 nm. CdSe nanoparticles, synthesized through a chemical route and capped with L-cysteine, were deposited into the NAA template using chemical bath deposition. Thermal annealing at 350 °C under a nitrogen atmosphere further enhanced the crystallinity of the nanorods. The CdSe nanorod-based ethanol gas sensor demonstrated exceptional sensing performance across a concentration range of 25–300 ppm, with optimal performance achieved at a working temperature of 200 °C. Visible light illumination further enhanced the sensor’s response, with the sensitivity increasing as the light power rose from 5 to 13 mW. The highest sensitivity was observed under 13 mW of light power, confirming the critical role of photo-induced electron–hole pairs in amplifying the response of the sensor. The sensitivity also exhibited a linear relationship with ethanol concentration under both dark and illuminated conditions. However, the response under visible light was significantly higher at all ethanol concentrations, highlighting the synergistic effect of light stimulation in boosting gas sensing capabilities. This enhancement was attributed to the reduction of the depletion region depth and an increase in carrier density associated with interactions between photo-generated holes and adsorbed oxygen species on the surface of the CdSe nanorods. Additionally, the sensor displayed excellent reproducibility, maintaining stable performance over five sensing cycles at 200 ppm ethanol under visible light, with the response and recovery times measured at approximately 12 and 18 s, respectively.

#### Innovative structures and integrated platforms

Innovative advancements in NAA-based chemiresistive sensors have explored novel structural designs and integrated platforms to enhance sensing performance and functionality. Special NAA structures, such as Nb_2_O_5_-based architectures fabricated through sequential anodization of Al/Nb bilayers, exemplify this creativity [167]. Mozalev et al. utilized porous 3D Nb_2_O_5_ structures formed through anodization of Al/Nb bilayers supported by NAA, where the ordered nanoporous architecture enhanced gas diffusion to the active sensing sites. The semiconducting properties of Nb_2_O_5_, including its wide bandgap and the generation of oxygen vacancies during annealing, facilitated high charge carrier mobility and increased surface reactivity. These oxygen vacancies acted as active sites for gas adsorption, creating measurable changes in conductivity, while the porous NAA-supported design ensured efficient gas-surface interactions, critical for H_2_ and ethanol sensing​ [168, 169].

MEMS-integrated platforms have further enhanced the versatility and performance of NAA-based chemiresistive sensors. By combining the unique properties of NAA with MEMS, researchers have developed compact, efficient sensors capable of precise gas detection. Mozalev et al. demonstrated this synergy with WO_3_-coated NAA on micro-hotplates fabricated via RF magnetron sputtering. The nanostructured WO_3_ leveraged the high surface area of NAA structure, facilitating faster adsorption–desorption dynamics and improving H_2_ response kinetics [170]. Further refinements through multifrequency interrogation techniques enhanced signal accuracy and response speed by optimizing the gas diffusion and processing efficiency within the WO_3_ nanotube arrays (Fig. 7b, c) [171, 172]. TiO_2_-based MEMS devices employed sol–gel deposition and physical vapor sputtering to integrate the sensitivity of TiO_2_ with the thermal stability of MEMS platforms. These systems minimized cross-sensitivity to humidity while maintaining high precision in gas detection [173, 174]. Similarly, MEMS-integrated Nb_2_O_5_ nanorod arrays exhibited robust H_2_ sensing, leveraging the wide bandgap and stable oxygen vacancies of Nb_2_O_5_ to enhance resistance modulation. The Schottky barrier at the metal–oxide interface further optimized charge carrier dynamics, amplifying the sensor performance [175]. Recent advancements include microheater-integrated platforms utilizing NAA-based designs, which significantly reduced power consumption by leveraging the thermal efficiency of NAA and eliminating heat loss through horizontal conduction. These robust designs, as shown by Lee et al., achieved operational stability under high temperatures and reduced power consumption to as low as 9 mW during pulsed operation, highlighting their suitability for low-power, high-performance gas sensing [176].

### Impedance spectroscopy–based sensors

Impedance spectroscopy (IS) has proven to be a highly effective approach for gas sensing, as it measures changes in the dielectric properties of materials resulting from the interaction between an applied electric field and the electric dipole moment of the material across various frequencies when gas molecules interact with the sensing surface. Specifically, the technique analyzes how the interaction of gas molecules with the surface of a sensing material induces variations in resistance, capacitance, or a combination of both. The integration of NAA-based sensing systems with IS provides several benefits. This non-destructive technique enables a detailed understanding of the complex physicochemical processes occurring within the material, such as charge transfer, adsorption dynamics, and ion migration. It also enables the identification of equivalent circuit models that correlate electrical responses with structural and compositional features of NAA, aiding in the systematic optimization of sensing performance. Furthermore, IS is highly sensitive to subtle changes in gas concentration, making it a valuable tool for detecting low concentrations of analytes, with high precision. Some representative work in this field are summarized in Table 4.

**Table 4:** Summary of NAA-based impedance spectroscopy sensors

Sensor design/chemical modification*	Detected gases**	Sensing mechanism	Ref
Alteration of pore size and length via anodization voltage and time Implementing IS to distinguishes between contributions from the membrane’s intrinsic electrical properties and electrical components	Water vapor (-)	Water vapor adsorption into the nanopores alters the dielectric constant of the alumina, leading to changes in capacitance	[179]
Pt nanowires formation within the NAA through electrodeposition	O_2_, H_2_, N_2_ (-)	Gas adsorption/desorption on Pt nanowires modulates resistance via catalytic charge transfer interactions between Pt and gas molecules at varying temperatures	[180]
NPt deposited via thermal evaporation Detection of gas species in aqueous phase Implementation of IS for validation of electrocatalytic efficiency of NPt and characterizing enhanced electron transfer	O_2_, NO, HNO in water and PBS buffer solution (-)	Gas adsorption on Pt nanowires modulates resistance via catalytic charge transfer interactions, influenced by pH and redox behavior	[181]
Ppy coatings applied to NAA substrates via dip-coating As-prepared Ppy/NAA sheet was adhered onto the Ag-Pd interdigitated electrode pattern	NH_3_ (5–95 ppm)	NH_3_ adsorption leads to changes in impedance due to interfacial polarization and charge redistribution within Ppy matrix	[182]
Room-temperature sensor based on magnetically aligned NiNWs grown on NAA template NiNWs grown via electrochemical deposition Ag interdigitated electrodes fabricated via sputtering using shadow mask	Ethanol (2–18 ppm) Humidity (30, 50, and 70%RH)	Adsorption of ethanol and water vapor on aligned Ni nanowires increases resistance, attributed to electron scattering and barrier formation at nanowire junctions	[183]

*All sensors summarized in this table use straight-pore NAA structures unless otherwise stated

**Reported concentration ranges correspond to the tested working range of the sensors

The high surface-to-volume ratio and intrinsic dielectric properties of bare NAA structures have been exploited to study the detection capability of the platform for different gases. Studies on unmodified NAA demonstrate its efficacy in detecting gases such as NH_3_ and cyclic VOCs, with IS enabling the evaluation of pore size-dependent adsorption behavior and ion migration mechanisms [177, 178]. Abrego et al. investigated the effects of the anodization parameters, such as time and voltage, on pore size distribution and density of NAA platforms, demonstrating the critical role of these structural features in determining sensitivity and selectivity using an IS-based transduction mechanism [179]. In their study, the NAA structures were fabricated through the two-step anodization process in 0.4 M sulfuric acid at 0 °C under varying conditions of voltage (16–31 V) and time (60–300 min). This process resulted in a self-ordered hexagonal pore arrangement with an average pore diameter of 20–29 nm, a pore density ranging from 10^11^ cm^–2^ at lower voltages to 10^10^ cm^–2^ at higher voltages, and a pore spacing of approximately 45 nm. The as-produced NAA film was directly used for gas sensing experiments, relying solely on its intrinsic properties, with electrical contacts established using silver paint on the NAA layer and the pure aluminum backside. IS was employed to monitor the sensing response in a controlled environment. The study demonstrated that the NAA-based sensor could detect water vapor through changes in relative capacitance (*ΔC/C*) across a sensing concentration range from 20 to 100 μL of water vapor. Sensitivity increased with both pore diameter and film thickness, which were readily controlled by the anodization voltage and time. The detection mechanism was further characterized through frequency-dependent impedance measurements. At low frequencies, the response was dominated by the electrical contact properties, as indicated by a semicircular feature in the impedance spectrum’s Cole–Cole plot, corresponding to contact resistance, Warburg impedance, and double-layer capacitance. However, at high frequencies, the response reflected the intrinsic electrical properties of the NAA membrane, evidenced by a second semicircular arc in the Cole–Cole plot. This high-frequency behavior was modeled in the equivalent circuit using membrane resistance and capacitance, revealing the contribution of the NAA structure’s dielectric and the pore-related properties to the sensor’s electrical characteristics. These early studies established a foundational understanding of combining NAA films with IS for gas sensing applications, with the integration of IS enabling detailed modeling of electrical properties, such as resistance and capacitance, and providing direct insights into gas molecules–surface interactions within the porous network and guiding the design of NAA sensors.

While bare NAA structures serve as robust, scalable substrates for gas sensing, their performance is inherently constrained by limited gas specificity and sensitivity. Integrating functional materials such as noble metals, polymers, or composites into the nanoporous architecture of NAA enhances their capabilities in IS-based sensing systems [180–182]. This integration leverages the synergistic effects of IS and functionalized NAA platforms, offering improved gas sensing performance with enhanced specificity and ability to characterize gas interactions. For example, Vunnam et al. presented Pt-functionalized NAA structures engineered via electropolymerization, resulting in a highly conductive and catalytically active system for O_2_ sensing [180]. The NAA structure was fabricated through a single-step anodization of aluminum sputtered onto silicon wafers, performed in oxalic acid at 40 V at room temperature. Pt was deposited into the pores via electropolymerization, forming uniformly distributed and aligned nanowires that conform to the NAA nanopore matrix. To complete the sensor structure, gold electrodes were then deposited via evaporation to establish electrical contacts for IS measurement. The use of IS technology with the functionalized NAA platform enabled real-time, frequency-dependent analysis of resistance and capacitance changes in response to gas adsorption and desorption. The fabricated sensor demonstrated distinct impedance behaviors for different gases, highlighting the potential of IS to differentiate between O_2_, H_2_, and N_2_ based on their unique interactions with the Pt nanowires. The sensitivity to pure O_2_, reflected by a resistance change (*ΔR/R*) of up to 0.2 at 40 °C, showcased the ability of the sensor to detect specific gases with high precision. Similarly, the decrease in resistance observed with H_2_ and N_2_ (*ΔR/R* of –0.045 and –0.12, respectively) underlined the robustness of the IS-based approach in characterizing gas interactions. By leveraging the advantages of IS, this study demonstrated the transformative potential of combining this analytical technique with functionalized NAA-based platforms. The ability to detect and differentiate gases through impedance analysis enhanced the specificity and reliability of the sensor, making it a promising candidate for advanced gas sensing applications.

**Fig. 8:**
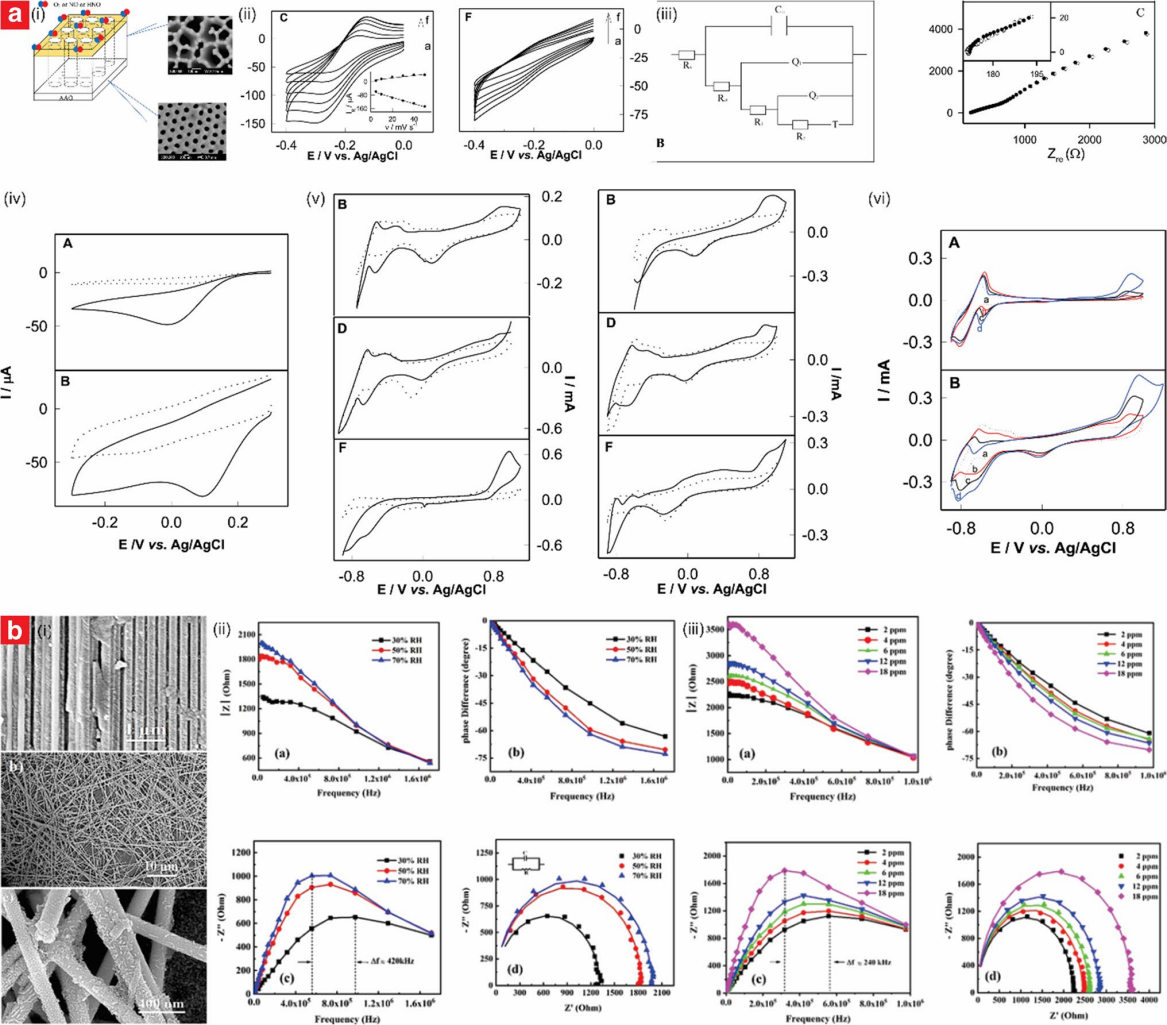
Engineering of NAA-based gas sensors utilizing impedance spectroscopy. **a** Engineering nanoporous Pt electrodes on NAA templates, utilizing impedance spectroscopy to enhance electrocatalytic activity toward reactive oxygen and nitrogen species across varying pH conditions: (i) schematic of the design and SEM images of Pt/NAA sensor; (ii) CV response, (iii) equivalent circuit and representative Nyquist plot of the nanoporous NPt sensor; (iv) CV curves of nanofilm-Pt (A) and NPt (B) during exposure to saturated O_2_; (v) and (vi) CV curves of NPt during exposure to various gas-absorbed liquids (reproduced from ref. [181] with permission from Elsevier copyright 2018). **b** Engineering of NAA-based sensors with aligned NiNWs and impedance spectroscopy to achieve room-temperature selective ethanol and humidity sensing: (i) SEM images of NiNWs inside and outside of NAA template; Bode and Nyquist plots of the sensor during exposure to different concentrations of (ii) RH and (iii) ethanol (reproduced from ref. [183] with permission from IOP Publishing copyright 2020)

Similarly, Tao et al. introduced a novel nanoporous platinum (NPt) electrode, prepared on a NAA membrane via thermal evaporation, and combined it with IS for the real-time electrocatalytic detection of reactive oxygen and nitrogen species, including O_2_, NO, and nitroxyl (HNO) from Angeli’s salt (Fig. 8a) [181]. The NAA membranes, commercially obtained with pore sizes of 100 nm and 200 nm, were used as templates for NPt deposition. Pt was deposited via thermal evaporation at a base vacuum of ~ 10^–7^ Torr, resulting in a thin film of ~ 40-nm thickness. The resulting electrode exhibited “nanocavities” formed by interconnected Pt nanoparticles, providing a significantly enhanced surface area compared to conventional nanofilm-Pt electrodes. Electrochemical IS and cyclic voltammetry (CV) demonstrated the advantages of the nanoporous structure, including higher conductivity and larger electroactive surface areas, particularly in the NPt-200 electrode, where its 200-nm pores enabled more efficient electron transfer and facilitated gas interactions. For instance, the NPt-200 electrode demonstrated a catalytic sensitivity of 0.85 mA⋅cm^–2^ to O_2_, compared to 0.54 mA⋅cm^–2^ for the nanofilm-Pt. Similarly, the catalytic currents for NO and HNO were approximately ten and three times larger, respectively, on the NPt-200 electrode compared to the nanofilm-Pt. These enhancements were attributed to the significantly lower impedance (i.e., 1.24–1.48 Ω⋅cm^2^) of the NPt electrodes compared to that of the reference nanofilm-Pt electrodes, enabling efficient charge transfer and promoting redox reactions at the interface. In addition, the semicircular features of Nyquist plots highlighted the improved conductivity of the NPt structures compared to that of planar electrodes, underscoring the role of IS in elucidating the enhanced charge transport in these systems.

In another study, Zia and Ali Shah investigated a polypyrrole-based nanocomposite integrated with NAA, which exhibited enhanced sensitivity to NH_3_ due to the synergistic benefits of combining functional polymer materials with nanoporous structures, and a dynamic impedance response to gas adsorption [182]. The NAA structure, with pore diameters of 30–40 nm, was fabricated by anodizing aluminum foils at 10 V in a 10% v/v sulfuric acid solution, followed by post-anodization treatments such as pore widening in phosphoric acid and annealing at 300 °C. Polypyrrole (Ppy) was synthesized via chemical oxidation polymerization using hydrogen peroxide as an oxidizing agent, then deposited onto the NAA substrate through dip-coating. Repeated coatings formed a robust layer of Ppy, which was heat-treated at 100 °C for 2 h to ensure adhesion and stability. The composite device was assembled on a ceramic substrate featuring Ag–Pd interdigitated electrodes, forming the final sensor configuration. IS was employed to monitor the response of the composite sensor to NH_3_ concentrations ranging from 5 to 95 ppm. The impedance response was most pronounced at low frequencies, particularly at 100 Hz, due to interfacial polarization effects governed by the Maxwell–Wagner–Sillars mechanism. The results demonstrated high sensitivity at lower concentrations, with a response of approximately 1.74 × 10^6^ Ω⋅ppm^–1^ at 100 Hz, decreasing to 6.29 × 10^4^ Ω⋅ppm^–1^ at higher concentrations. The dynamic response and recovery times were notably fast at ~ 6 s, highlighting rapid adsorption–desorption dynamics. Furthermore, the sensor exhibited excellent stability over two months, with resistance changes remaining consistent during bi-weekly measurements under ambient conditions.

Hybrid platform gas sensors combining NAA with IS offer a powerful approach to enhancing gas detection performance. By integrating functional materials and leveraging the ability of IS to provide frequency-dependent analysis, these sensors achieve improved sensitivity, selectivity, and a deeper understanding of gas–material interaction mechanisms, making them highly versatile for various gas sensing applications. For instance, Radzik et al. introduced a hybrid thick-thin NAA structure for methanol sensing, utilizing IS to probe the influence of material heterogeneity on impedance response [95]. The thick-thin NAA structure (pore diameter of ~ 40 nm) comprised of two layers: a thin layer of NAA film, and a thick layer of interdigitated capacitor electrodes on top of the NAA surface. The NAA thin film was fabricated through the anodization (in 4% oxalic acid at 40 V and room temperature) of aluminum thin films (1 µm thick) deposited on alumina substrates. After anodization, an interdigitated capacitor was then patterned on the surface of the NAA using a screen-printed conductor and fired on a belt-furnace. Using IS, the authors investigated the sensor’s frequency-dependent response to varying methanol concentrations. The analysis revealed distinct changes in capacitance and resistance, with the sensor exhibiting maximum sensitivity at lower frequencies (< 2 kHz). The capacitance demonstrated an exponential relationship with methanol concentration, providing a quantifiable signal that reflected changes in the dielectric environment within the nanopores. The authors also studied the ability of the sensor to distinguish methanol from water vapors, with Nyquist and Cole–Cole plots revealing unique response patterns for each vapor. For methanol, the Nyquist plots display pronounced shifts in both resistance (real component) and reactance (imaginary component) with increasing concentration, forming distinct semicircular patterns that scaled with methanol dilution. In contrast, water vapors caused only minor changes in resistance and capacitance, with smaller shifts observed in the semicircles of the Nyquist plots. These differences reflected methanol’s stronger adsorption and dielectric effects on the NAA structure compared to water, which had limited interaction with the sensor material.

Similarly, Mohammadi et al. integrated NAA structures with aligned nickel nanowires (Ni NWs) to create a room-temperature gas sensor, employing IS to analyze the interaction of ethanol and humidity with the nanowire system (Fig. 8b) [183]. The NAA substrate, with an average pore diameter of ~ 145 nm, was fabricated through hard anodization in 0.3 M oxalic acid, with voltage ramping from 40 to 130 V over 60 min, followed by post-anodization treatments including pore widening and removal of the BOL. Ni NWs were synthesized within the NAA substrate via electrochemical deposition, using an electrolyte containing nickel sulfate and boric acid, and aligned on silver interdigitated electrodes. Magnetic alignment of the ferromagnetic NiNWs and subsequent annealing at 100 °C ensured strong interfacial contacts and uniform orientation. IS revealed the sensor’s ability to detect ethanol vapor in the concentration range of 2–18 ppm, with a response ratio (*R*_*air*_*/R*_*ethanol*_) of 2.72 observed at 18 ppm and 50% RH. The sensor also demonstrated sensitivity to changes in humidity (30%–70% RH), although ethanol caused more pronounced impedance shifts. Nyquist and Bode plots highlighted significant resistance increases upon ethanol exposure, particularly at low frequencies, attributed to enhanced charge transfer resistance at the nanowire junctions and interfaces. This was apparent from the larger semicircles in the Nyquist plots and increased phase angles in the Bode plots, which denoted stronger ethanol adsorption and slower charge transfer dynamics at higher concentrations. Capacitance changes were also monitored; however, they were minimal, likely due to substrate noise and limited interfacial effects within the studied frequency range.

## Optical sensors

Solid-state optical gas sensors represent an advanced class of devices designed to detect and quantify gas molecules through changes in their optical properties. These sensors operate on the principle of light–matter interactions, where gas molecules modify the way in which a platform material interacts with light, altering its properties such as fluorescence intensity, plasmonic resonance, or refractive index. Compared to other types of sensors, optical gas sensors are non-invasive, capable of miniaturization, and less susceptible to environmental interferences, making them suitable for a wide range of applications, from environmental monitoring to medical diagnostics [4, 184, 185].

Nanostructured materials play a crucial role in enhancing the performance of optical gas sensors, not only because of their high surface-area-to-volume ratio but also because of their ability to enable the formation of unique optical structures to interact with electromagnetic waves. NAA is particularly noteworthy among nanostructured materials for developing optical gas sensors that are compact, reliable, and versatile, addressing critical challenges in modern sensing applications. NAA serves a dual function as both a sensing matrix, providing sites for gas adsorption, and an optical transduction platform, converting gas-induced changes into measurable signals. The unique optical properties of NAA are intrinsically linked to its nanostructured architecture, which is achieved through the anodization. Modifying anodization conditions allows for the creation of unique optical structures and tailored light propagation properties, enhancing the sensitivity of the sensor to gas-induced changes of physical properties. The porous structure of NAA can be functionalized with specific molecules, enabling both enhanced sensitivity and specificity toward analyte gases and the creation of advanced optical systems. For example, plasmonic structures can be realized by combining NAA-based dielectric mirrors with metallic coatings, while embedding light-emitting materials, such as fluorescent dyes or quantum dots, allows for the design of luminescent sensors. Such functionalization approach significantly enhances the capabilities of NAA-based optical sensors by introducing novel sensing mechanisms and improving their overall performance across diverse applications.

As the field progresses, the integration of NAA structures into compact and multi-functional optical platforms continues to offer practical solutions for real-world gas sensing challenges. Innovations focus on embedding NAA within photonic circuits, enhancing signal processing capabilities while maintaining sensor miniaturization. The porous architecture of NAA also facilitates hybrid designs, where optical sensing mechanisms are combined with complementary detection techniques, enabling multi-modal analysis for improved sensitivity and selectivity​​. Table 5 summarizes the most representative advancements in optical gas sensors based on NAA structures, which will be described in detail in the following sections, highlighting the sensing mechanisms, target gases, and fundamentals, providing an overview of the state-of-the-art developments in this field.

**Table 5:** Summary of NAA-based optical sensors

naa platform		Detected gases*	Sensing mechanism	Ref
NAA structure	Sensor design/chemical modification			
Refractive index-based sensors—interferometric sensors				
Bilayered straight pore (NAA–BFPIs)	Bi-layers having different pore diameters Thin Au film deposited on top via sputtering to enhance reflectometric interference RIfS used to analyze changes in *OT*_*eff*_ Integrated with microfluidic setup for gas sensing assessment experiments	Ethanol (0, 56, 112 g⋅m^–3^)	Ethanol infiltrates the NAA-BFPI layers, altering the refractive index of the layers and causing measurable change in *ΔOT*_*eff*_, which is detected through Fabry–Pérot interference fringe shifts; the system enables layer-specific sensitivity	[190]
Straight pores	Room-temperature sensor based on reflective interferometric design Thin Au film deposited on top via sputtering to enhance reflectometric interference RIfS used to analyze changes in *OT*_*eff*_ Integrated with microfluidic setup for gas sensing assessment experiments	H_2_S (0–2% in air)	H_2_S selectively binds onto the Au-coated surface of NAA, altering the refractive index of the porous layer and shifting Fabry–Pérot interference fringes	[191]
Straight pore	Room-temperature sensor based on reflective interferometric design Au or Pt thin film deposited on top via sputtering for specific gas affinities RIfS used to analyze changes in OT_eff_ Integrated with microfluidic setup for gas sensing assessment experiments	H_2_S (0–2% in air) H_2_ (~ 2%)	H_2_S or H_2_ adsorb on metal-coated surface of NAA (e.g., Au or Pt), altering the refractive index of the porous layer and shifting optical fringes via gas-specific metal–surface interactions (e.g., Au enables H_2_S detection via Au–S interaction, and Pt enables H_2_ detection via lattice expansion)	[192]
Refractive index-based sensors—photonic crystal sensors				
Quasi 1D–PC	Uniform pore structure with alternating layers of stem (290 nm thick) and branched (140 nm thick) channels	Ethanol (0–13.72 mmol/L)	Adsorption of ethanol gas into the porous structure alters the effective refractive index of the stem and branched channel layers, causing the red shift in PSB position	[196]
1D–PC with microcavity (NAA–μCVs)	PC featuring optical microcavity fabricated using SPA for label-free sensing of VOCs Tunable RBs in the visible spectrum (450, 550, 650 nm) Transmission spectroscopy used to analyze shifts in the RB position	Ethanol (11.2–55.8 g⋅m^–3^) Isopropanol (7.7–38.4 g⋅m^–3^) Acetone (58.9–294.4 g⋅m^–3^)	VOCs adsorb into NAA–μCV nanopores, altering the refractive index and shifting the RB in the transmission spectrum; each VOC has a distinct adsorption/desorption kinetic profile, which act as identification fingerprints	[197]
1D–PC with Fabry–Pérot microcavity (NAA–HOμCV)	PC featuring extended length optical microcavity fabricated using SPA for label-free discrimination of VOCs Tunable high-order RBs in the visible spectrum acting as optical fingerprint upon exposure to gases	Ethanol (11.2–55.8 g⋅m^–3^) Isopropanol (7.7–38.4 g⋅m^–3^) Acetone (58.9–294.4 g⋅m^–3^)	VOCs adsorb into NAA-HOμCV nanopores, altering the refractive index and shifting multiple high-order RBs independently; real-time detection and discrimination of VOCs based on high-order RBs fingerprint presented in radar map format	[198]
Tamm plasmon resonance structures based on NAA–GIF	NAA–GIF fabricated using SPA Tamm plasmon structure created by deposition of noble metals thin film (Au, Ag, and Al) on NAA–GIF via sputter coating and thermal evaporation	MPTMS (0–30 ppm in ethanol vapor)	Specific binding of thiol groups from MPTMS molecules to the metal surface alters the local refractive index, causing a red shift in Tamm plasmon resonance	[199]
Colorimetric sensors				
Straight pore	Immobilization of BPB and BCG triphenyl-methane type indicators via adsorption on NAA with dodecylbenzenesulfonic acid as a catalyst and post-functionalization protonation with a sulfonic acid solution Optical reflection measurements conducted using LEDs and photodiodes	NH_3_ (0–100 ppm)	NH_3_ deprotonates immobilized acid–base indicators via a reversible pH-dependent protolytic reaction, resulting in a color change due to altered absorbance at specific wavelengths	[201]
Straight pore	Spiropyran molecules grafted onto NAA templates via covalent bonding with APTES-functionalized NAA	Volatile acids (acetic acid, formic acid, nitric acid, hydrochloric acid)	Volatile acid vapors protonate merocyanine on NAA, causing a shift in absorption peaks and a visible color shift, which is reversible and tunable via UV/visible light exposure	[202]
Plasmonic effect-based sensors				
Straight pore	Functionalization with ZnO nanoparticles synthesized by the sol–gel method via spin coating on NAA chip Ni and Au layers deposited onto the NAA by e-beam evaporation to enhance LSPR Reflectance optical probe used to detect LSPR-induced changes in reflected light	Ethanol (0–100 ppb)	Ethanol adsorbs onto ZnO nanoparticles deposited on Au-coated NAA, altering the local refractive index and shifting the LSPR condition, which is detected as a change in reflected light intensity and wavelength	[203]
Straight pore	Hexagonally ordered thin film of Au nanoparticles deposited onto nanobowled NAA Integrated with SiO_2_ pillars for enhanced sensitivity	He, Ar (2 × 10^4^ Pa) CO_2_ (10–20% in Ar)	Gas molecules adsorbed onto the surface of gold nanoparticles modulate the local refractive index, causing measurable shifts in the LSPR wavelength	[204]
Straight pore	Au nanoparticles deposited on NAA via sputtering to create plasmonic platform for SERS sensing SERS used for label-free, single-virus-level detection and enrichment of airborne viruses NAA also acts as size-selective filtration	SARS-CoV-2 pseudoviruses (-)	Viruses enrich in Au–NAA nanopores and are detected via SERS using plasmon-enhanced molecular fingerprinting	[205]
Straight pore	The alumina nanowires created from chemical etching of NAA template Ag deposited onto the nanowires using ion-beam sputtering or thermal evaporation SAM of 1-propanethiol functionalization for benzene gas adsorption	Benzene (< 0.1 ppm–10 ppm)	Benzene adsorbs on thiol-functionalized Ag nanowires, amplifying Raman signal due to enhanced electromagnetic fields via surface plasmon resonance hotspots	[206]
Luminescence sensors				
Straight pore	Functionalization of pyrene-1-butylic acid to emit fluorescence, with myristic acid was co-adsorbed to for controlling excimer formation and preventing aggregation	O_2_ (0–100%)	O_2_ quenches PBA excimer fluorescence via dynamic (oxygen collides with excited PBA molecules) and static (oxygen forms ground-state complexes) non-radiative energy transfer	[208]
Straight pore	Pt-based dyes were embedded in a sol–gel matrix and spin-coated onto NAA templates to create a uniform sensing layer	O_2_ (0–100%)	O_2_ quenches the phosphorescence of PtTFPP and PtOEP dyes phosphorescence via Dexter energy transfer, following dynamic and static models	[210]

*Reported concentration ranges correspond to the tested working range of the sensors

### Refractive index–based sensors

Refractive index-based optical sensors exploit the changes in the effective refractive index of NAA platforms induced by gas adsorption onto the pore walls or infiltration into the nanopores, which alter the local dielectric environment. This interaction modifies the way light propagates through or is reflected from the NAA structure, causing measurable shifts in reflectance, transmittance, or interference fringes [186, 187]. These shifts form the basis for precise gas detection, making refractive index-based sensing a simple yet powerful approach for gas sensing applications. The sensitivity of this detection mechanism is determined by the interplay between the gas’s concentration and its molecular characteristics, such as polarizability, its affinity with the surface of the material as well as the structural attributes of the NAA platform. By tailoring parameters such as pore size, porosity, and surface properties, NAA structures can be finely engineered to enhance detection performance, ensuring accurate and efficient sensing capabilities.

#### Interferometric sensors

NAA-based interferometric sensors operate on the principle of light interference to monitor changes in the effective optical thickness (*OT*_*eff*_) of NAA films. Gases that interact with the nanopores alter the optical path length of the structure by changing the effective refractive index, which in turn induces spectral shifts in the Fabry–Pérot interference fringes. These spectral changes provide insights into gas adsorption, condensation, or desorption within the nanoporous network. The dynamics of capillary condensation in NAA, one of the sensing mechanisms in nanoporous structure, were explored in the early studies of Casanova and co-workers [188, 189]. In these studies, simple bare NAA structures featuring straight cylindrical nanopores were used to investigate the interaction of organic vapors (i.e., isopropanol and toluene) with the pore surfaces. Optical interferometry was employed to monitor adsorption and desorption cycles, revealing that evaporation occurs at equilibrium pressures predicted by the classical Kelvin equation, even at nanoscale dimensions. In contrast, condensation exhibited hysteresis, which was attributed to metastable vapor states, surface defects, and fluid–solid interactions mediated by van der Waals forces. Furthermore, these studies highlighted that the geometrical parameters of NAA, particularly pore size and uniformity, play a critical role in determining adsorption behavior and the reproducibility of gas detection, providing a framework for optimizing NAA as a platform for vapor sensing.

**Fig. 9:**
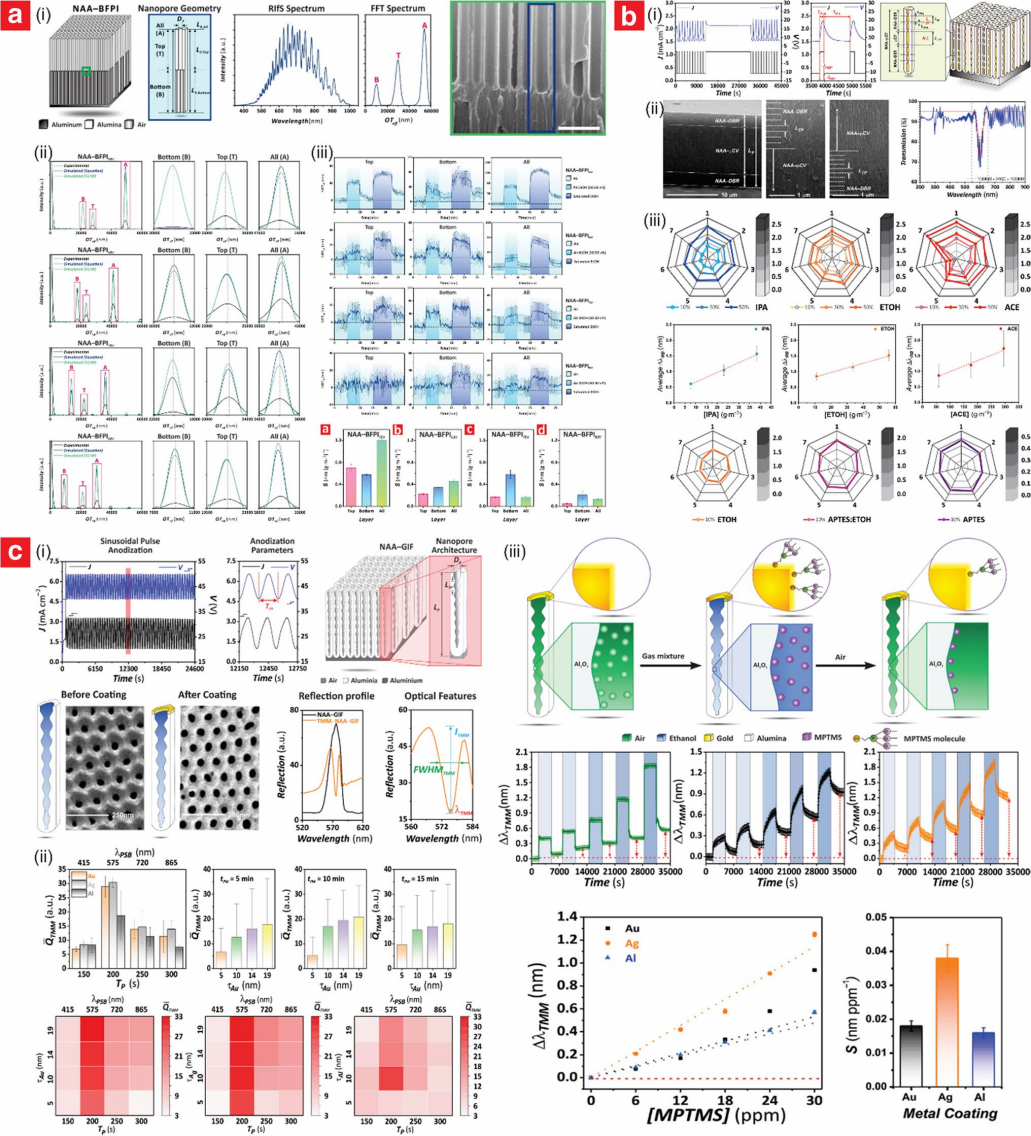
Engineering of NAA-based optical gas sensors based on refractive index change transduction method. **a** Engineering bilayered NAA Fabry–Perot interferometers via anodization and chemical etching to achieve real-time gas sensing using RIfS: (i) engineering of NAA − BFPIs, and optical and structural characterizations; (ii) experimental and simulated FFT spectra of various representative NAA − BFPIs; and (iii) real-time monitoring of Δ*OT*_*eff*_ of NAA − BFPIs upon exposure to ethanol vapor and sensitivity bar chart analysis (reproduced from ref. [190] with permission from American Chemical Society copyright 2023). **b** Engineering NAA optical microcavity (NAA-HOμCVs) with high-order resonance modes for discriminative gas detection of VOCs using spectral shift analysis: (i) structural engineering via galvanostatic pulse anodization; (ii) structural and optical characterizations; and (iii) discriminative gas-sensing performance assessments (reproduced from ref. [198] with permission from American Chemical Society copyright 2024). **c** Engineering Tamm plasmon resonances in NAA–PC for enhanced light confinement and gas sensing sensitivity: (i) fabrication of dielectric component (NAA–GIF) via SPA; (ii) engineering of TMM band via modifications of plasmonic components (i.e., metal coatings); and (iii) assessment of the chemical sensitivity of TMM − NAA − GIFs (reproduced from ref. [199] with permission from American Chemical Society copyright 2024)

Lim et al. expanded on these principles by developing bi-layered NAA Fabry–Pérot interferometers (NAA–BFPIs) as optical sensing platforms for ethanol vapor detection (Fig. 9a) [190]. The innovative architecture of NAA–BFPIs fabricated using a three-step anodization process in 0.3 M oxalic acid at 6 °C consisted of two distinct layers: a top layer with larger nanopores and a bottom layer featuring smaller nanopores. Precise engineering of these structures was achieved through varying anodization voltages (i.e., 50–80 V for the top layer and 29–46 V for the bottom layer), anodization time, and pore widening treatment. This stratified structure allowed the sensor to exhibit layer-specific optical responses due to its distinct sensitivities, where the top layer with larger nanopores demonstrated higher sensitivity to liquid ethanol, while the bottom layer with smaller nanopores achieved maximum sensitivity to ethanol gas. The sensing mechanism used changes in effective optical thickness (*ΔOT*_*eff*_) caused by ethanol gas adsorption within the nanopores as the core sensing principle. Measurements performed using reflectometric interference spectroscopy (RIfS) demonstrated distinct and reproducible optical responses. The study reported a maximum sensitivity towards ethanol vapor of 1.21 ± 0.01 nm⋅(g⋅m^–3^)^–1^ for the bilayer configuration in NAA–BFPI_50 V_, demonstrating the highest detection performance with excellent linearity (*R*^2^ = 0.9997). Sensitivity values for individual layers were also detailed: the top layer exhibited a sensitivity of 0.70 ± 0.06 nm⋅(g⋅m^–3^)^–1^ (*R*^2^ = 0.9752), while the bottom layer showed a slightly lower sensitivity of 0.58 ± 0.02 nm⋅(g⋅m^–3^)^–1^ but with superior linearity (*R*^2^ = 0.9975). These findings highlighted the contributions of both layers to the overall sensing mechanism and the ability of the bilayered structure to provide enhanced and versatile detection capabilities for ethanol gas sensing. Real-time monitoring confirmed the stability and repeatability of the sensing system, with negligible performance variation across measurement cycles.

In biomedical diagnostics, NAA platforms have been combined with RIfS technique to detect H_2_S—a key volatile sulfur compounds (VSCs) in human breath associated with oral malodor [191, 192]. Kumeria et al. developed an NAA-based reflective interferometric sensor for detecting H_2_S in human breath, featuring a gold-coated porous structure that enhanced selectivity and sensitivity through strong affinity for S-containing compounds [191]. The NAA platform was fabricated through the two-step anodization process in 0.3 M oxalic acid at 0 °C, resulting in a highly ordered porous structure with pore diameters of 30–35 nm and a pore length of approximately 4 µm. To enhance sensitivity and selectivity toward S-containing compounds, the NAA surface was coated with an 8-nm Au layer via vapor deposition to attain strong selectivity through the affinity of Au for thiol (–SH) groups in S-containing compounds. The sensor demonstrated concentration-dependent wavelength shifts of the Fabry–Perot interference fringes, with shifts ranging from 1 to 14 nm for H_2_S concentrations between 0 and 2% in air. The corresponding *OT*_*eff*_ changes were directly linked to the effective refractive index alteration in the film caused by the adsorption of H_2_S molecules on the gold-coated surface. The sensor exhibited high specificity for S-containing compounds, as no significant interference was observed when exposed to other gases such as H_2_ or air. The real-time performance of the sensor was validated for oral malodor analysis in volunteers. The sensor successfully detected VCSs, primarily H_2_S, at typical concentrations of 0.2–0.4 µg L^–1^ found in healthy individuals. The ability to monitor changes in *OT*_*eff*_ signals in response to breath samples confirmed its practical application for distinguishing oral hygiene conditions based on VSC levels. In another study from the same author, they expanded the scope of RIfS by using both Au and Pt coatings on NAA sensors to detect H_2_S and H_2_ gases [192]. To enable gas-specific detection, the NAA surface was coated with an 8 nm layer of either Au or Pt to detect H_2_S and H_2_, respectively. The Au-coated NAA platform exhibited high sensitivity to H_2_S, effectively detecting concentrations typical of oral malodor (i.e., ~ 0.2–0.4 µg L^–1^) and demonstrating wavelength shifts up to 14 nm at 2%, with recovery times significantly reduced from 50 to 60 min at room temperature, which could be reduced to 10 min with moderate heating (60–65 °C). Pt-coated NAA selectively detected H_2_ through *OT*_*eff*_ shifts caused by lattice expansion at concentrations up to 2%, while showing no response to air.

#### Photonic crystal sensors

Photonic crystal (PC) sensors exploit the periodic modulation of the effective refractive index within NAA structures and the characteristic photonic stopband (PSB) of these photonic systems—specific wavelength region where light propagation is prohibited. Interaction with gases causes measurable shifts in the PSB, enabling highly sensitive and selective gas detection and making it a powerful approach for optical sensing. NAA–PC structures are created through precise pulse anodization techniques [18]. These methods produce alternating layers of varying effective refractive indices by controlling pore geometry and spacing, resulting in configurations such as distributed Bragg reflectors (DBRs), gradient index filter (GIFs), or microcavities [44, 193–195]. The inherent tunability of these structures makes NAA–PC sensors highly adaptable for addressing diverse gas sensing challenges. Shang et al. introduced a narrow-bandwidth NAA–PC sensor fabricated using step-voltage compensation anodization [196]. This approach precisely controlled the pore geometry and layer thickness by applying a voltage waveform with sinusoidal increases and linear decreases during each anodization period, resulting in a 1D NAA–PC in the form of a DBR with a PSB that had a full width at half maximum (FWHM) of 30 nm. The sensor demonstrated significant sensitivity and excellent repeatability to ethanol vapor, with a total red shift of the PSB of 66 nm as the ethanol concentration increased from 0 to 13.72 mmol⋅L^–1^ (saturated). This sensing was driven by the changes in the effective refractive index of the PC platform produced by the adsorption of ethanol molecules into the nanopores. The sensitivity was further enhanced by locating the PSB in the near-infrared (NIR) range, as the longer wavelength facilitated larger shifts in response to analyte adsorption.

Microcavity-based photonic crystals exploit light confinement within defective layers embedded between two dielectric mirrors (e.g., DBRs or GIFs) to create a narrow resonance band (RB), which is highly sensitive to environmental refractive index changes. The structural and optical characteristics of these microcavities are tailored through advanced anodization techniques, enabling precise gas detection and, notably, gas discrimination based on unique spectral signatures. Tran et al. developed NAA-based optical microcavities (NAA–μCVs) using sinusoidal pulse anodization (SPA) to create a light-confining structure with tunable RBs for real-time, selective gas sensing of VOCs [197]. The NAA–μCVs were fabricated using sinusoidal pulse anodization (SPA) in 1 M sulfuric acid at –2 °C, resulting in a highly ordered structure with a central physical cavity layer sandwiched between two NAA–GIF dielectric mirrors. The precision of the fabrication process yielded pore diameters of 7–9 nm and a total thickness of 22–24 μm, with RBs engineered at 450, 550, and 650 nm to optimize optical performance. These RBs exhibited an average normalized quality factor (*Q̅*) of approximately 40, signifying sharp and narrow spectral features ideal for gas sensing. Upon exposure to the three testing VOCs (i.e., ethanol, acetone, and isopropanol), the resonance wavelength demonstrated reversible concentration-dependent red shifts, allowing dynamic, real-time monitoring of adsorption–desorption kinetics. The sensitivity of these microcavities was attributed to the robust light confinement within the microcavity and the high reflectivity of the GIFs, which amplified light–molecule interactions. Average sensitivities for the RB spectral shifts were reported as follows: isopropanol [(10.4 ± 0.5) × 10^–3^ nm⋅(g⋅m^–3^)^–1^], ethanol [(8.9 ± 0.3) × 10^–3^ nm⋅(g⋅m^–3^)^–1^], and acetone [(4.3 ± 0.2) × 10^–3^ nm⋅(g⋅m^–3^)^–1^]. Discrimination between VOCs was achieved based on the unique spectral shift magnitudes and kinetic profiles, supported by their characteristic adsorption and desorption rates. The authors also presented a two-phase exponential desorption kinetic model described in this study, which effectively characterized different VOCs based on their desorption behavior within NAA–μCVs. The desorption process was divided into a fast initial phase, attributed to the removal of loosely adsorbed VOC molecules, and a slower phase corresponding to the release of strongly bound molecules from compact layers near the pore walls. This unique two-phase kinetic profile, influenced by VOC-specific interactions with the NAA nanopores (e.g., hydrogen bonding and polarity), provided a distinct chemical fingerprint for identifying and classifying different VOCs.

Expanding on the concept, Tran et al. developed a high-order Fabry–Pérot optical microcavity structure (NAA–HOμCV) using stepwise pulse anodization, wherein an extended-length defect layer embedded between the NAA–DBR mirrors was utilized to generate multiple RBs within the PSB. This extended-length microcavity configuration exhibited nine RBs that served as a powerful tool to discriminate between different gases by producing distinct spectral fingerprints upon exposure to each analyte (Fig. 9b) [198]. The NAA–HOμCVs were fabricated using SPA process in 1.1 M sulfuric acid at –2 °C, producing a layered structure comprising NAA–DBR mirrors separated by an extended-length cavity. The resulting structure featured a pore diameter of 10 ± 2 nm and a total film thickness of 14.1 ± 0.1 µm. Importantly, the sensor design did not rely on additional chemical functionalization, with the detection mechanism exploiting the intrinsic photonic properties of the NAA–HOμCVs. The sensors exhibited dynamic responses to isopropanol, ethanol, and acetone, with sensitivities of 0.43 ± 0.02 nm⋅(g⋅m^–3^)^–1^, 0.56 ± 0.03 nm⋅(g⋅m^–3^)^–1^, and 0.12 ± 0.01 nm⋅(g⋅m^–3^)^–1^, respectively. Notably, the structure demonstrated irreversible sensing, in this case, for silane molecules, detecting trace levels at concentrations as low as 4.54 ppb. This trace level sensing capability was attributed to the strong adsorption of silane molecules on the alumina surface, driven by their high chemical reactivity and ability to form stable surface bonds with the abundant surface area of the porous structure. The extended cavity length of the NAA–HOμCVs not only provided additional light recirculation paths for amplification of spectral shifts and improving the signal-to-noise ratio, but also allowed for multi-variable response patterns, enabling a single sensor to detect and identify multiple gas species based on their unique spectral responses. These advancements underscore the potential of high-order microcavities for creating compact and efficient systems for gas discrimination.

Building on the versatility of NAA–PC structures, they can be further engineered to create advanced architectures, such as integrating metal coatings to support Tamm plasmon modes, enabling enhanced sensing capabilities through tailored hybrid photonic–plasmonic interactions. Tran et al. presented a hybrid plasmonic–photonic gas sensing system for detecting ethanol and thiol-containing molecules, exploring Tamm plasmon resonances (TMM) configuration created at the interface between a dielectric NAA–PC and metallic layers (Fig. 9c) [199]. The NAA–PC layer featuring NAA–GIF configuration was fabricated using a two-step voltage-controlled SPA process in oxalic acid, with voltage modulated between 40 and 50 V. Pore widening treatment further refined the NAA–GIF structure, enhancing the effective refractive index contrast required for highly resolved PSBs. To create the hybrid TMM structure, thin films of Au, Ag, and Al were deposited onto the NAA surface using sputtering or thermal evaporation techniques, with Au and Ag achieving the highest performance at 19 nm, while Al exhibited optimal resonance quality at a thinner 9.5 nm due to reduced optical losses and interband transitions at this thickness. The optical properties of the TMM system were characterized by the formation of a sharp and distinct resonance dip (i.e., TMM band) within the broader PSB of the NAA–GIF structure. To evaluate sensing performance, the system was tested with various concentrations (i.e., from 0 to 30 ppm) of (3-mercaptopropyl)trimethoxysilane (MPTMS) in ethanol vapor mixture. The thiol (–SH) groups in MPTMS selectively interacted with metallic surfaces, forming self-assembled monolayers (SAMs) that altered the local effective refractive index at the plasmon–photonic interface. This interaction caused a linear red shift in the TMM resonance wavelength across the tested concentration range, which was monitored in real time using a UV–visible spectrometer. The hybrid sensor demonstrated high sensitivity to MPTMS, achieving a response of 0.038 ± 0.001 nm⋅ppm^–1^ and a LOD of 4.2 ppm with Ag coatings. Slightly lower sensitivities were observed with Au coatings (i.e., 0.018 ± 0.002 nm⋅ppm^–1^ and a LOD of 8.3 ppm) and Al coatings (i.e., 0.016 ± 0.001 nm⋅ppm^–1^ and a LOD of 9.4 ppm). These results highlight the potential of hybrid plasmonic–photonic structures based on TMM resonances for highly sensitive and selective gas detection applications.

### Colorimetric sensors

Colorimetric gas sensors offer a simple, cost-effective, and visually interpretable approach for gas detection, relying on perceptible color changes in response to chemical interactions. These sensors operate by exploiting specific optical transitions, such as absorption or reflection shifts, triggered by gas adsorption or chemical reactions on the sensing surface. Compared to other optical gas sensors, colorimetric sensors excel in their straightforward design, requiring minimal instrumentation and enabling rapid, on-site assessments. NAA serves as an exceptional platform for the design of colorimetric gas sensors because of its ability to enhance the optical and sensing properties critical for colorimetric detection. The tunable geometry of NAA nanopores allows precise modulation of optical properties, such as interference effects and light scattering, which are central to amplifying visible color changes. Additionally, the well-defined porous structure of NAA facilitates efficient immobilization of active sensing materials that undergo distinct color changes upon exposure to target gases. These materials interact directly with the confined environment of the nanopores, improving gas adsorption and reaction kinetics. This synergy between the nanostructure of NAA and the sensing material results in faster response times and more pronounced colorimetric signals. Recent advancements have demonstrated the versatility of NAA-based colorimetric sensors for detecting diverse analytes, including nitroaromatics, ammonia, and volatile acids [200–202].

**Fig. 10:**
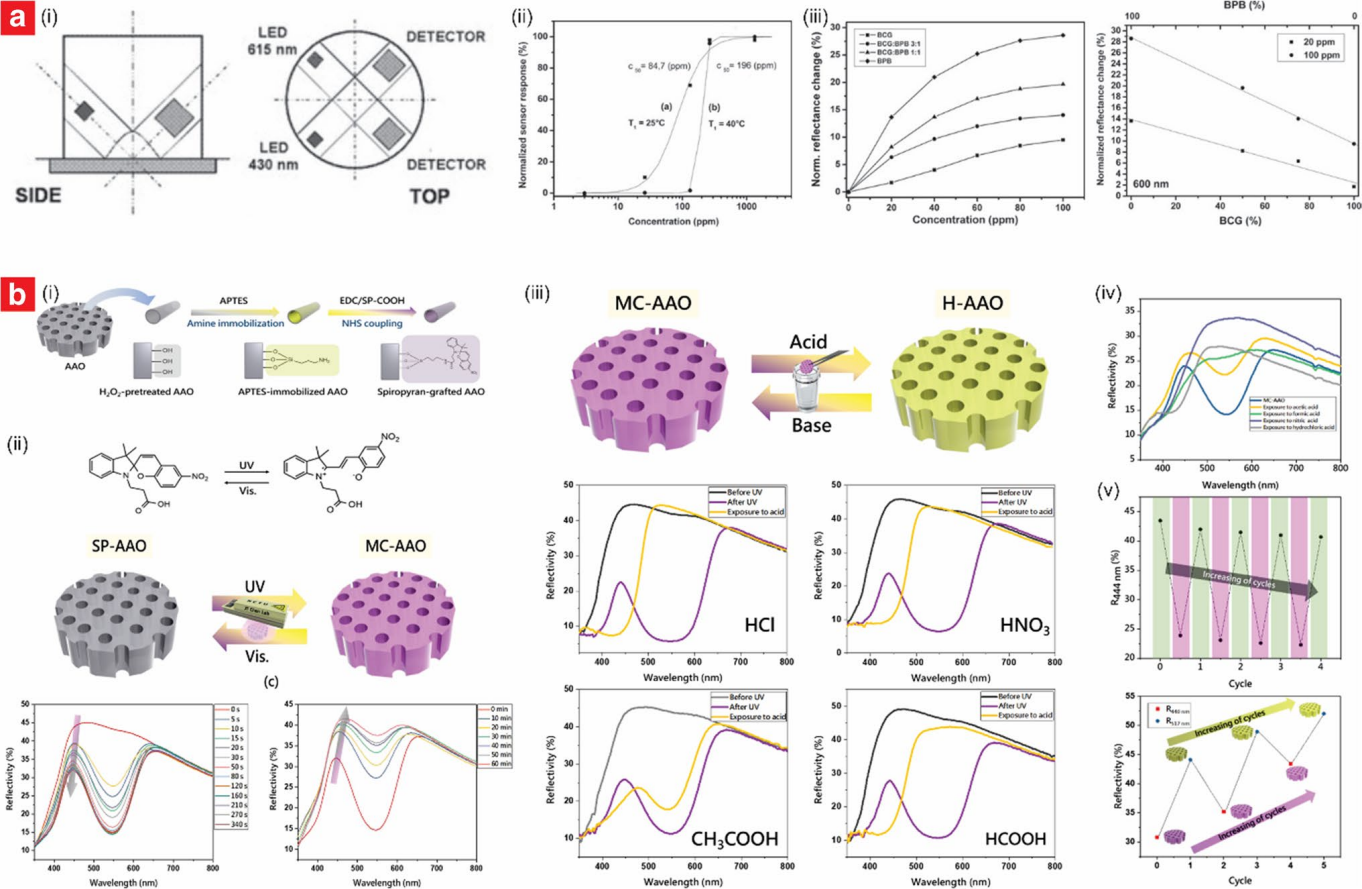
Engineering of NAA-based colorimetric gas sensors. **a** Colorimetric NH_3_ sensors based on NAA embedded with triphenyl-methane indicators: (i) schematic showing the side and top view of the sensor design; (ii) calibration curve of single-indicator BCG/NAA sensor measured at different temperatures; and (iii) calibration curves of the sensors using BCG and BPB indicators in different mass ratio upon exposure to various NH_3_ concentrations (reproduced from ref. [201] with permission from Taylor & Francis Group copyright 2014). **b** Engineering smart photo-/acid-responsive nanomembranes by grafting spiropyran molecules onto NAA templates for reversible and sensitive environmental pH monitoring: (i) synthesis of spiropyran-grafted NAA membranes; (ii) illustration of photochromism mechanism between SP-AAO and MC-AAO membranes and their corresponding UV–Vis spectra (ii) illustration of acidochromism between MC-NAA and H-NAA membranes and UV − visible diffuse reflectance spectra upon exposure to different acidic gases (e.g., HCl, HNO_3_, CH_3_COOH, HCOOH); (iv) side-by-side comparison of the reflectivity of an MC-AAO membrane upon exposure to various acid vapors; and (v) stability test during different cycles of protonation and deprotonation of the MC-NAA membranes (reproduced from ref. [202] with permission from American Chemical Society copyright 2020)

Markovics and Kovács developed a NAA-based colorimetric NH_3_ sensor for environmental applications, particularly in agriculture (Fig. 10a) [201]. The sensor design utilized triphenyl-methane acid–base indicators, including bromophenol blue (BPB) and bromocresol green (BCG), immobilized on the nanoporous NAA surface, leveraging a simple optical detection mechanism that eliminated the need for complex electronic systems. The anodization process, performed at 18 V in 5% sulfuric acid for 10 min, produced a nanoporous NAA layer with a uniform structure suitable for immobilization of dye indicators. Functionalization was achieved through adsorption of the indicators from an ethanol–water solution, followed by protonation with dodecylbenzenesulfonic acid to ensure chemical stability and responsiveness to NH_3_. The sensor operated by monitoring optical reflectance changes caused by the deprotonation of the immobilized indicators upon exposure to ammonia. These interactions resulted in color shifts that were measured spectrophotometrically or via a custom reflection-based interrogation unit. This simple device, equipped with LEDs and photodiodes, provided binary output signals indicating whether NH_3_ concentrations had reached preset thresholds. The sensors demonstrated a detection range between 31 and 69 ppm, depending on the indicator used. The device also exhibited tunable dynamic range and sensitivity through temperature adjustments, as increased temperature shifted the sensor’s calibration curves toward higher ammonia concentrations.

Further innovations combined the principles of photochromism with colorimetric detection. Lee et al. reported the development of a switchable, reusable, and highly sensitive gas sensor based on spiropyran-grafted NAA templates for detecting volatile acids (Fig. 10b) [202]. The sensor design leveraged the unique photochromic and acidochromic properties of spiropyran molecules, which were grafted onto porous NAA layers to create a functional sensing platform. The porous NAA templates (200-nm pore diameter) were pretreated with hydrogen peroxide to introduce hydroxyl groups and functionalized with (3-aminopropyl)triethoxysilane (APTES) to covalent attachment of spiropyran molecules through amide bond formation. This process ensured uniform spiropyran grafting while maintaining the structural integrity of the NAA for effective sensing. The detection mechanism of the sensor relied on the reversible isomerization of spiropyran to its merocyanine form under UV irradiation. The merocyanine was then protonated in the presence of volatile acids, resulting in a color change from purple to yellow. This acidochromic response was attributed to proton adsorption within the nanopores, which induced structural changes in the spiropyran molecules. The process was highly reversible, as exposure to visible light or base vapors restored the original state of the sensor. The sensor exhibited rapid response times of less than 10 s for strong acids like hydrochloric and nitric acids, while weaker acids such as acetic acid produced slower and less pronounced responses.

### Plasmonic effect–based sensors

Plasmonic effect–based gas sensors utilizing NAA materials exploit the localized surface plasmon resonance (LSPR) phenomenon, where oscillations of conduction electrons on the surface of metallic nanoparticles embedded in NAA are sensitive to changes in the surrounding refractive index, enabling high-sensitivity gas detection. LSPR arises from gas adsorption on metal nanoparticles that alter the refractive index of their surrounding environment, causing measurable spectral shifts in the LSPR signal. Several notable examples of plasmonic sensors integrating LSPR, Tamm plasmon resonance (TMM), and surface-enhanced Raman scattering (SERS) in NAA structures have been demonstrated. This highlights the emerging potential of NAA in plasmonics as a transformative platform for highly sensitive and specific gas detection [199, 203–206]. The integration of NAA into plasmonic sensor designs provides a nanostructured platform that stabilizes and uniformly distributes metal nanoparticles, enabling consistent and reproducible plasmonic responses. The high surface area of NAA porous platforms facilitates efficient gas diffusion, resulting in rapid response times and improved sensing signals. Additionally, the tunable geometry of NAA, which can be precisely engineered via anodization parameter modifications to create specialized optical structures that optimize light–matter interactions, can be combined with plasmonic designs to further amplify LSPR signals for precise gas detection.

Kim et al. developed a plasmonic optical gas sensing system for ethanol vapor detection, leveraging LSPR created by bi-metallic plasmonic system made of Ni and Au layers, and functional ZnO nanoparticles to achieve high sensitivity and a rapid response [203]. The NAA substrate was fabricated using the two-step anodization process in 3 M phosphoric acid at 110 V, with a second anodization step producing a 1-µm-thick layer optimized for sensitivity. To enhance LSPR effects, a thin layer of Au and Ni was deposited onto the NAA substrate using electron-beam evaporation. The LSPR signal was characterized by a strong reflectance band at 610 nm, observed through changes in reflected light intensity and spectral position using a reflectance optical probe and spectrometer. The sensing layer was created by depositing ZnO nanoparticles onto the NAA surface via spin coating. The ZnO nanoparticles (~ 3 nm) were synthesized using the sol–gel method, with size control achieved through variations in heating temperature and time. The detection mechanism relied on changes in the refractive index caused by ethanol adsorption onto the ZnO nanoparticle surface, which altered the LSPR conditions of the bi-metallic Ni and Au layers. These changes induced variations in the intensity and spectral position of reflected light, which were measured using a reflectance optical probe and analyzed with a spectrometer. The sensor exhibited a linear response to ethanol concentrations ranging from 0 to 100 ppb, with a LOD as low as 5 ppb and consistent sensitivity across the tested range.

**Fig. 11:**
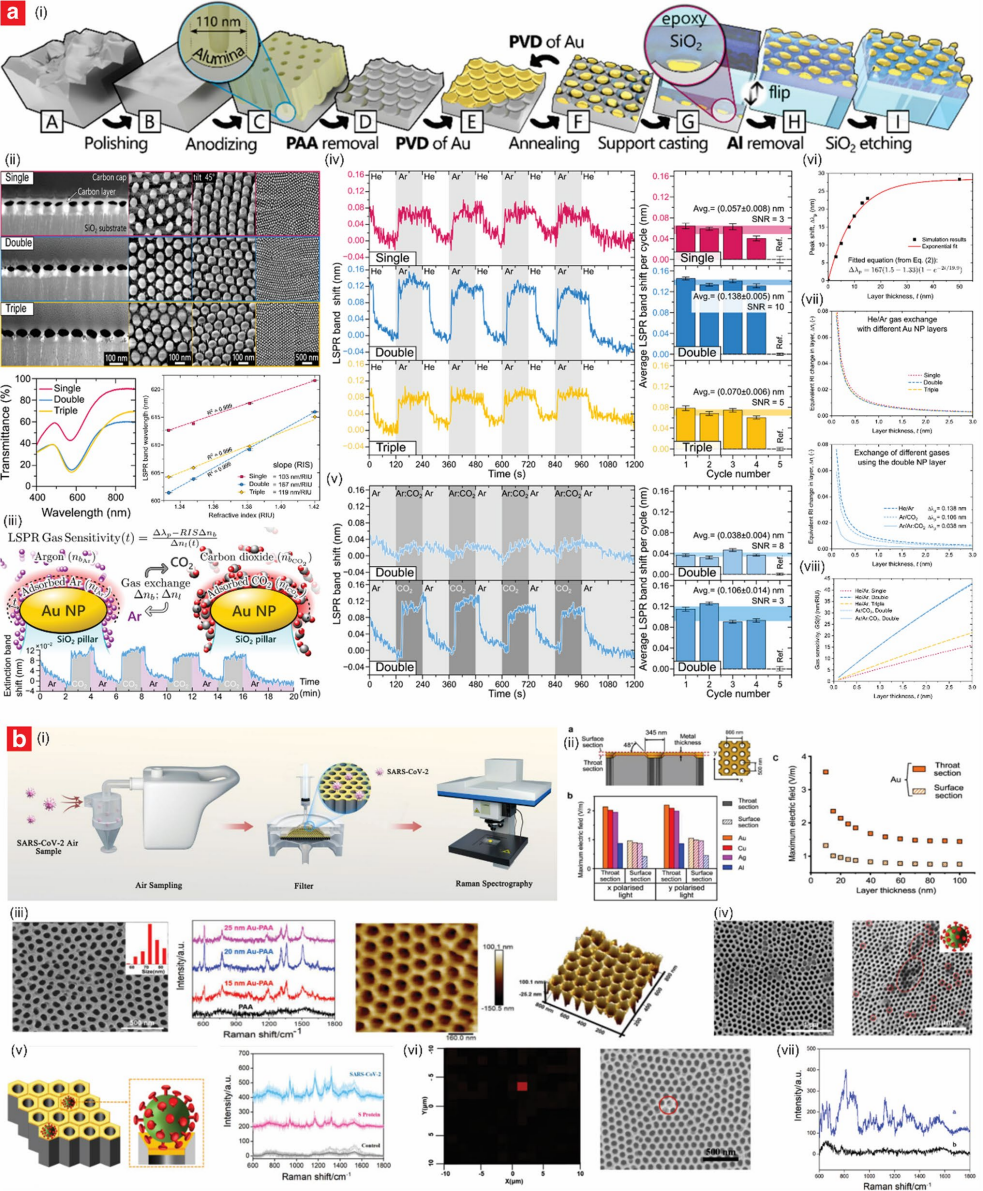
Engineering of NAA-based gas sensors utilizing plasmonic resonance transduction method. **a** Engineering hexagonally ordered Au NP layers on NAA templates for plasmonic gas sensing with a novel LSPR gas sensitivity benchmarking parameter: (i) Fabrication process of the Au NP layers; (ii) SEM images of three types of Au NP arrangements and tunning of their corresponding LSPR band wavelength; (iii) schematic depicting detection mechanism; LSPR band wavelength shift measurements in real-time during exposure to (iv) He and (v) CO_2_ versus Ar as baseline; and (vi)-(viii) LSPR gas sensitivity function *GS(t)* (reproduced from ref. [204] with permission from American Chemical Society copyright 2024). **b** Engineering dual-functional Au-NAA membranes for virus enrichment and label-free detection of airborne SARS-CoV-2 using SERS: (i) workflow of a rapid and label-free platform for SARS-CoV-2 enrichment and SERS identification; (ii) theoretical analysis and Raman results of different plasmonic components (e.g., Au, Cu, Ag, Al); (iii) structural characterization of the platform via SEM and AFM scans; and (iv) and (v) SERS detection of SARS-CoV-2 by the Au-NAA via Raman spectra measurements (reproduced from ref. [205] with permission from American Chemical Society copyright 2024)

Proença et al. developed a plasmonic gas sensor for detecting low-refractive-index gases at room temperature, utilizing densely packed ellipsoidal Au nanoparticle arrays. They introduced a novel LSPR gas sensitivity parameter to improve benchmarking accuracy and enable reliable performance evaluation (Fig. 11a) [204]. The sensor fabrication was based on NAA templates anodized at 40 V in 0.3 M oxalic acid (8 °C, 20 h) to create well-ordered nanopores. Au nanoparticle films were deposited via sputtering and transformed into discrete nanoparticles through solid-state dewetting and annealing, forming three types of arrangements (i.e., single, double, and triple layers) based on varying film thicknesses. These arrays were subsequently transferred onto glass substrates and embedded within SiO_2_ nanopillars to enhance plasmonic coupling and optical properties. The optical characteristics of the system were defined by its LSPR transmittance minimum band near a wavelength of 570 nm, which responded to changes in the effective refractive index caused by gas molecules interacting with the nanoparticle surfaces. The performance of the sensor was evaluated through its LSPR response to He and CO_2_ in Ar gas matrix (i.e., He/Ar and Ar/CO_2_). For He/Ar gas exchange, all three Au NP configurations—single, double, and triple layers—were examined, where the double-layer configuration exhibited the highest sensitivity, achieving a peak wavelength shift of 0.138 ± 0.005 nm, compared to 0.057 ± 0.008 nm for the single-layer and 0.070 ± 0.006 nm for the triple-layer. The superior performance of the double layer was attributed to its optimal nanoparticle size and interparticle coupling, which enhanced plasmonic field intensity. The double-layer configuration was tested for CO_2_ detection, showing an average wavelength shift of 0.106 ± 0.014 nm for pure CO_2_ and 0.038 ± 0.004 nm for a 20% CO₂ mixture in Ar, with a LOD estimated at 10% CO_2_ concentration. In addition, the authors introduced an LSPR Gas Sensitivity Function (*GS*(t)) as a benchmarking parameter to evaluate the performance of plasmonic gas sensors. This function helped address a critical limitation of conventional sensitivity metrics by considering the thin adsorbed gas layer that forms on the sensor surface, separated from the contributions of the bulk gas, providing a more accurate measure of the sensor’s ability to detect gases based on surface interactions.

Among plasmonic-effect-based optical gas sensors, SERS is a powerful spectroscopic technique that amplifies weak Raman signals of molecules absorbed on nanostructured metal surfaces, enabling detection at trace levels. This enhancement arises primarily from LSPR, which generates intense electromagnetic fields at the metal surface, and from charge transfer interactions between the adsorbed molecules and the metal. These interactions, involving electron transfer processes that modify molecular polarizability, further amplify the Raman signal. Han et al. introduced a dual-functional SERS-based sensor for detecting airborne viruses, utilizing a Au-coated NAA substrate that combined high virus enrichment efficiency with single-virus detection sensitivity (Fig. 11b) [205]. The NAA template was commercially obtained and featured hexagonally packed nanochannels with an initial pore size of approximately 90 nm. A 20-nm-thick Au layer was deposited onto the NAA surface via ion sputtering to enable plasmonic enhancement through near-field effects and the electromagnetic lightning rod effect, which were further amplified by the surface roughness of the Au layer. The detection mechanism relied on the highly efficient capture of virus particles through size exclusion within the nanochannels, followed by significant Raman signal amplification at the plasmonic hot spots located around the channel openings. This sensor demonstrated exceptional performance, achieving an enrichment efficiency of 98% for simulated particles and 86% for SARS-CoV-2 pseudoviruses. By identifying the distinct vibrational fingerprints of the SARS-CoV-2 spike protein, the sensor achieved single-virus sensitivity, a notable advancement over traditional detection systems that often require bulk sample processing or external filtration steps.

Oh et al. introduced a SERS-based gas sensor using a silver-coated aggregated nanowire structure with tapered nanogaps fabricated from NAA templates for benzene detection [206]. The NAA templates were fabricated using the two-step anodization process in oxalic acid, with a constant voltage of 40 V during the first anodization for 15 h, and 50 V during the second anodization for 1 h. Pores with an initial diameter of 30 nm were chemically etched in phosphoric acid, and during the drying process, the alumina nanowires aggregated spontaneously due to surface tension. This self-aggregation formed irregular bundles with pyramid-like geometries and tapered nanogaps narrowing from 50 to 0 nm, creating dense hot spots for plasmonic enhancement and significant Raman signal amplification. These nanowire bundles were subsequently coated with Ag or Au films of 60–120-nm thickness via thermal evaporation or sputtering to achieve the LSPR enhancement. To create a SERS-based sensor for benzene sensing, 1-propanethiol was used to form SAMs on the Ag-coated nanowires. This functionalization enhanced the adsorption of benzene molecules through hydrophobic interactions. The benzene detection mechanism involved weak physical adsorption of benzene molecules onto the 1-propanethiol SAMs, altering the local refractive index and enhancing the Raman signal. The sensor exhibited outstanding performance, achieving a LOD of < 0.3 ppm for benzene when the substrate was cooled to − 80 °C, with real-time detection occurring within 120 s. The plasmonic enhancement was attributed to the high electric field intensities generated in the tapered nanogaps of the nanowires. The SERS substrate demonstrated an enhancement factor of approximately 1.8 × 10⁷, with optimal performance achieved using a 100-nm silver coating, which provided a balance between nanogap density and light reflection efficiency. The sensor demonstrated high reproducibility, with a relative standard deviation of < 4%, and its response to benzene concentrations followed a linear relationship up to 5 ppm.

### Luminescence sensors

Luminescence-based gas sensors are valued for their high sensitivity, rapid response times, and adaptability to various environmental conditions. These sensors typically consist of luminescent materials, intrinsic or extrinsic embedded in a supporting matrix, such as a porous substrate or thin film. By leveraging the emission of light through fluorescence or phosphorescence from these materials, they detect target gases via measurable changes in luminescence intensity or other spectral properties. Besides their core functionality, luminescence-based sensors offer additional advantages that enhance their versatility. For instance, they enable non-invasive and remote sensing, which is critical for monitoring gases in hazardous or hard-to-reach environments. Their ability to detect gases across a wide range of concentrations further broadens their application scope. Tailored luminescent dyes and materials allow for improved and tailorable selectivity, ensuring accurate detection of specific gases, while their compact and lightweight designs support integration into portable and wearable devices. Furthermore, their compatibility with advanced microfluidic and nanophotonic systems positions them as integral components in cutting-edge sensing technologies. NAA templates have proven to be highly suitable platforms for luminescence-based sensing. The well-ordered nanoporous structures of NAA offer a large surface area, promoting efficient interaction between gas molecules and luminescent materials. This unique architecture not only enhances sensitivity and stability but also supports the straightforward integration of luminescent dyes within the pores. The optical transparency of NAA further facilitates its application in optical sensing systems, while the simplicity of its fabrication processes ensures scalability and cost-effectiveness. These advantages have been instrumental in studies that explore sophisticated sensing mechanisms, such as fluorescence quenching, excimer formation, and phosphorescence enhancement, demonstrating the potential of NAA templates to elevate sensor performance.

The development of fluorescence-based O_2_ sensors using pyrene derivatives has shown promising advancements. Fujiwara et al. introduced a fluorescence O_2_ sensor based on a pyrene-1-butyric acid (PBA) chemisorption film on a NAA platform [207]. The anodization of NAA substrate was performed in a 1.0 M H_2_SO_4_ solution, followed by post-anodization treatment with pore-widening process to create a controlled-pore structure. The PBA functional film was formed by immersing the NAA plate into a 0.1 mmol⋅L^–1^ ethanol solution of pyrene-1-butyric acid (PBA), a polycyclic aromatic hydrocarbon with strong fluorescence and a high quantum yield. Functionalization with PBA introduces a carboxyl group, which forms a stable chemisorbed layer on the aluminum oxide surface, facilitating efficient oxygen interaction. This chemisorbed layer enables fluorescence quenching upon exposure to O_2_, making it highly effective for optical sensing applications. The sensor demonstrated strong sensitivity to O_2_, achieving a *I*_*0*_*/I*_*100*_ value of 6.14 ± 0.15, significantly surpassing earlier designs based on silicone (Si) polymer films. Its fluorescence quenching mechanism, involving both dynamic (collisional interactions) and static (ground-state complex formation) quenching processes, revealed two distinct O_2_-accessible sites. The first, more accessible site exhibited a quenching constant (*K*_*SV1*_) of 0.028 Torr^–1^, while the second, less accessible site showed a lower constant (*K*_*SV2*_) of 0.00086 Torr^–1^. These characteristics were well-captured by a modified Stern–Volmer equation, enabling precise calibration of the sensor. The sensor exhibited fast and reversible response times, requiring 10 s to switch from Ar to O_2_ and 53 s for the reverse process, while maintaining reliable performance under alternating O_2_ and Ar atmospheres without hysteresis, demonstrating its resilience and reliability. Its robust photostability was confirmed, with only a 10% decrease in fluorescence intensity after 24 h of continuous irradiation with a 150-W tungsten lamp.

Building on this work, Fujiwara et al. continued to refine the sensor design by incorporating myristic acid into the chemisorption layer of PBA on NAA platform [208]. The addition of myristic acid was designed to control the spatial arrangement of PBA molecules and promote efficient excimer formation, thereby enhancing fluorescence quenching sensitivity for O_2_ detection and allowed the sensor to better discriminate O_2_ from other quenchers. The NAA substrate was fabricated through anodization in 0.3 M oxalic acid under controlled current density conditions of 2 mA⋅cm^–2^ at a temperature of 10 °C for 10 min to produce a uniform nanoporous structure. The PBA functional layer was immobilized on the NAA surface using the same procedure in their previous study. Myristic acid was subsequently introduced to fine-tune the molecular arrangement of PBA, ensuring optimal conditions for excimer formation and enhanced interaction with O_2_ molecules. The sensor exhibited excellent sensitivity to O_2_ concentrations ranging from 0 to 160 Torr, with a linear Stern–Volmer response (KSV of 0.0195 Torr^–1^) indicating strong fluorescence quenching, while maintaining operational stability with fluorescence intensity variations below 5% and rapid response times of 5–10 s for multiple O_2_ exposure and recovery cycles. The excimer fluorescence intensity decreased significantly, with approximately 60% quenching observed in O_2_ compared to nitrogen environments. The incorporation of myristic acid enhanced performance by stabilizing the chemisorption layer and reducing non-specific quenching, leading to a ~ 15% improvement in signal-to-noise ratio and a more stable fluorescence intensity baseline.

**Fig. 12:**
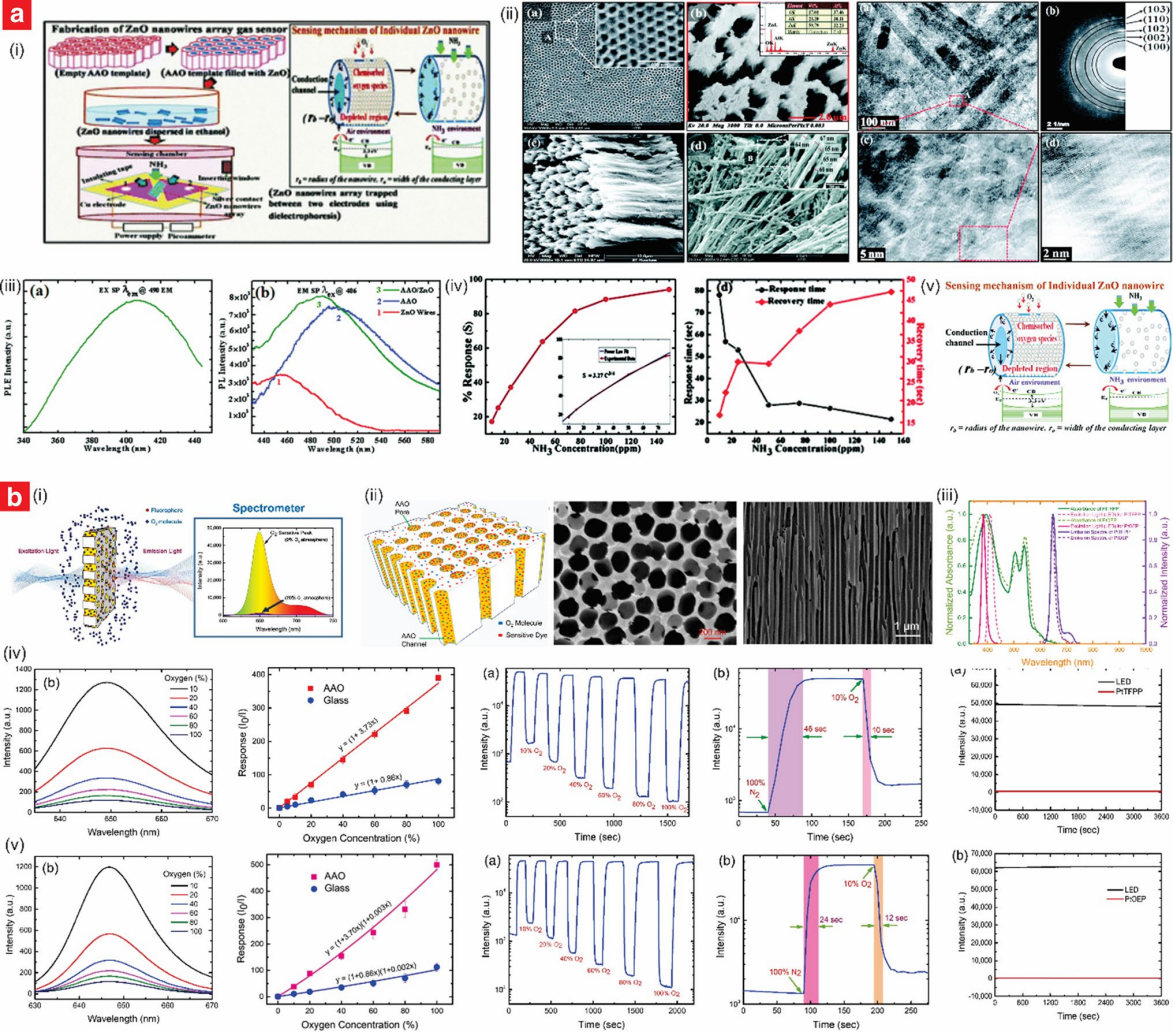
Engineering of NAA-based luminescence gas sensors. **a** Engineering luminescent ZnO nanowire arrays via NAA-assisted template synthesis for NH_3_ gas sensing at room-temperature with rapid response and recovery: (i) design and concept of the sensor; (ii) structural characterization via SEM and TEM imaging and (iii) photoluminescence spectra of ZnO/NAA assemblies; (iv) sensing response and kinetics of ZnO/NAA sensor during exposure to various NH_3_ concentrations; and (v) illustration of sensing mechanism (reproduced from ref. [134] with permission from The Royal Society of Chemistry copyright 2014). **b** Engineering phosphorescence-based optical O_2_ sensors using PtTFPP and PtOEP dyes on NAA through-hole membranes: (i) schematic of the dye-embedded NAA sensor concept; (ii) schematic showing dye-embedded NAA platform and structural characterizations with SEM images; (iii) photoluminescence spectra of NAA platform embedded with PtTFPP and PtOEP dyes; row (iv) and (v), from left to right: emission spectra, response versus O_2_ concentrations curve, real-time dynamic response curves, and photostability test under continuous illumination of PtTFPP and PtOEP dyes on NAA through-hole membranes, respectively (reproduced with permission from ref. [210] copyright 2022)

In another study, Kumar et al. presented a room-temperature NH_3_ gas sensor leveraging luminescent ZnO nanowires synthesized using an NAA-assisted template method (Fig. 12a) [134]. The NAA templates, featuring nanopores with diameters of 45–50 nm and a thickness of 12 μm, were prepared by anodization (0.3 M oxalic acid at 40 V and 3 °C) and subsequent post-anodization pore widening. These templates facilitated the synthesis of vertically aligned ZnO nanowires through vacuum infiltration with a saturated zinc nitrate solution, followed by an annealing at 435 °C for 40 h to form wurtzite ZnO structures. The nanowires were then aligned between copper electrodes on a glass substrate using dielectrophoresis with an AC electric field of 15 Vpp at 0.5 MHz. The ZnO nanowire sensor demonstrated highly effective performance for NH_3_ detection at room temperature, achieving a sensitivity of 68% at 50 ppm with a linear response range extending to 75 ppm. The response and recovery times were rapid, recorded at 28 and 29 s, respectively. This remarkable performance was attributed to the ordered structure and high surface-to-volume ratio of the ZnO nanowires, which promoted efficient gas interaction. Additionally, the luminescent properties of the nanowires, characterized by a green emission at 490 nm due to oxygen vacancy defects, enhanced the functionality of the sensor by facilitating charge transfer during gas adsorption and reaction. The detection mechanism was based on the interaction between NH_3_ molecules and adsorbed oxygen ions (O_2_^–^) on the ZnO surface. Adsorbed O_2_^–^ ions created a depletion region by extracting electrons from the conduction band of ZnO. Exposure to NH_3_ molecules reduced the depletion width by releasing trapped electrons, thereby decreasing the resistance. This response followed a power-law relationship (S = AC^α^) with α ≈ 0.75, demonstrating predictable and reproducible behavior of this sensing system.

Phosphorescence-based sensors are another category of luminescence sensors that has also been explored for O_2_ sensing application [209, 210]. Liu et al. introduced a phosphorescence-based oxygen sensor utilizing through-hole NAA substrates, achieving superior sensitivity and stability through the integration of Pt-based porphyrins and the unique high-surface-area nanochannel structure of NAA (Fig. 12b) [210]. The NAA membranes, commercially obtained, featured through-hole nanopores with a diameter of approximately 200 nm and a thickness of 60 µm. Two phosphorescent dyes, platinum(II) meso-tetrakis(pentafluorophenyl)porphyrin (PtTFPP) and platinum(II) octaethylporphyrin (PtOEP), were embedded into a sol–gel matrix comprising tetraethyl orthosilicate, n-octyltriethoxysilane, ethanol, hydrogen chloride, and Triton X-100. Each dye mixture was dissolved in tetrahydrofuran (THF) and individually loaded into the NAA substrates via spin-coating method. The use of NAA provided a high-surface-area platform with uniform nanochannels, facilitating efficient gas–dye interactions that amplified the O_2_ response. The sensor detected O_2_ via phosphorescence quenching, driven by Dexter energy transfer, where O_2_ molecules facilitated radiation-less transitions from the excited triplet states of the dyes to the ground state. Stern–Volmer plots revealed predominantly static quenching, with minimal dynamic contributions, allowed for strong, linear response to O_2_ presence. The PtTFPP sensor exhibited a maximum response (*I*_*0*_*/I*) of approximately 400 at 100% O_2_, with a *K*_*SV*_ of 3.73%^–1^—four times greater than the glass-based version. Similarly, the PtOEP sensor achieved a response of ~ 500 at 100% O_2_, with a *K*_*SV*_ value of 3.7%^–1^. The enhanced sensitivity was attributed to the structural characteristics of the NAA nanochannels, which increased the gas–dye interaction area and amplified the sensor response. Dynamic response times further underscored the practical feasibility of these sensors. The PtTFPP-based sensor exhibited response and recovery times of ~ 12 and ~ 24 s, respectively, while the PtOEP sensor showed ~ 10 s for response and ~ 46 s for recovery. Both sensors maintained high photostability, with less than 5% reduction in phosphorescence intensity after continuous LED illumination for 1 h. Moreover, their performance remained stable across varying humidity levels (55 to 75% RH) and under minor temperature fluctuations.

## NAA-based gas sensors with miscellaneous sensing principles

NAA provides a flexible platform for gas sensing beyond conventional electrochemical and optical methods, demonstrating high performance through alternative sensing principles such as surface acoustic wave (SAW), ionization, Schottky barrier, ionic transport mechanisms, and laser-excitation spectroscopy. These methods exploit the unique structure of NAA to achieve superior sensitivity and selectivity.

NAA-based gas sensors developed based on SAW technique leverage the inherent properties of acoustic wave detection technique to achieve efficient gas detection with fast response and recovery at room temperature [125, 211]. These sensors benefit from the unique capability to directly detect mass changes with high sensitivity through shifts in wave propagation velocity caused by adsorbed mass, and inherently avoids electromagnetic interference because of the absence of reliance on electrical or optical signal propagation. Early demonstrations of NAA-based SAW sensors for ethanol detection showed that tailoring the pore geometry of the porous NAA layer significantly enhanced sensitivity, by up to an order of magnitude, compared to uncoated SAW devices, an improvement attributed to the high adsorption capacity of the nanostructured porous film [211]. The applicability of NAA-based SAW sensors to NH_3_ detection has also been presented, with frequency shifts of approximately 0.001% per percentage change in NH_3_ concentration observed [125]. These sensors demonstrated reversible and stable response to NH_3_ at a concentration as low as 18 ppm, with response and recovery times of ~ 30–40 s and ~ 60–80 s, respectively.

Gas ionization sensor is another type of sensing device that operates based on the principle of field ionization, where strong electric fields at sharp nanotips ionize gas molecules, producing measurable electrical signals that correlate with the gas type and concentration. Integration of MWCNTs arrays within NAA templates has been explored for field ionization sensors, providing uniform ionization for detecting organic vapors like acetic acid and ethanol [212]. The sensors demonstrated high sensitivity to acetic acid in the gas phase, which was attributed to high static polarizability (5.105 Å^3^) of acetic acid vapor and low ionization energy (10.37 eV). The sensor reliably detected acetic acid via monitoring of pre-discharge current and breakdown voltage, which reached 7.01 pA at a low concentration of 50 µg∙L^–1^.

NAA-based Schottky barrier sensors leverage the unique properties of metal–semiconductor junctions to achieve high sensitivity and selectivity for gas detection, exemplified by their application in H_2_ sensing with advanced nanostructures such as Nb_2_O_5_ nanorods and Pd-decorated SnO_2_ nanotubes. These sensors detect gases by utilizing the modulation of the Schottky barrier height at the metal–semiconductor interface caused by gas adsorption, which alters carrier density and generates measurable changes in electrical resistance or current. The sensitivity can be further enhanced by catalytic metals and nanostructures that promote gas dissociation and efficient electron transfer. Nb_2_O_5_ nanorods arrays fabricated via NAA-template assisted anodization combined with a Pt/NiCr top electrode layer, forming a Schottky junction at the Pt/Nb_2_O_5_ interface, was demonstrated to create Schottky barrier sensor. H_2_ molecules dissociated on the Pt surface, forming atomic H that diffused to the junction, reducing the Schottky barrier height and altering the electrical conductivity. The sensor exhibited a response of 17 at 1000 ppm H_2_, with a response time of 3.5 min and a recovery time of 8.5 min [175]. Another H_2_ gas sensor design using nano-Schottky-junction-engineered Pd-decorated SnO_2_ nanotube arrays on a 3D porous NAA substrate. The sensor demonstrated remarkable sensitivity to H_2_ at room temperature, with a LOD as low as 1.6 ppb and a response of 3.54 for 200 ppm H_2_. The response increased linearly with concentration up to 1000 ppm, and the sensor exhibited excellent selectivity for H_2_ over other gases such as ethanol, toluene, and formaldehyde. Stability tests showed minimal degradation in performance over 100 days, indicating long-term reliability. The response and recovery times were rapid, supporting the applicability of this sensor design in real-time monitoring [213].

Ionic transport-based sensors detect gases by monitoring changes in ionic current or rectification within a confined nanostructure, where gas interactions with functionalized surfaces modulate ion transport properties, enabling precise and selective detection. A design combing intrinsic ionic rectification ability of NAA membranes, enhanced through functionalization with spiropyran molecules has created an optical-activated, highly sensitive SO_2_ gas sensor. The NAA membrane, which inherently rectified ionic flow due to its BOL, was further enhanced by functionalizing the nanochannel surfaces with SP molecules. This functionalization extended the rectification ability along the pore length and enabled SO₂ detection upon UV light activation. The sensor demonstrated exceptional performance in detecting SO_2_ over a dynamic range from 10 nM to 1 mM, with a LOD as low as 10 nM. The response current increased significantly, reaching 237 μA at 2 V after UV activation, indicating strong ionic rectification [214].

Reflection spectroscopy, though a common technique in optical analysis, represents a less frequently explored detection mechanism for developing NAA-based optical gas sensors. Reflection-based gas sensors utilizing a novel combination of functional materials with NAA benefited from the reflective aluminum support, eliminating the need for external reflective backgrounds required for glass-based sensors. These sensors also highlighted integration with other materials, which enhanced sensing efficiency and simplified the overall sensor design for NH_3_ detection, as demonstrated in the studies by Markovics and co-workers [215, 216]. The use of triphenyl-methane dyes (BPB, BCG, BCP) immobilized on NAA surfaces has been demonstrated via a cost-effective and reproducible adsorption process, with the dyes directly embedded within the NAA nanopores [215]. NAA platform fabricated at optimal conditions (e.g., 12 V anodization in 5% sulfuric acid) yielded sensors with a LOD of 18 ppm, a dynamic range of 6–80 ppm, and rapid response times (less than 10 s) based on acid–base interactions between NH_3_ and the immobilized dyes. Expanding on this design, BCG-containing ormosil coatings, an organically modified silica film derived from sol–gel technique, has been introduced to create reflection-based NAA gas sensors [216]. The ormosil coatings on NAA platform (anodized at 24 V in 5% sulfuric acid) demonstrated broader dynamic ranges (0–80 ppm) for NH_3_ detection, with a LOD of 6 ppm, and high mechanical stability, though with slightly slower recovery times due to gas adsorption in the pores. These examples underscore the versatility of NAA in supporting diverse gas sensing principles, cementing its role as a robust and adaptable platform for advanced applications.

## Conclusion

This review has provided an up-to-date analysis of the latest advancements in NAA-based gas sensors, offering both foundational insights and practical perspectives on their diverse architecture and applications in gas sensing. Key detection principles and notable developments in sensors utilizing optical and electrochemical transduction mechanisms have been presented in detail. NAA exhibits key features that make this material highly attractive for gas sensor development. The large surface area, highly ordered and tunable pore structures, unique chemical and thermal stability, and ease of functionalization make NAA a versatile platform for diverse gas sensing mechanisms. NAA structures have been combined with optical techniques (e.g., photoluminescence, reflection, and transmission) and electrochemical methods (e.g., impedance spectroscopy, chemiresistive sensing, catalytic detection) to detect a range of target gas analytes. The NAA structures have also been integrated with other functional materials such as metal nanoparticles, metal oxides, and carbon-based composites into the NAA matrix. This has provided a path for enhancing selectively by engineering the way in which this platform interacts with gas molecules, which in turn improves the overall sensing performance to meet the stringent demands of modern gas sensing applications. In addition to that, the structural engineering of NAA with complex pore configurations and tailored chemical modifications has expanded further its potential for developing multi-functional and high-performance sensing devices. Despite significant advancements, to fully unlock the potential of NAA-based gas sensors remains challenging. A common gap in the broader field of gas sensor development, which is also inherent to NAA-based sensors, is the ability to accurately discriminate between different gases in a matrix of multiple components. This capability is crucial for high-precision applications, such as environmental monitoring and industrial safety. Addressing this issue will require innovative approaches, including the design of multi-output sensors, sensor arrays with complementary responses, or the integration of advanced data processing algorithms, such as machine learning or pattern recognition, to effectively analyze and interpret complex sensor signals. Another significant gap specific to NAA-based gas sensors is the under-utilization of the inherent optical properties of NAA material. While simple NAA optical structures have been employed, much of the current research relies heavily on combining these basic structures with complex functional materials to achieve selective gas sensing, thereby placing the focus on the functional materials rather than NAA itself. This approach leaves the true potential of NAA almost unexplored, particularly its ability to produce diverse and tunable optical structures through controlled anodization. Proof-of-concept studies in this space have demonstrated that, by leveraging the versatility of NAA to create advanced optical structures (e.g., photonic crystals, Tamm plasmon configurations, or waveguides) researchers could unlock new opportunities for optical gas sensing. These advancements could lead to the development of sensors with unique optical responses tailored to specific gas analytes or single sensors with multiple optical outputs capable of simultaneously detecting different gas properties based on optical fingerprints. When combined with discriminating algorithms, such as machine learning, these sensors could maximize their potential for analyzing complex gas mixtures while reducing reliance on external functional materials. Further advancements in NAA-based sensors will also depend on integration into IoT-enabled devices and lab-on-chip systems, which offers significant potential for compact, real-time monitoring solutions. Moreover, scalable and eco-friendly fabrication methods, such as hybrid pulse anodization, are critical for achieving cost-effective production and enabling widespread adoption. Addressing these challenges and focusing on emerging trends can push the boundaries of NAA-based sensor technology, paving the way for transformative applications.

In summary, NAA-based gas sensors offer a transformative platform for advancing gas sensing technologies across diverse applications. The versatility of NAA, combined with its ability to be integrated with advanced materials and leverage innovative structural designs, places this technology in a perfect situation to develop promising solutions for next-generation sensing systems. Future efforts are expected to focus on harnessing tunable optical properties and structural adaptability of NAA to create sensors with enhanced precision, multi-functional capabilities, and real-time monitoring potential. Looking ahead, sustained research into optical structuring, real-time integration, and scalable fabrication will be key to realizing the full potential of NAA-based sensors in next-generation monitoring systems.

## Data Availability

No datasets were generated or analysed during the current study.

## References

[1] Asri MIA, Hasan MN, Fuaad MRA et al (2021) MEMS gas sensors: a review. IEEE Sens J 21(17):18381–18397. 10.1109/JSEN.2021.3091854

[2] Dhall S, Mehta BR, Tyagi AK et al (2021) A review on environmental gas sensors: materials and technologies. Sensors International 2:100116. 10.1016/j.sintl.2021.100116

[3] Korotcenkov G (2013) *Handbook of Gas Sensor Materials: Properties, Advantages and Shortcomings for Applications Volume 1: Conventional Approaches*. (Integrated Analytical Systems). New York, NY: Springer

[4] Wu J, Yue G, Chen W et al (2020) On-chip optical gas sensors based on group-IV materials. ACS Photonics 7(11):2923–2940. 10.1021/acsphotonics.0c00976

[5] Cho SH, Suh JM, Eom TH et al (2021) Colorimetric sensors for toxic and hazardous gas detection: a review. Electron Mater Lett 17(1):1–17. 10.1007/s13391-020-00254-9

[6] Tian X, Cui X, Lai T et al (2021) Gas sensors based on TiO2 nanostructured materials for the detection of hazardous gases: a review. Nano Materials Science 3(4):390–403. 10.1016/j.nanoms.2021.05.011

[7] De Fazio R, Al-Hinnawi A-R, De Vittorio M et al (2022) An Energy-autonomous smart shirt employing wearable sensors for users’ safety and protection in hazardous workplaces. Applied Sciences 12(6). 10.3390/app12062926

[8] Ates HC, Brunauer A, von Stetten F et al (2021) Integrated devices for non-invasive diagnostics. Adv Func Mater 31(15):2010388. 10.1002/adfm.202010388

[9] Vajhadin F, Mazloum-Ardakani M, Amini A (2021) Metal oxide-based gas sensors for the detection of exhaled breath markers. MEDICAL DEVICES & SENSORS 4(1):e10161. 10.1002/mds3.10161 33615149 10.1002/mds3.10161 PMC7883254

[10] Zong B, Wu S, Yang Y et al (2024) Smart gas sensors: recent developments and future prospective. Nano-Micro Letters 17(1):54. 10.1007/s40820-024-01543-w 39489808 10.1007/s40820-024-01543-w PMC11532330

[11] Panda S, Mehlawat S, Dhariwal N et al (2024) Comprehensive review on gas sensors: unveiling recent developments and addressing challenges. Mater Sci Eng, B 308:117616. 10.1016/j.mseb.2024.117616

[12] Dai Z, Ju H (2012) Bioanalysis based on nanoporous materials. TrAC, Trends Anal Chem 39:149–162. 10.1016/j.trac.2012.05.008

[13] B S, John A, Panda PK (2021) A review on metal-oxide based p-n and n-n heterostructured nano-materials for gas sensing applications. Sensors International 2:100085. 10.1016/j.sintl.2021.100085

[14] Bhati VS, Kumar M, Banerjee R (2021) Gas sensing performance of 2D nanomaterials/metal oxide nanocomposites: a review. Journal of Materials Chemistry C 9(28):8776–8808. 10.1039/D1TC01857D

[15] Isaac NA, Pikaar I, Biskos G (2022) Metal oxide semiconducting nanomaterials for air quality gas sensors: operating principles, performance, and synthesis techniques. Microchim Acta 189(5):196. 10.1007/s00604-022-05254-0 10.1007/s00604-022-05254-0 PMC9023411 35445855

[16] Losic D, Santos A (2015) *Nanoporous Alumina : Fabrication, Structure, Properties and Applications*. (Springer Series in Materials Science, 219). Cham: Springer International Publishing

[17] Lee W, Park S-J (2014) Porous anodic aluminum oxide: anodization and templated synthesis of functional nanostructures. Chem Rev 114(15):7487–7556. 10.1021/cr500002z 24926524 10.1021/cr500002z

[18] Santos A (2017) Nanoporous anodic alumina photonic crystals: fundamentals, developments and perspectives. Journal of Materials Chemistry C 5(23):5581–5599. 10.1039/C6TC05555A

[19] Taşaltın N, Öztürk S, Kılınç N et al (2011) Investigation of the hydrogen gas sensing properties of nanoporous Pd alloy films based on AAO templates. J Alloy Compd 509(14):4701–4706. 10.1016/j.jallcom.2011.01.081

[20] Yim C, Lee M, Yun M et al (2015) CO2-selective nanoporous metal-organic framework microcantilevers. Sci Rep 5(1):10674. 10.1038/srep10674 26035805 10.1038/srep10674 PMC4451844

[21] Santos A, Kumeria T, Losic D (2013) Nanoporous anodic aluminum oxide for chemical sensing and biosensors. TrAC, Trends Anal Chem 44:25–38. 10.1016/j.trac.2012.11.007

[22] Law CS, Lim SY, Abell AD et al (2018) Nanoporous Anodic alumina photonic crystals for optical chemo- and biosensing: fundamentals, advances, and perspectives. Nanomaterials 8(10). 10.3390/nano8100788 10.3390/nano8100788 PMC6215225 30287772

[23] Wang J, Vu KN, Abell AD et al (2023) Nanoporous anodic alumina-based iontronics: fundamentals and applications. Journal of Materials Chemistry C 11(27):9051–9081. 10.1039/D3TC01735D

[24] Eftekhari A (2008) Nanostructured materials in electrochemistry. Wiley-VCH, Weinheim, p 2008

[25] Masuda H, Fukuda K (1995) Ordered metal nanohole arrays made by a two-step replication of honeycomb structures of anodic alumina. Science (American Association for the Advancement of Science) 268(5216):1466–1468. 10.1126/science.268.5216.1466 10.1126/science.268.5216.1466 17843666

[26] Masuda H, Hasegwa F, Ono S (1997) Self-ordering of cell arrangement of anodic porous alumina formed in sulfuric acid solution. J Electrochem Soc 144(5):L127. 10.1149/1.1837634

[27] Masuda H, Yada K, Osaka A (1998) Self-ordering of cell configuration of anodic porous alumina with large-size pores in phosphoric acid solution. Jpn J Appl Phys 37(11A):L1340. 10.1143/JJAP.37.L1340

[28] Jessensky O, Müller F, Gösele U (1998) Self-organized formation of hexagonal pore structures in anodic alumina. J Electrochem Soc 145(11):3735. 10.1149/1.1838867

[29] Lee W, Ji R, Gösele U et al (2006) Fast fabrication of long-range ordered porous alumina membranes by hard anodization. Nat Mater 5(9):741–747. 10.1038/nmat1717 16921361 10.1038/nmat1717

[30] Yanagishita T, Kawato R, Masuda H (2022) Highly ordered anodic porous alumina prepared by anodization of Al in extremely dilute H2SO4. J Electrochem Soc 169(7):073504. 10.1149/1945-7111/ac7dc9

[31] Terashima A, Iwai M, Kikuchi T (2022) Nanomorphological changes of anodic aluminum oxide fabricated by anodizing in various phosphate solutions over a wide pH range. Appl Surf Sci 605:154687. 10.1016/j.apsusc.2022.154687

[32] Li S-Y, Liu Z-L, Xiang G-X et al (2022) Photoluminescence properties of anodic aluminium oxide films formed in a mixture of malonic acid and oxalic acid. Luminescence 37(11):1864–1872. 10.1002/bio.4363 35977810 10.1002/bio.4363

[33] Chernyakova K, Vrublevsky I, Jagminas A et al (2021) Effect of anodic oxygen evolution on cell morphology of sulfuric acid anodic alumina films. J Solid State Electrochem 25(4):1453–1460. 10.1007/s10008-021-04925-x

[34] Vrublevsky I, Lushpa N, Chernyakova K et al (2020) "Investigation of the mechanism of electronic conductivity and parameters of localized states in porous anodic alumina films obtained in phosphoric acid," in *2020 IEEE International Conference on Electrical Engineering and Photonics (EExPolytech)* pp. 219–221, 10.1109/EExPolytech50912.2020.9243857.

[35] Roslyakov IV, Kolesnik IV, Levin EE et al (2020) Annealing induced structural and phase transitions in anodic aluminum oxide prepared in oxalic acid electrolyte. Surf Coat Technol 381:125159. 10.1016/j.surfcoat.2019.125159

[36] Garcia-Vergara SJ, Skeldon P, Thompson GE et al (2007) Stress generated porosity in anodic alumina formed in sulphuric acid electrolyte. Corros Sci 49(10):3772–3782. 10.1016/j.corsci.2007.03.036

[37] Sagar S, Mohammadian N, Park S et al (2020) Ultra-thin anodized aluminium dielectric films: the effect of citric acid concentration and low-voltage electronic applications. Nanotechnology 31(25):255705. 10.1088/1361-6528/ab7fd1 32168504 10.1088/1361-6528/ab7fd1

[38] Schneider M, Langklotz U, Kühne L et al (2024) Investigation of anodic oxide formation on AA 7075 in citric acid. Mater Chem Phys 320:129458. 10.1016/j.matchemphys.2024.129458

[39] González-Rovira L, González-Souto L, Astola PJ et al (2020) Assessment of the corrosion resistance of self-ordered anodic aluminum oxide (AAO) obtained in tartaric-sulfuric acid (TSA). Surf Coat Technol 399:126131. 10.1016/j.surfcoat.2020.126131

[40] Brzózka A, Brudzisz A, Rajska D et al (2020) "Chapter two - recent trends in synthesis of nanoporous anodic aluminum oxides," in *Nanostructured Anodic Metal Oxides*, G. D. Sulka Ed.: Elsevier pp. 35–88.

[41] Zahariev A, Kanazirski I, Girginov A (2008) Anodic alumina films formed in sulfamic acid solution. Inorg Chim Acta 361(6):1789–1792. 10.1016/j.ica.2007.03.040

[42] Gunenthiran S, Wang J, Law CS et al (2025) Nanoporous anodic alumina photonic crystals for solid-state lasing systems: state-of-the-art and perspectives. Journal of Materials Chemistry C 13(3):985–1012. 10.1039/D4TC04166F

[43] Liu S, Tian J, Zhang W (2021) Fabrication and application of nanoporous anodic aluminum oxide: a review. Nanotechnology 32(22):222001. 10.1088/1361-6528/abe25f 10.1088/1361-6528/abe25f 33530076

[44] Santos A, Yoo JH, Rohatgi CV et al (2016) Realisation and advanced engineering of true optical rugate filters based on nanoporous anodic alumina by sinusoidal pulse anodisation. Nanoscale 8(3):1360–1373. 10.1039/C5NR05462A 26492584 10.1039/c5nr05462a

[45] Kushnir SE, Pchelyakova TY, Napolskii KS (2018) Anodizing with voltage versus optical path length modulation: a new tool for the preparation of photonic structures. Journal of Materials Chemistry C 6(45):12192–12199. 10.1039/C8TC04246B

[46] Law CS, Lim SY, Liu L et al (2020) Realization of high-quality optical nanoporous gradient-index filters by optimal combination of anodization conditions. Nanoscale 12(17):9404–9415. 10.1039/C9NR10526C 32124886 10.1039/c9nr10526c

[47] Lee W, Schwirn K, Steinhart M et al (2008) Structural engineering of nanoporous anodic aluminium oxide by pulse anodization of aluminium. Nat Nanotechnol 3(4):234–239. 10.1038/nnano.2008.54 18654508 10.1038/nnano.2008.54

[48] Lee W, Scholz R, Gösele U (2008) A continuous process for structurally well-defined Al2O3 nanotubes based on pulse anodization of aluminum. Nano Lett 8(8):2155–2160. 10.1021/nl080280x 18558786 10.1021/nl080280x

[49] Lee W, Kim J-C (2010) Highly ordered porous alumina with tailor-made pore structures fabricated by pulse anodization. Nanotechnology 21(48):485304. 10.1088/0957-4484/21/48/485304 21063054 10.1088/0957-4484/21/48/485304

[50] Apos, Sullivan JP, Wood GC et al (1997) The morphology and mechanism of formation of porous anodic films on aluminium. Proceedings of the Royal Society of London. A. Mathematical and Physical Sciences 317(1531):511–543. 10.1098/rspa.1970.0129

[51] Sulka GD, Parkoła KG (2007) Temperature influence on well-ordered nanopore structures grown by anodization of aluminium in sulphuric acid. Electrochim Acta 52(5):1880–1888. 10.1016/j.electacta.2006.07.053

[52] Lee W, Kim J-C, Gösele U (2010) Spontaneous current oscillations during hard anodization of aluminum under potentiostatic conditions. Adv Func Mater 20(1):21–27. 10.1002/adfm.200901213

[53] Law CS, Lim SY, Macalincag RM et al (2018) Light-confining nanoporous anodic alumina microcavities by apodized stepwise pulse anodization. ACS Applied Nano Materials 1(9):4418–4434. 10.1021/acsanm.8b00494

[54] Napolskii KS, Noyan AA, Kushnir SE (2020) Control of high-order photonic band gaps in one-dimensional anodic alumina photonic crystals. Opt Mater 109:110317. 10.1016/j.optmat.2020.110317

[55] Acosta LK, Bertó-Roselló F, Xifre-Perez E et al (2019) Stacked nanoporous anodic alumina gradient-index filters with tunable multispectral photonic stopbands as sensing platforms. ACS Appl Mater Interfaces 11(3):3360–3371. 10.1021/acsami.8b19411 30590008 10.1021/acsami.8b19411

[56] Santos A, Law CS, Pereira T et al (2016) Nanoporous hard data: optical encoding of information within nanoporous anodic alumina photonic crystals. Nanoscale 8(15):8091–8100. 10.1039/C6NR01068G 27020686 10.1039/c6nr01068g

[57] Lim SY, Law CS, Marsal LF et al (2018) Engineering of hybrid nanoporous anodic alumina photonic crystals by heterogeneous pulse anodization. Sci Rep 8(1):9455. 10.1038/s41598-018-27775-6 29930341 10.1038/s41598-018-27775-6 PMC6013466

[58] Liu L, Lim SY, Law CS et al (2021) Optical engineering of nanoporous photonic crystals by Gaussian-Like pulse anodization. Microporous Mesoporous Mater 312:110770. 10.1016/j.micromeso.2020.110770

[59] Law CS, Santos A, Nemati M et al (2016) Structural engineering of nanoporous anodic alumina photonic crystals by sawtooth-like pulse anodization. ACS Appl Mater Interfaces 8(21):13542–13554. 10.1021/acsami.6b03900 27171214 10.1021/acsami.6b03900

[60] Cheng W, Steinhart M, Gösele U et al (2007) Tree-like alumina nanopores generated in a non-steady-state anodization. J Mater Chem 17(33):3493–3495. 10.1039/B709618F

[61] Montero-Moreno JM, Belenguer M, Sarret M et al (2009) Production of alumina templates suitable for electrodeposition of nanostructures using stepped techniques. Electrochim Acta 54(9):2529–2535. 10.1016/j.electacta.2008.03.067

[62] Santos A, Ferré-Borrull J, Pallarès J et al (2011) Hierarchical nanoporous anodic alumina templates by asymmetric two-step anodization. physica status solidi (a) 208(3):668–674. 10.1002/pssa.201026435

[63] Kure-Chu S-Z, Osaka K, Yashiro H et al (2016) Facile fabrication of ordered multi-tiered hierarchical porous alumina nanostructures with multiple and fractional ratios of pore interval toward multifunctional nanomaterials. ECS Journal of Solid State Science and Technology 5(5):P285. 10.1149/2.0231605jss

[64] Nagaura T, Takeuchi F, Inoue S (2008) Fabrication and structural control of anodic alumina films with inverted cone porous structure using multi-step anodizing. Electrochim Acta 53(5):2109–2114. 10.1016/j.electacta.2007.09.016

[65] Santos A, Formentín P, Pallarès J et al (2011) Structural engineering of nanoporous anodic alumina funnels with high aspect ratio. J Electroanal Chem 655(1):73–78. 10.1016/j.jelechem.2011.02.005

[66] Li J, Li C, Chen C et al (2012) Facile method for modulating the profiles and periods of self-ordered three-dimensional alumina taper-nanopores. ACS Appl Mater Interfaces 4(10):5678–5683. 10.1021/am301603e 23020550 10.1021/am301603e

[67] Bruschi L, Mistura G, Phadungbut P et al (2015) Adsorption on ordered and disordered duplex layers of porous anodic alumina. Langmuir 31(17):4895–4905. 10.1021/acs.langmuir.5b00716 25871845 10.1021/acs.langmuir.5b00716

[68] Wang H, Hou S, Wang Q et al (2015) Dual-response for Hg2+ and Ag+ ions based on biomimetic funnel-shaped alumina nanochannels. Journal of Materials Chemistry B 3(8):1699–1705. 10.1039/C4TB01804D 32262442 10.1039/c4tb01804d

[69] Yu Z, Zhao K, Li L et al (2023) A vivid Au-porous anodic alumina composite film with the inverted taper structure for label-free detection. Nano Res 16(7):9997–10003. 10.1007/s12274-023-5549-6

[70] Santos A, Montero-Moreno JM, Bachmann J et al (2011) Understanding pore rearrangement during mild to hard transition in bilayered porous anodic alumina membranes. ACS Appl Mater Interfaces 3(6):1925–1932. 10.1021/am200139k 21539376 10.1021/am200139k

[71] Nemati M, Santos A, Losic D (2018) Fabrication and optimization of bilayered nanoporous anodic alumina structures as multi-point interferometric sensing platform. Sensors 18(2). 10.3390/s18020470 10.3390/s18020470 PMC5855889 29415436

[72] Macias G, Hernández-Eguía LP, Ferré-Borrull J et al (2013) Gold-coated ordered nanoporous anodic alumina bilayers for future label-free interferometric biosensors. ACS Appl Mater Interfaces 5(16):8093–8098. 10.1021/am4020814 23910449 10.1021/am4020814

[73] Jouault N, Berni S (2022) Synthesis of ordered duplex nanoporous alumina with modulated constriction and composition. Solid State Sci 133:107020. 10.1016/j.solidstatesciences.2022.107020

[74] Porta-i-Batalla M, Xifré-Pérez E, Eckstein C et al (2017) 3D nanoporous anodic alumina structures for sustained drug release. Nanomaterials 7(8). 10.3390/nano7080227 10.3390/nano7080227 PMC5575709 28825654

[75] Kim J, Jeong C (2024) Research on variation in nanopore parameters and surface characteristics of anodic aluminum oxide (AAO) films with time-controlled anodization processes. J Mater Sci 59(23):10556–10571. 10.1007/s10853-024-09806-y

[76] Roslyakov IV, Shirin NA, Evdokimov PV et al (2022) High-temperature annealing of porous anodic aluminium oxide prepared in selenic acid electrolyte. Surf Coat Technol 433:128080. 10.1016/j.surfcoat.2022.128080

[77] Janshoff A, Dancil K-PS, Steinem C et al (1998) Macroporous p-type silicon Fabry−Perot layers. Fabrication, Characterization, and Applications in Biosensing. Journal of the American Chemical Society 120(46):12108–12116. 10.1021/ja9826237

[78] Han H, Park S-J, Jang JS et al (2013) In situ determination of the pore opening point during wet-chemical etching of the barrier layer of porous anodic aluminum oxide: nonuniform impurity distribution in anodic oxide. ACS Appl Mater Interfaces 5(8):3441–3448. 10.1021/am400520d 23521656 10.1021/am400520d

[79] Ono S, Masuko N (2017) Effect of electric field strength on cell morphology and anion incorporation of anodic porous alumina. ECS Trans 75(27):23. 10.1149/07527.0023ecst

[80] Macias G, Ferré-Borrull J, Pallarès J et al (2014) 1-D nanoporous anodic alumina rugate filters by means of small current variations for real-time sensing applications. Nanoscale Res Lett 9(1):315. 10.1186/1556-276X-9-315 25024680 10.1186/1556-276X-9-315 PMC4082282

[81] Wang Y, Chen Y, Kumeria T et al (2015) Facile synthesis of optical microcavities by a rationally designed anodization approach: tailoring photonic signals by nanopore structure. ACS Appl Mater Interfaces 7(18):9879–9888. 10.1021/acsami.5b01885 25901537 10.1021/acsami.5b01885

[82] Yan P, Fei G-T, Li H et al (2014) Alumina photonic crystals with defect modes for sensor application. Chin J Chem Phys 27(1):121–124. 10.1063/1674-0068/27/01/121-124

[83] Huang K, Pu L, Shi Y et al (2006) Photoluminescence oscillations in porous alumina films. Appl Phys Lett 89(20):201118. 10.1063/1.2390645

[84] Vrublevsky I, Jagminas A, Hemeltjen S et al (2010) Photoluminescent behavior of heat-treated porous alumina films formed in malonic acid. Appl Surf Sci 256(7):2013–2017. 10.1016/j.apsusc.2009.09.038

[85] Santos A, Alba M, Rahman MM et al (2012) Structural tuning of photoluminescence in nanoporous anodic alumina by hard anodization in oxalic and malonic acids. Nanoscale Res Lett 7(1):228. 10.1186/1556-276X-7-228 22515214 10.1186/1556-276X-7-228 PMC3413565

[86] Santos A, Kumeria T, Losic D (2013) Optically optimized photoluminescent and interferometric biosensors based on nanoporous anodic alumina: a comparison. Anal Chem 85(16):7904–7911. 10.1021/ac401609c 23862775 10.1021/ac401609c

[87] Acosta LK, Law CS, Santos A et al (2022) Tuning intrinsic photoluminescence from light-emitting multispectral nanoporous anodic alumina photonic crystals. APL Photonics 7(2):026108. 10.1063/5.0078505

[88] Rajeev G, Prieto Simon B, Marsal LF et al (2018) Advances in nanoporous anodic alumina-based biosensors to detect biomarkers of clinical significance: a review. Adv Healthcare Mater 7(5):1700904. 10.1002/adhm.201700904 10.1002/adhm.201700904 29205934

[89] Jani AMM, Kempson IM, Losic D et al (2010) Dressing in layers: layering surface functionalities in nanoporous aluminum oxide membranes. Angew Chem Int Ed 49(43):7933–7937. 10.1002/anie.201002504 10.1002/anie.201002504 20845338

[90] Velleman L, Triani G, Evans PJ et al (2009) Structural and chemical modification of porous alumina membranes. Microporous Mesoporous Mater 126(1):87–94. 10.1016/j.micromeso.2009.05.024

[91] Losic D, Cole MA, Dollmann B et al (2008) Surface modification of nanoporous alumina membranes by plasma polymerization. Nanotechnology 19(24):245704. 10.1088/0957-4484/19/24/245704 21825829 10.1088/0957-4484/19/24/245704

[92] Chaki NK, Vijayamohanan K (2002) Self-assembled monolayers as a tunable platform for biosensor applications. Biosens Bioelectron 17(1):1–12. 10.1016/S0956-5663(01)00277-9 11742729 10.1016/s0956-5663(01)00277-9

[93] Dai J, Baker GL, Bruening ML (2006) Use of porous membranes modified with polyelectrolyte multilayers as substrates for protein arrays with low nonspecific adsorption. Anal Chem 78(1):135–140. 10.1021/ac0513966 16383320 10.1021/ac0513966

[94] Popat KC, Mor G, Grimes C et al (2004) Poly (ethylene glycol) grafted nanoporous alumina membranes. J Membr Sci 243(1):97–106. 10.1016/j.memsci.2004.05.030

[95] Radzik C, Kocanda GM, Haji-Sheikh M et al (2008) Electrical impedance response of a thick-thin film hybrid anodic nanoporous alumina sensor to methanol vapors. International Journal on Smart Sensing and Intelligent Systems 1(2):470–479. 10.21307/ijssis-2017-302

[96] Podgolin SK, Petukhov DI, Dorofeev SG et al (2020) Anodic alumina membrane capacitive sensors for detection of vapors. Talanta 219:121248. 10.1016/j.talanta.2020.121248 32887139 10.1016/j.talanta.2020.121248

[97] Park JY, Yi J-H, Choa Y-H (2023) ppb-Level ethanol gas sensor of porous anodic aluminum oxide at room temperature. J Am Ceram Soc 106(12):7209–7217. 10.1111/jace.19061

[98] Lim G-H, Kim I-Y, Park J-Y et al (2024) Anodic aluminum oxide-based chemi-capacitive sensor for ethanol gas. Nanomaterials 14(1). 10.3390/nano14010070 10.3390/nano14010070 PMC10780559 38202525

[99] Chen Y, Meng F, Li M et al (2009) Novel capacitive sensor: fabrication from carbon nanotube arrays and sensing property characterization. Sens Actuators, B Chem 140(2):396–401. 10.1016/j.snb.2009.04.012

[100] Timár-Horváth V, Juhász L, Vass-Várnai A et al (2008) Usage of porous Al2O3 layers for RH sensing. Microsyst Technol 14(7):1081–1086. 10.1007/s00542-007-0466-2

[101] Kim Y, Jung B, Lee H et al (2009) Capacitive humidity sensor design based on anodic aluminum oxide. Sens Actuators, B Chem 141(2):441–446. 10.1016/j.snb.2009.07.007

[102] Chung CK, Khor OK, Kuo EH et al (2020) Total effective surface area principle for enhancement of capacitive humidity sensor of thick-film nanoporous alumina. Mater Lett 260:126921. 10.1016/j.matlet.2019.126921

[103] Juhasz L, Vass-Vamai A, Timar-Horvath V et al, "Porous alumina based capacitive MEMS RH sensor," in *2008 Symposium on Design, Test, Integration and Packaging of MEMS/MOEMS*, 9–11 April 2008 2008, pp. 381–385, 10.1109/DTIP.2008.4753024.

[104] Yao L, Zheng M, Li H et al (2011) High-performance humidity sensors based on high-field anodized porous alumina films. Nanotechnology 22(37):379501. 10.1088/0957-4484/22/37/379501 10.1088/0957-4484/20/39/395501 19724104

[105] Sharma K, Islam SS (2016) Optimization of porous anodic alumina nanostructure for ultra high sensitive humidity sensor. Sens Actuators, B Chem 237:443–451. 10.1016/j.snb.2016.06.041

[106] Almasi Kashi M, Ramazani A, Abbasian H et al (2012) Capacitive humidity sensors based on large diameter porous alumina prepared by high current anodization. Sens Actuators, A 174:69–74. 10.1016/j.sna.2011.11.033

[107] Chung CK, Khor OK, Syu CJ et al (2015) Effect of oxalic acid concentration on the magnetically enhanced capacitance and resistance of AAO humidity sensor. Sens Actuators, B Chem 210:69–74. 10.1016/j.snb.2014.12.096

[108] Chen SW, Khor OK, Liao MW et al (2014) Sensitivity evolution and enhancement mechanism of porous anodic aluminum oxide humidity sensor using magnetic field. Sens Actuators, B Chem 199:384–388. 10.1016/j.snb.2014.03.057

[109] Jeong SH, Im HL, Hong S et al (2017) Massive, eco-friendly, and facile fabrication of multi-functional anodic aluminum oxides: application to nanoporous templates and sensing platforms. RSC Adv 7(8):4518–4530. 10.1039/C6RA25201J

[110] Chung CK, Ku CA, Wu ZE (2021) A high-and-rapid-response capacitive humidity sensor of nanoporous anodic alumina by one-step anodizing commercial 1050 aluminum alloy and its enhancement mechanism. Sens Actuators, B Chem 343:130156. 10.1016/j.snb.2021.130156

[111] Islam T, Nimal AT, Mittal U et al (2015) A micro interdigitated thin film metal oxide capacitive sensor for measuring moisture in the range of 175–625 ppm. Sens Actuators, B Chem 221:357–364. 10.1016/j.snb.2015.06.101

[112] Juhász L, Mizsei J (2009) Humidity sensor structures with thin film porous alumina for on-chip integration. Thin Solid Films 517(22):6198–6201. 10.1016/j.tsf.2009.04.010

[113] Balde M, Vena A, Sorli B (2015) Fabrication of porous anodic aluminium oxide layers on paper for humidity sensors. Sens Actuators, B Chem 220:829–839. 10.1016/j.snb.2015.05.053

[114] Alam N, Islam SS (2023) A graphene oxide (GO)–porous anodic alumina (PAA) bilayer system: how GO dispersion regulates the lower RH detection limit to near zero in conjugation with PAA. Journal of Materials Chemistry C 11(46):16297–16309. 10.1039/D3TC03075J

[115] Nahar RK (2000) Study of the performance degradation of thin film aluminum oxide sensor at high humidity. Sens Actuators, B Chem 63(1):49–54. 10.1016/S0925-4005(99)00511-0

[116] Kumar S, Islam T, Raina KK (2018) Study of long term drift of aluminum oxide thin film capacitive moisture sensor. IEEE Trans Device Mater Reliab 18(2):180–188. 10.1109/TDMR.2018.2813397

[117] Kumar S, Raina KK, Islam T (2021) Anodic aluminium oxide based humidity sensor for online moisture monitoring of power transformer. Sens Actuators, B Chem 329:128908. 10.1016/j.snb.2020.128908

[118] Vasiliev AA, Lipilin AS, Mozalev AM et al (2012) Gas sensors based on ceramic MEMS structures made of anodic alumina and yttria stabilized zirconia films. Additional Conferences (Device Packaging, HiTEC, HiTEN, and CICMT) 2012(CICMT):000528–000534. 10.4071/CICMT-2012-WP33

[119] Karpov EE, Karpov EF, Suchkov A et al (2013) Energy efficient planar catalytic sensor for methane measurement. Sens Actuators, A 194:176–180. 10.1016/j.sna.2013.01.057

[120] Bíró F, Dücső C, Radnóczi GZ et al (2017) ALD nano-catalyst for micro-calorimetric detection of hydrocarbons. Sens Actuators, B Chem 247:617–625. 10.1016/j.snb.2017.03.075

[121] Roslyakov IV, Kolesnik IV, Evdokimov PV et al (2021) Microhotplate catalytic sensors based on porous anodic alumina: Operando study of methane response hysteresis. Sens Actuators, B Chem 330:129307. 10.1016/j.snb.2020.129307

[122] Kalinin IA, Roslyakov IV, Bograchev D et al (2024) High performance microheater-based catalytic hydrogen sensors fabricated on porous anodic alumina substrates. Sens Actuators, B Chem 404:135270. 10.1016/j.snb.2023.135270

[123] Vasiliev AA, Pisliakov AV, Sokolov AV et al (2016) Non-silicon MEMS platforms for gas sensors. Sens Actuators, B Chem 224:700–713. 10.1016/j.snb.2015.10.066

[124] Nadekar B, Ghuge C, Khollam Y et al (2024) Boosting electrochromic performance of Pt-embedded porous alumina thick films via interface engineering with Low-temperature VOCs gas sensors. 10.2139/ssrn.4713835

[125] Varghese OK, Gong D, Dreschel WR et al (2003) Ammonia detection using nanoporous alumina resistive and surface acoustic wave sensors. Sens Actuators, B Chem 94(1):27–35. 10.1016/S0925-4005(03)00252-1

[126] Elam JW, Zinovev A, Han CY et al (2006) Atomic layer deposition of palladium films on Al2O3 surfaces. Thin Solid Films 515(4):1664–1673. 10.1016/j.tsf.2006.05.049

[127] Chung C-K, Ku C-A (2023) An effective resistive-type alcohol vapor sensor using one-step facile nanoporous anodic alumina. Micromachines 14(7). 10.3390/mi14071330 10.3390/mi14071330 PMC10384929 37512643

[128] Li C-Y, Li C-Y, Wu Y-L et al (2016) The fabrication of high sensitivity gold nanorod H2S gas sensors utilizing the highly uniform anodic aluminum oxide template. AIP Adv 6(12):125002. 10.1063/1.4971233

[129] Li C-Y, Hsu C-P, Li C-Y et al (2018) Synthesis of single-phase Au nanorods in an anodic aluminum oxide template with an optimized process for a highly sensitive and non-enzyme methyl mercaptan gas detector. Microsyst Technol 24(10):4129–4136. 10.1007/s00542-017-3667-3

[130] Mohd Chachuli SA, Hamidon MN, Mamat MS et al (2018) A hydrogen gas sensor based on TiO2 nanoparticles on alumina substrate. Sensors 18(8). 10.3390/s18082483 10.3390/s18082483 PMC6111410 30071579

[131] Zhang Y, Hang C, Jiang H et al (2025) Introducing oxygen vacancies into WO3 thin film for improving hydrogen sensing performance of Pd/WO3-x/AAO sensors. Sens Actuators, B Chem 423:136843. 10.1016/j.snb.2024.136843

[132] Lu C-C, Huang Y-S, Huang J-W et al (2010) A macroporous TiO2 oxygen sensor fabricated using anodic aluminium oxide as an etching mask. Sensors 10(1):670–683. 10.3390/s100100670 22315561 10.3390/s100100670 PMC3270862

[133] John N, Thomas P, Divya KV et al (2018) Enhanced room temperature gas sensing of aligned Mn3O4 nanorod assemblies functionalized by aluminum anodic membranes. Nanotechnology 29(33):335503. 10.1088/1361-6528/aac655 29781445 10.1088/1361-6528/aac655

[134] Kumar N, Srivastava AK, Nath R et al (2014) Probing the highly efficient room temperature ammonia gas sensing properties of a luminescent ZnO nanowire array prepared via an AAO-assisted template route. Dalton Trans 43(15):5713–5720. 10.1039/C3DT53305K 24557454 10.1039/c3dt53305k

[135] Lu S, Zhang Y, Liu J et al (2021) Sensitive H2 gas sensors based on SnO2 nanowires. Sens Actuators, B Chem 345:130334. 10.1016/j.snb.2021.130334

[136] Chen Y, Yuan T, Li Y et al (2024) Ppb-level H2S sensor with super selectivity based on Fe-NiOx nanotube assembled by AAO template. Ceramics International 50(13, Part A):22243–22251. 10.1016/j.ceramint.2024.01.320

[137] Yang C-S, Mahmood A, Kim B et al (2016) Enhancing gas sensing properties of graphene by using a nanoporous substrate. 2D Materials 3(1):011007. 10.1088/2053-1583/3/1/011007

[138] Sener M, Sisman O, Kilinc N (2023) AAO-assisted nanoporous platinum films for hydrogen sensor application. Catalysts 13(3). 10.3390/catal13030459

[139] Wang M, Feng Y (2007) Palladium–silver thin film for hydrogen sensing. Sens Actuators, B Chem 123(1):101–106. 10.1016/j.snb.2006.07.030

[140] Hassan K, Chung G-S (2017) Fast and reversible hydrogen sensing properties of Pd-capped Mg ultra-thin films modified by hydrophobic alumina substrates. Sens Actuators, B Chem 242:450–460. 10.1016/j.snb.2016.11.078

[141] Du L, Zheng L, Wei H et al (2019) Palladium/bismuth nanowires with rough surface for stable hydrogen sensing at low temperatures. ACS Applied Nano Materials 2(3):1178–1184. 10.1021/acsanm.8b02029

[142] Kyun Tae K, Jun S, Sung Min C (2006) Hydrogen gas sensor using Pd nanowires electro-deposited into anodized alumina template. IEEE Sens J 6(3):509–513. 10.1109/JSEN.2006.874456

[143] Hao M, Wu S, Zhou H et al (2016) Room-temperature and fast response hydrogen sensor based on annealed nanoporous palladium film. J Mater Sci 51(5):2420–2426. 10.1007/s10853-015-9555-2

[144] Ding D, Chen Z, Lu C (2006) Hydrogen sensing of nanoporous palladium films supported by anodic aluminum oxides. Sens Actuators, B Chem 120(1):182–186. 10.1016/j.snb.2006.02.007

[145] Rumiche F, Wang HH, Hu WS et al (2008) Anodized aluminum oxide (AAO) nanowell sensors for hydrogen detection. Sens Actuators, B Chem 134(2):869–877. 10.1016/j.snb.2008.06.054

[146] Kim B-J, Kim J-S (2014) Hydrogen sensor using the Pd film supported on anodic aluminum oxide. Int J Hydrogen Energy 39(29):16500–16505. 10.1016/j.ijhydene.2014.05.071

[147] Wu S, Zhou H, Hao M et al (2016) Fast response hydrogen sensors based on anodic aluminum oxide with pore-widening treatment. Appl Surf Sci 380:47–51. 10.1016/j.apsusc.2016.02.087

[148] Sanger A, Kumar A, Kumar A et al (2016) Highly sensitive and selective hydrogen gas sensor using sputtered grown Pd decorated MnO2 nanowalls. Sens Actuators, B Chem 234:8–14. 10.1016/j.snb.2016.04.152

[149] Gorokh G, Mozalev A, Solovei D et al (2006) Anodic formation of low-aspect-ratio porous alumina films for metal-oxide sensor application. Electrochim Acta 52(4):1771–1780. 10.1016/j.electacta.2006.01.081

[150] Huang JW, Chang CK, Lu KCC et al, "A novel approach for nanoporous gas sensor fabrication using anodic aluminum oxidation and MEMS process," in *2009 9th IEEE Conference on Nanotechnology (IEEE-NANO)*, 26–30 July 2009 2009, pp. 322–325.

[151] Lu C, Chen Z (2009) High-temperature resistive hydrogen sensor based on thin nanoporous rutile TiO2 film on anodic aluminum oxide. Sens Actuators, B Chem 140(1):109–115. 10.1016/j.snb.2009.04.004

[152] Shyue J-J, Cochran RE, Padture NP (2006) Transparent-conducting, gas-sensing nanostructures (nanotubes, nanowires, and thin films) of titanium oxide synthesized at near-ambient conditions. J Mater Res 21(11):2894–2903. 10.1557/jmr.2006.0352

[153] Zheng B-C, Shi J-B, Lin H-S et al (2020) Growth of less than 20 nm SnO nanowires using an anodic aluminum oxide template for gas sensing. Micromachines 11(2). 10.3390/mi11020153 10.3390/mi11020153 PMC7074593 32019256

[154] Kumar A, Sanger A, Kumar A et al (2016) Highly sensitive and selective CO gas sensor based on a hydrophobic SnO2/CuO bilayer. RSC Adv 6(52):47178–47184. 10.1039/C6RA06538D

[155] Gorokh G, Zakhlebayeva A, Taratyn I et al (2022) A micropowered chemoresistive sensor based on a thin alumina nanoporous membrane and SnxBikMoyOz nanocomposite. Sensors 22(10). 10.3390/s22103640 10.3390/s22103640 PMC9147226 35632047

[156] Yan J, Song Z (2024) Metal-decorated 3D tin oxide nanotubes in a monolithic sensor array chip for room-temperature gas identification. J Alloy Compd 976:173075. 10.1016/j.jallcom.2023.173075

[157] Ding D, Chen Z, Rajaputra S et al (2007) Hydrogen sensors based on aligned carbon nanotubes in an anodic aluminum oxide template with palladium as a top electrode. Sens Actuators, B Chem 124(1):12–17. 10.1016/j.snb.2006.11.034

[158] Hoa ND, Van Quy N, Cho Y et al (2007) An ammonia gas sensor based on non-catalytically synthesized carbon nanotubes on an anodic aluminum oxide template. Sens Actuators, B Chem 127(2):447–454. 10.1016/j.snb.2007.04.041

[159] Mangu R, Rajaputra S, Clore P et al (2010) Ammonia sensing properties of multiwalled carbon nanotubes embedded in porous alumina templates. Mater Sci Eng, B 174(1):2–8. 10.1016/j.mseb.2010.03.003

[160] Sudheep CV, Verma A, Jasrotia P et al (2024) Revolutionizing gas sensors: the role of composite materials with conducting polymers and transition metal oxides. Results in Chemistry 7:101255. 10.1016/j.rechem.2023.101255

[161] Farea MA, Mohammed HY, Shirsat SM et al (2021) Hazardous gases sensors based on conducting polymer composites: review. Chem Phys Lett 776:138703. 10.1016/j.cplett.2021.138703

[162] Dan Y, Cao Y, Mallouk TE et al (2007) Dielectrophoretically assembled polymer nanowires for gas sensing. Sens Actuators, B Chem 125(1):55–59. 10.1016/j.snb.2007.01.042

[163] Biring S, Kolaru RB (2022) Achieving high response of poly (3-hexylthiophene-2,5-diyl) molecules to gaseous ammonia using anodic aluminum oxide nanoporous substrate operated under 1 V. Sens Actuators, B Chem 373:132712. 10.1016/j.snb.2022.132712

[164] Sanger A, Jain PK, Mishra YK et al (2017) Palladium decorated silicon carbide nanocauliflowers for hydrogen gas sensing application. Sens Actuators, B Chem 242:694–699. 10.1016/j.snb.2016.11.107

[165] Koh H-J, Kim SJ, Maleski K et al (2019) Enhanced selectivity of MXene gas sensors through metal ion intercalation: in situ X-ray diffraction study. ACS Sensors 4(5):1365–1372. 10.1021/acssensors.9b00310 31062965 10.1021/acssensors.9b00310

[166] Laatar F, Harizi A, Zarroug A et al (2017) Novel CdSe nanorods/porous anodic alumina nanocomposite-based ethanol sensor: sensitivity enhancement by visible light illumination. J Mater Sci: Mater Electron 28(16):12259–12267. 10.1007/s10854-017-7042-z

[167] Vázquez RM, Mozalev A, Llobet E (2014) Fast response hydrogen microsensor based on semiconductor niobium-oxide nanostructures via smart anodizing of Al/Nb metal layers. Procedia Engineering 87:811–814. 10.1016/j.proeng.2014.11.674

[168] Mozalev A, Vázquez RM, Bittencourt C et al (2014) Formation–structure–properties of niobium-oxide nanocolumn arrays via self-organized anodization of sputter-deposited aluminum-on-niobium layers. Journal of Materials Chemistry C 2(24):4847–4860. 10.1039/C4TC00349G

[169] Mozalev A, Bendova M, Vazquez RM et al (2016) Formation and gas-sensing properties of a porous-alumina-assisted 3-D niobium-oxide nanofilm. Sens Actuators, B Chem 229:587–598. 10.1016/j.snb.2016.02.024

[170] Mozalev A, Calavia R, Vázquez RM et al (2013) MEMS-microhotplate-based hydrogen gas sensor utilizing the nanostructured porous-anodic-alumina-supported WO3 active layer. Int J Hydrogen Energy 38(19):8011–8021. 10.1016/j.ijhydene.2013.04.063

[171] Vergara A, Calavia R, Vázquez RM et al (2012) Multifrequency interrogation of nanostructured gas sensor arrays: a tool for analyzing response kinetics. Anal Chem 84(17):7502–7510. 10.1021/ac301506t 22834982 10.1021/ac301506t

[172] Tang W, Chen Z, Song Z et al (2022) Microheater integrated nanotube array gas sensor for parts-per-trillion level gas detection and single sensor-based gas discrimination. ACS Nano 16(7):10968–10978. 10.1021/acsnano.2c03372 35797450 10.1021/acsnano.2c03372

[173] Artzi-Gerlitz R, Benkstein KD, Lahr DL et al (2009) Fabrication and gas sensing performance of parallel assemblies of metal oxide nanotubes supported by porous aluminum oxide membranes. Sens Actuators, B Chem 136(1):257–264. 10.1016/j.snb.2008.10.056

[174] Huang J-W, Lu KC-C, Huang Y-S et al (2009) Novel fabrication process using nanoporous anodic aluminum oxidation and MEMS technologies for gas detection. Procedia Chemistry 1(1):56–59. 10.1016/j.proche.2009.07.014

[175] Pytlicek Z, Bendova M, Prasek J et al (2019) On-chip sensor solution for hydrogen gas detection with the anodic niobium-oxide nanorod arrays. Sens Actuators, B Chem 284:723–735. 10.1016/j.snb.2019.01.009

[176] Lee B, Cho I, Kang M et al (2023) Thermally/mechanically robust anodic aluminum oxide (AAO) microheater platform for low power chemoresistive gas sensor. J Micromech Microeng 33(8):085011. 10.1088/1361-6439/ace05e

[177] Dickey EC, Varghese OK, Ong KG et al (2002) Room temperature ammonia and humidity sensing using highly ordered nanoporous alumina films. Sensors 2(3):91–110. 10.3390/s20300091

[178] Kocanda M, Haji-Sheikh MJ, Ballantine DS (2009) Detection of cyclic volatile organic compounds using single-step anodized nanoporous alumina sensors. IEEE Sens J 9(7):836–841. 10.1109/JSEN.2009.2022553

[179] Abrego I, Campos A, Bethancourt G et al (2012) A study of anodization time and voltage effect on the fabrication of self-ordered nano porous aluminum oxide films: a gas sensor application. MRS Online Proc Libr 1449(1):73–79. 10.1557/opl.2012.1171

[180] Vunnam S, Andolu A, Kocanda M et al, "Oxygen detection using nanoporous anodized aluminum oxide sensors," in *2011 Fifth International Conference on Sensing Technology*, 28 Nov.-1 Dec. 2011 2011, pp. 112–115, 10.1109/ICSensT.2011.6136943.

[181] Tao W, Pan D, Gong Z et al (2018) Nanoporous platinum electrode grown on anodic aluminum oxide membrane: Fabrication, characterization, electrocatalytic activity toward reactive oxygen and nitrogen species. Anal Chim Acta 1035:44–50. 10.1016/j.aca.2018.06.076 30224143 10.1016/j.aca.2018.06.076

[182] Zia TuH, Ali Shah AuH (2023) Polypyrrole/porous anodized aluminum oxide composite device for ammonia gas sensing. Synth Met 296:117350. 10.1016/j.synthmet.2023.117350

[183] Mohammadi M, Fardindoost S, Iraji zad A, et al (2020) Room temperature selective sensing of aligned Ni nanowires using impedance spectroscopy. Materials Research Express 7(2):025044. 10.1088/2053-1591/ab66ac

[184] Zhou X, Xue Z, Chen X et al (2020) Nanomaterial-based gas sensors used for breath diagnosis. Journal of Materials Chemistry B 8(16):3231–3248. 10.1039/C9TB02518A 32031564 10.1039/c9tb02518a

[185] Kumar V, Raghuwanshi SK, Kumar S (2022) Advances in nanocomposite thin-film-based optical fiber sensors for environmental health monitoring—a review. IEEE Sens J 22(15):14696–14707. 10.1109/JSEN.2022.3185004

[186] Wang L, Qin X, Ji D et al (2015) Engineering optical properties of metal/porous anodic alumina films for refractometric sensing. Appl Surf Sci 355:139–144. 10.1016/j.apsusc.2015.07.087

[187] Vasilkov MY, Mikhailov IN, Nikulin YV et al (2023) Spectral optical properties of ceramic nanoporous membranes based on anodic aluminium oxide coated silver in ammonia vapors. Opt Spectrosc 131(8):771–776. 10.1134/S0030400X23060188

[188] Casanova F, Chiang CE, Li CP et al (2008) Effect of surface interactions on the hysteresis of capillary condensation in nanopores. Europhys Lett 81(2):26003. 10.1209/0295-5075/81/26003

[189] Casanova F, Chiang CE, Li C-P et al (2008) Gas adsorption and capillary condensation in nanoporous alumina films. Nanotechnology 19(31):315709. 10.1088/0957-4484/19/31/315709 21828801 10.1088/0957-4484/19/31/315709

[190] Lim SY, Law CS, Tran KN et al (2023) Engineering nanoporous anodic alumina bilayered interferometers for liquid and gas sensing. ACS Applied Nano Materials 6(22):20954–20969. 10.1021/acsanm.3c03978

[191] Kumeria T, Parkinson L, Losic D (2011) A nanoporous interferometric micro-sensor for biomedical detection of volatile sulphur compounds. Nanoscale Res Lett 6(1):634. 10.1186/1556-276X-6-634 22176687 10.1186/1556-276X-6-634 PMC3265559

[192] Kumeria T, Losic D (2011) Reflective interferometric gas sensing using nanoporous anodic aluminium oxide (AAO). physica status solidi (RRL) – Rapid Research Letters 5(10–11):406–408. 10.1002/pssr.201105425

[193] Martín J, Martín-González M, Francisco Fernández J et al (2014) Ordered three-dimensional interconnected nanoarchitectures in anodic porous alumina. Nat Commun 5(1):5130. 10.1038/ncomms6130 25342247 10.1038/ncomms6130 PMC4770565

[194] Santos A, Law CS, Chin Lei DW et al (2016) Fine tuning of optical signals in nanoporous anodic alumina photonic crystals by apodized sinusoidal pulse anodisation. Nanoscale 8(43):18360–18375. 10.1039/C6NR06796D 27766342 10.1039/c6nr06796d

[195] Lee J, Bae K, Kang G et al (2015) Graded-lattice AAO photonic crystal heterostructure for high Q refractive index sensing. RSC Adv 5(88):71770–71777. 10.1039/C5RA15890G

[196] Shang GL, Fei GT, Zhang Y et al (2013) Preparation of narrow photonic bandgaps located in the near infrared region and their applications in ethanol gas sensing. Journal of Materials Chemistry C 1(34):5285–5291. 10.1039/C3TC30782D

[197] Tran KN, Tran HNQ, Gunenthiran S et al (2023) Desorption kinetics profiling of volatile organic compounds in nanoporous anodic alumina photonic crystal optical microcavities. ACS Applied Optical Materials 1(12):1987–2003. 10.1021/acsaom.3c00320

[198] Tran KN, Tran HNQ, Lim SY et al (2024) Detection of volatile organic compounds through spectroscopic signatures in nanoporous fabry–pérot optical microcavities. ACS Appl Mater Interfaces 16(19):24961–24975. 10.1021/acsami.4c03804 38706267 10.1021/acsami.4c03804

[199] Tran HNQ, Tran KN, Gunenthiran S et al (2024) Tailoring tamm plasmon resonances in dielectric nanoporous photonic crystals. ACS Appl Mater Interfaces 16(9):11787–11799. 10.1021/acsami.3c16981 38394678 10.1021/acsami.3c16981

[200] Liu Y, Wang HH, Indacochea JE et al (2011) A colorimetric sensor based on anodized aluminum oxide (AAO) substrate for the detection of nitroaromatics. Sens Actuators, B Chem 160(1):1149–1158. 10.1016/j.snb.2011.09.040

[201] Markovics Á, Kovács B (2014) Optical ammonia sensors for environmental applications. Anal Lett 47(3):465–477. 10.1080/00032719.2013.843188

[202] Lee L-R, Karapala VK, Lin Y-L et al (2020) Intelligent environmental sensing: fabrication of switchable, reusable, and highly sensitive gas sensors with spiropyran-grafted anodic aluminum oxide templates. The Journal of Physical Chemistry C 124(22):11870–11876. 10.1021/acs.jpcc.0c01129

[203] Kim SW, Cha SH, Kang BH et al, "Optical gas sensor based on LSPR using ZnO nanoparticles and AAO nanostructure," in *2015 IEEE SENSORS*, 1–4 Nov. 2015 2015, pp. 1–3, 10.1109/ICSENS.2015.7370399.

[204] Proença M, Lednický T, Meira DI et al (2024) New parameter for benchmarking plasmonic gas sensors demonstrated with densely packed Au nanoparticle layers. ACS Appl Mater Interfaces 16(42):57832–57842. 10.1021/acsami.4c11102 39399975 10.1021/acsami.4c11102 PMC11503611

[205] Han J, Zhang X, Jones RR et al (2024) Dual-functional Au-porous anodic alumina (PAA) sensors for enrichment and label-free detection of airborne virus with surface-enhanced Raman scattering. Build Environ 257:111484. 10.1016/j.buildenv.2024.111484

[206] Oh M-K, Kim H, Gupta P et al (2024) High sensitive SERS film of Ag-coated aggregated nanowire structure and benzene gas detection. Japanese Journal of Applied Physics 63(1):015003. 10.35848/1347-4065/ad160b

[207] Fujiwara Y, Okura I, Miyashita T et al (2002) Optical oxygen sensor based on fluorescence change of pyrene-1-butyric acid chemisorption film on an anodic oxidation aluminium plate. Anal Chim Acta 471(1):25–32. 10.1016/S0003-2670(02)00928-5

[208] Fujiwara Y, Amao Y (2003) Optical oxygen sensor based on controlling the excimer formation of pyrene-1-butylic acid chemisorption layer onto nano-porous anodic oxidized aluminium plate by myristic acid. Sens Actuators, B Chem 89(1):58–61. 10.1016/S0925-4005(02)00428-8

[209] Araki N, Amao Y, Funabiki T et al (2007) Optical oxygen-sensing properties of porphyrin derivatives anchored on ordered porous aluminium oxide plates. Photochem Photobiol Sci 6(7):794–803. 10.1039/b618030b 17609774 10.1039/b618030b

[210] Liu C-Y, Sadhu AS, Karmakar R et al (2022) Strongly Improving the sensitivity of phosphorescence-based optical oxygen sensors by exploiting nano-porous substrates. Biosensors 12(10). 10.3390/bios12100774 10.3390/bios12100774 PMC9599114 36290912

[211] Yelton WG, Pfeifer KB, Staton AW (2002) Porous Al2 O 3 nanogeometry sensor films: growth and analysis. J Electrochem Soc 149(1):H1. 10.1149/1.1421608

[212] Chen X, Guo Z, Huang J et al (2008) Fabrication of gas ionization sensors using well-aligned MWCNT arrays grown in porous AAO templates. Colloids Surf, A 313–314:355–358. 10.1016/j.colsurfa.2007.04.118

[213] Song Z, Fang W, Zhu B et al (2024) Nano-Schottky-junction-engineered Pd/SnO2 nanotube array for ultrasensitive hydrogen sensing at room temperature. Anal Methods 16(35):5954–5958. 10.1039/D4AY00988F 39188154 10.1039/d4ay00988f

[214] Zhang D, Sun Y, Wang Z et al (2023) Switchable biomimetic nanochannels for on-demand SO2 detection by light-controlled photochromism. Nat Commun 14(1):1901. 10.1038/s41467-023-37654-y 37019894 10.1038/s41467-023-37654-y PMC10076267

[215] Markovics Á, Nagy G, Kovács B (2009) Reflection-based sensor for gaseous ammonia. Sens Actuators, B Chem 139(1):252–257. 10.1016/j.snb.2009.02.075

[216] Markovics Á, Kovács B (2013) Fabrication of optical chemical ammonia sensors using anodized alumina supports and sol–gel method. Talanta 109:101–106. 10.1016/j.talanta.2013.01.054 23618145 10.1016/j.talanta.2013.01.054

